# Protein covariation networks for elucidating ferroptosis inducer mechanisms and potential synergistic drug targets

**DOI:** 10.1038/s42003-025-07886-3

**Published:** 2025-03-31

**Authors:** Rina Kunishige, Yoshiyuki Noguchi, Naomi Okamoto, Lei Li, Akito Ono, Masayuki Murata, Fumi Kano

**Affiliations:** 1 Multimodal Cell Analysis Collaborative Research Cluster, Institute of Science Tokyo, Yokohama-shi, Kanagawa Japan; 2 Cellshoot Therapeutics, Inc., Koto-ku, Tokyo Japan; 3 International Research Center for Neurointelligence, Institutes for Advanced Study, The University of Tokyo, Tokyo, Japan; 4 Axcelead Drug Discovery Partners, Inc., Fujisawa, Kanagawa Japan; 5 Cell Biology Center, Institute of Integrated Research, Institute of Science Tokyo, Yokohama-shi, Kanagawa Japan

**Keywords:** Target identification, Single-cell imaging, Cell death, Protein analysis, Cellular signalling networks

## Abstract

In drug development, systematically characterizing a compound’s mechanism of action (MoA), including its direct targets and effector proteins, is crucial yet challenging. Network-based approaches, unlike those focused solely on direct targets, effectively detect a wide range of cellular responses elicited by compounds. This study applied protein covariation network analysis, leveraging quantitative, morphological, and localization features from immunostained microscopic images, to elucidate the MoA of AX-53802, a novel ferroptosis inducer. From the candidate targets extracted through network analysis, GPX4 was verified as the direct target by validation experiments. Additionally, aggregates involving GPX4, TfR1, and F-actin were observed alongside iron reduction, suggesting a ferroptosis defense mechanism. Furthermore, combination therapies targeting GPX4 and FAK/Src were found to enhance cancer cell death, and MDM2, ezrin, and cortactin were identified as potential ferroptosis inhibitor targets. These findings highlight the effectiveness of network-based approaches in uncovering a compound’s MoA and developing combination therapies for cancer.

## Abstract (web summary)

Using protein covariation network analysis, this study uncovers the mechanism of action for AX-53802, a new ferroptosis inducer, validating GPX4 as its direct target, and proposes combination therapies targeting FAK/Src to enhance cancer cell death.

## Introduction

In drug development, systematically characterizing a compound’s mechanism of action (MoA), including not only its direct targets but also effector proteins influencing its pharmacological effects, is pivotal yet challenging^1^. The high failure rate of compounds in clinical trials, often due to toxicity or lack of efficacy, underscores the necessity of assessing both on-target compound activity and potential side effects. Although the primary approach involves identifying direct targets through methods such as direct binding assays^2–4^ or chemoinformatics analysis designed to assess compound MoA similarities or specific compound–target interactions^1,5–7^, these methods may overlook crucial indirect effectors. Conversely, network-based methods can detect a broad spectrum of cellular responses triggered by a compound. For example, methods such as detecting MoA by network dysregulation (DeMAND)^1^ or Mode of Action by Network Analysis^8,9^, allows for MoA estimation from network perturbation analysis based on drug-specific gene expression profiles (GEPs). DeMAND identifies a compound’s target based on downstream molecular consequences, even in cases where the expression of the target itself shows minimal change^1^. Although comprehensive analysis of gene expression data is valuable, compounds primarily interact with proteins, indicating that direct detection of protein-level changes likely provides a clearer understanding of the MoA compared with indirect gene expression changes. Moreover, although network perturbation analysis based on GEP data may offer insights into MoA at the pathway level, it may fail to identify the precise molecular targets or effectors within those pathways, leaving a critical knowledge gap.

To address this issue, we aimed to elucidate the MoA directly through protein-level network analysis. For this purpose, we employed a protein covariation network analysis method termed protein localization and modification-based covariation network (PLOM-CON) analysis^10^, based on proteins’ quantitative and morphological features as well as their localization in immunostained microscopy images. The resulting network captures the rapid, synchronized, time-dependent behaviors of target proteins, incorporating localization and morphological data that are inaccessible through biochemical methods. Originally developed to analyze transient condensates induced by specific stimuli^10^, this method is capable of comprehensively capturing dynamic cellular processes. In the present study, we leverage this method to delineate the MoA of a novel ferroptosis inducer (FIN) identified through compound screening.

Among image-based network analysis methods, Difference in Sums of Weighted cO-dependence profiles (DiSWOP)^11^ employs multiplex staining, such as MELC^12^ and requires image alignment for single-cell analysis, which can be labor-intensive. In comparison, PLOM-CON has a simplified workflow in which individual cells are separately stained for each protein. While DiSWOP is well-suited for analyzing heterogeneous tissue samples, the ability of PLOM-CON to detect temporal changes in drug responses makes its results more interpretable and ideal for investigating drug mechanisms in homogeneous cultured cell systems (detailed comparisons are available in the Supplementary Discussion).

Ferroptosis, a form of regulated cell death characterized by iron-dependent lipid peroxidation, is regulated by various factors, including the cellular metabolism of iron, lipids, and reactive oxygen species^13–15^. It is initiated by the failure of the cellular antioxidant defense system, particularly the glutathione-dependent enzyme glutathione peroxidase 4 (GPX4), responsible for neutralizing lipid peroxides^16–19^. Given the relevance of ferroptosis to diseases such as cancer, neurodegenerative diseases, and tissue damage following ischemia-reperfusion events, it represents a major target for therapeutic interventions^14^.

Existing FINs can be categorized based on their MoA^16,20,21^. Class I inhibitors, such as erastin, inhibit the cystine/glutamate antiporter (system x_c_^−^), reducing cystine uptake and thereby depleting intracellular glutathione (GSH), a critical antioxidant^13,16,22–24^. Class II inhibitors, including RSL3 and ML162, covalently bind to selenocysteine at position 46 in GPX4’s active site, hindering its ability to neutralize lipid hydroperoxides^16,18,19^. Class III FINs, such as FIN56, degrade GPX4 and deplete coenzyme Q10, disrupting cellular antioxidant defenses and promoting ferroptosis^25^. Class IV FINs promote lipid peroxidation by increasing labile iron pool levels or directly oxidizing iron^26^. In cancer therapy, direct inhibition of GPX4 is proposed as a superior strategy for inducing ferroptosis in cancer cells compared with disrupting GSH^27^, despite challenges such as poor selectivity and insufficient in vivo antitumor effects^16,28,29^. To address these challenges, various strategies are being explored, including developing covalent GPX4 inhibitors using masked nitrile-oxide electrophiles (e.g., ML210)^17^, employing antibody-drug conjugates^30^, and leveraging PROTAC technology^31,32^. Beyond enhancing the efficiency and specificity of GPX4 inhibition, another promising direction in cancer therapy involves promoting cell death through combination therapies. Network analysis has emerged as an optimal strategy for exploring combination drug targets, allowing the identification of potential positive and negative regulators of ferroptosis from networks. Targeting these regulators for combination therapy could expedite and potentiate cancer cell death, potentially overcoming effectiveness issues associated with existing ferroptosis inducers.

In this study, we explored the MoA of a new ferroptosis inducer by comparing networks with and without the drug. Through PLOM-CON analysis and validation experiments, GPX4 was identified as the primary target. We also observed GPX4 forming aggregates with transferrin receptor protein 1 (TfR1) and filamentous actin (F-actin) as a downstream cellular response to lipid peroxidation, concurrent with iron reduction, indicative of a defensive mechanism against ferroptosis. From the network, we extracted potential combination drug targets using two methods, based on the assumption that the molecules most significantly altered in response to the ferroptosis inducer are likely ferroptosis regulators. Modulating the extracted targets could effectively facilitate or inhibit ferroptosis, highlighting the value of adopting a network-based approach for elucidating effective combination therapies.

## Results

### AX-53802, identified through compound screening, induces ferroptosis

**Fig. 1:**
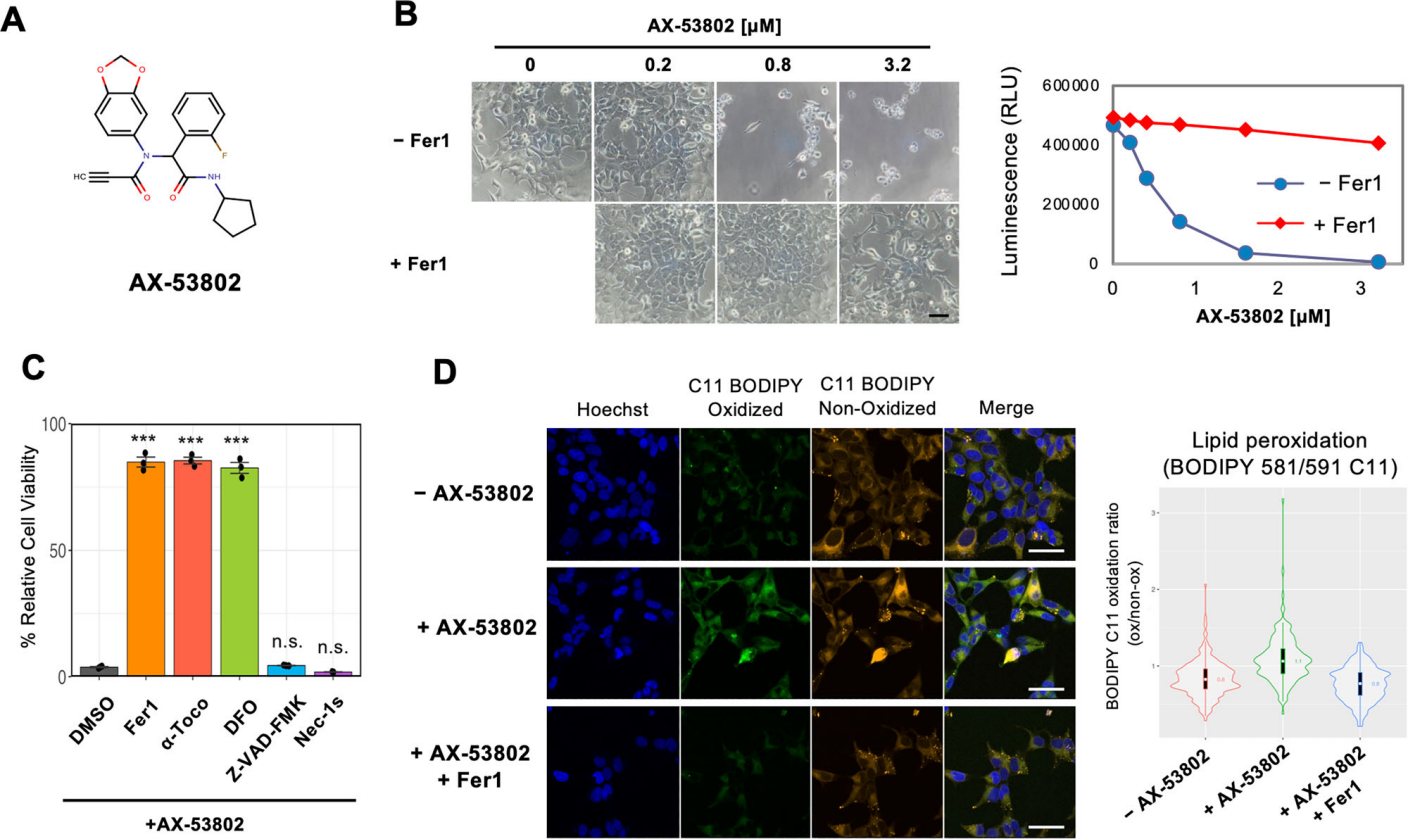
AX-53802, identified through compound screening, induces ferroptosis. **A** Chemical structure of AX-53802. **B** Microscopic image and viability measurements of HEK293 cells treated with various AX-53802 concentrations for 24 h, assessed through the CellTiter-Glo assay. Cotreatment with Fer1 (1 μM) rescued the effects of AX-53802 on cell viability. Scale bar: 40 μm. **C** Viability of HEK293 cells treated with 0.4 μM AX-53802 and cell death inhibitors, measured through the CellTiter-Glo assay. Cotreatment with the lipophilic antioxidants Fer1 (1 μM) or tocopherol (α-Toco; 100 μM), or with the iron chelator deferoxamine (DFO; 20 μM) inhibited cell death, whereas apoptosis (z-vad-fmk; 20 μM) and necrosis (Nec-1s; 10 μM) inhibitors did not. *Y*-axis: relative cell viability normalized to conditions lacking AX-53802 treatment. Data: means ± standard errors of means (SEMs; *n*  =  3). ***P  <  0.001; ns not significant (Dunnett’s test). **D** HEK293 cells treated with 0.8 μM AX-53802 for 1 h show lipid peroxide accumulation via BODIPY-C11 probe assessment. Cotreatment with Fer1 (1 μM) reduces oxidation. Scale bars: 50 μm. Right panel: quantification of BODIPY-C11 oxidation ratio per cell. Boxes, 25th–75th percentiles; center (white dots), median; whiskers, 1.5 × interquartile range. Absolute value of the effect size was calculated between conditions using Cliff’s Delta: −AX-53802 vs. +AX-53802, 0.54; −AX-53802 vs. +AX-53802 + Fer1,0.15; +AX-53802 vs. +AX-53802 + Fer1, 0.64.

Through screening >50,000 maximally structurally diverse compounds, aimed at identifying ferroptosis inducers using renal cancer cell lines, AX-53802 was identified (Fig. 1A). Its cytotoxic effect was effectively countered by cotreatment with ferroptosis inhibitor ferrostatin-1 (Fer1). Subsequent validation using HEK293 and HT-1080 cells confirmed significant induction of ferroptosis by AX-53802, suggesting a consistent MoA across different cell lines (Figs. 1B and S1). Given our expertise in immunofluorescence and microscopic image analysis using HEK293 cells, these cells were selected for further investigation.

To elucidate the mode of cell death induced by AX-53802, the ability of various cell death inhibitors with distinct mechanisms to suppress AX-53802–induced cell death was assessed. Cotreatment with the lipophilic antioxidants Fer1 or tocopherol, as well as the iron chelator deferoxamine, effectively inhibited AX-53802–induced cell death (Figs. 1C, S2, and S3). However, inhibitors of apoptosis (z-vad-fmk) and necrosis (Nec-1s) had no effect, confirming that AX-53802 induced ferroptotic cell death. Time-lapse observations further characterized the cell death process, revealing cellular swelling and rupture, typical features of ferroptosis (Supplementary Videos S1 and S2). In contrast to the synchronized wave-like cell death observed with typical ferroptosis inducers, such as erastin^33^, AX-53802–treated cells did not exhibit synchronized death, indicating AX-53802’s distinct MoA in ferroptosis induction. Time-lapse observations revealed morphological changes, such as cellular rounding beginning as early as 1 h after AX-53802 addition, with some cells dying as early as 3 h post-treatment. By 18 h, the majority of cells had undergone cell death, although a subset remained viable, highlighting the variability in cellular responses to ferroptosis induction.

Given that lipid peroxide accumulation is a hallmark of ferroptosis, it was measured using the BODIPY-C11 probe^34^. AX-53802 addition resulted in increased lipid peroxides detected as early as 1 h post-treatment, and this effect was suppressed by Fer1 treatment (Fig. 1D). Thus, these experiments confirmed AX-53802’s role as a ferroptosis inducer.

### Estimating the MoA of AX-53802 through PLOM-CON analysis

**Fig. 2:**
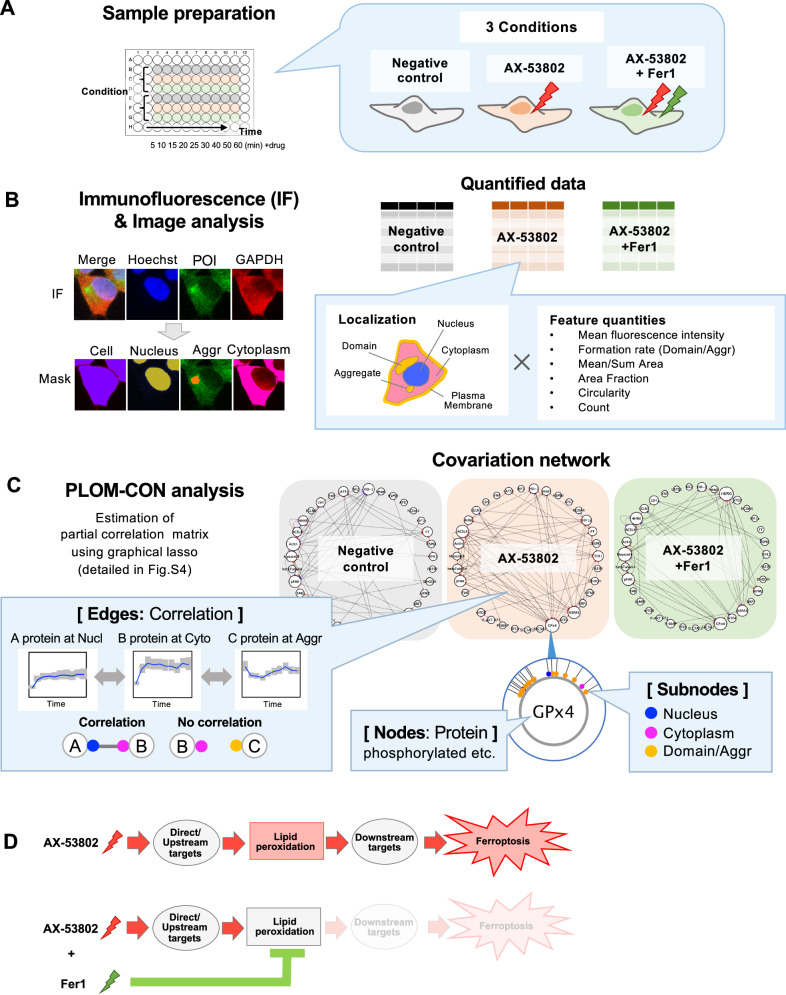
Schematic overview of PLOM-CON analysis for estimating the AX-53802 MoA. [Illustrations adapted with modifications from Noguchi et al. (2021), iScience, 24(7)]. **A** HEK293 cells in 96-well plates treated under three distinct conditions; untreated (control), 0.8 μM AX-53802, and AX-53802 + 1 μM Fer1. Cells were fixed at 5, 10, 15, 20, 25, 30, 40, 50, and 60 min post-treatment. **B** Immunofluorescence and image analysis were conducted following (**A**). Antibodies listed in Supplementary Data 1 were used for staining, followed by imaging using a confocal laser-scanning microscope (×40 objective). Segmentation was performed, with Hoechst and glyceraldehyde 3-phosphate dehydrogenase (GAPDH) staining used as nuclear and cell region markers, respectively. Specific masks were created for areas with intense localized staining: “domains” (relatively large), “aggregates” (small), and PM-associated aggregates. Following segmentation, feature quantities were collected, as detailed in Supplementary Data 2, including fluorescence intensity across cellular compartments (nucleus, cytoplasm, domain, aggregates, and PM), domain and aggregate formation rates, and morphological characteristics, e.g., area and circularity. **C** Covariation network constructed using feature quantities from (**B**). Partial correlation matrix, viewed as a weighted adjacency matrix, was estimated using the graphical lasso algorithm and visualized as networks (refer to Fig. S5). In the network, nodes represent proteins of interest and subnodes are color-coded by the cellular localizations (nucleus, cytoplasm, domains, and aggregates) of feature quantities. Edges between nodes indicate temporal correlations between protein feature quantities within each localization. **D** PLOM-CON analysis was conducted under three conditions: negative control (untreated cells), AX-53802 treatment, and AX-53802 + Fer1 cotreatment. Fer1 counteracts lipid peroxide accumulation, offering insights into protein changes preceding or following lipid peroxide formation in the pathway.

To elucidate the mechanism underlying AX-53802’s ferroptosis induction, we employed PLOM-CON analysis (Fig. 2A–C; Schematic overview)^10^. This method involves capturing time-series microscopy images of samples stained with antibodies against numerous proteins, quantifying their abundance, post-translational modification levels, localization, and morphological features, and constructing a network to illustrate their temporal correlations.

For PLOM-CON analysis, we examined three conditions: untreated cells as controls, cells treated with AX-53802, and cells treated with both AX-53802 and Fer1. By including Fer1 in the latter condition, we aimed to inhibit lipid peroxide accumulation, thereby determining specific protein alterations occurring relative to lipid peroxidation in the cellular pathway (Fig. 2D). Time points (5, 10, 15, 20, 25, 30, 40, 50, and 60 min) were selected up to just before significant morphological changes occurred in the cells (Supplementary Video S1). This strategy aimed to capture early and subtle protein dynamics changes before noticeable morphological alterations interfered with accurate fluorescence intensity quantification during image analysis.

We selected antibodies for PLOM-CON analysis based on two criteria. First, antibodies targeting molecules pivotal to various ferroptosis-related pathways with confirmed localizations from reliable sources, such as the Human Protein Atlas and antibody manufacturers’ data. This validation ensured the specificity and reliability of our observations. Second, antibodies were chosen based on their responsiveness to AX-53802–induced changes, resulting in a curated selection of 35 antibodies (refer to Fig. S4 for representative staining images and Supplementary Data 1 for details on the functions of the target proteins and the usage of the antibodies). Antibodies spanned various critical pathways: the GPX4/GSH pathway^13,16^ (SLC7A11, SLC3A2, GPX4, HSP90^35^, ATF4, and HSPA5^36^), the FSP1/DHODH-CoQ10 pathway (FSP1^37^, ACSL4^38^, FATP2, and DHODH^39,40^), lipid peroxidation markers (4HNE^41^), antioxidant stress response^42^ (Nrf2, HO-1, Keap1, HIF1a, EGLN1, and LSH), iron metabolism (TfR1^43^, HSPB1, FT^44,45^, NCOA4^46^, and ATG7^45^), the Hippo pathway^47^ (NF2, LATS1, and YAP), the mTOR pathway^48^ (mTOR, pAkt, and pS6rp), lysosome-related molecules^35^ (TFEB and LAMP2), and cytoskeleton/adhesion-related molecules^49^ [F-actin (phalloidin), myosin IIB, beta-tubulin, pFAK, and FAK]. Stained images showed intense localized staining of several antibodies in areas within cells (Fig. S4).

**Fig. 3:**
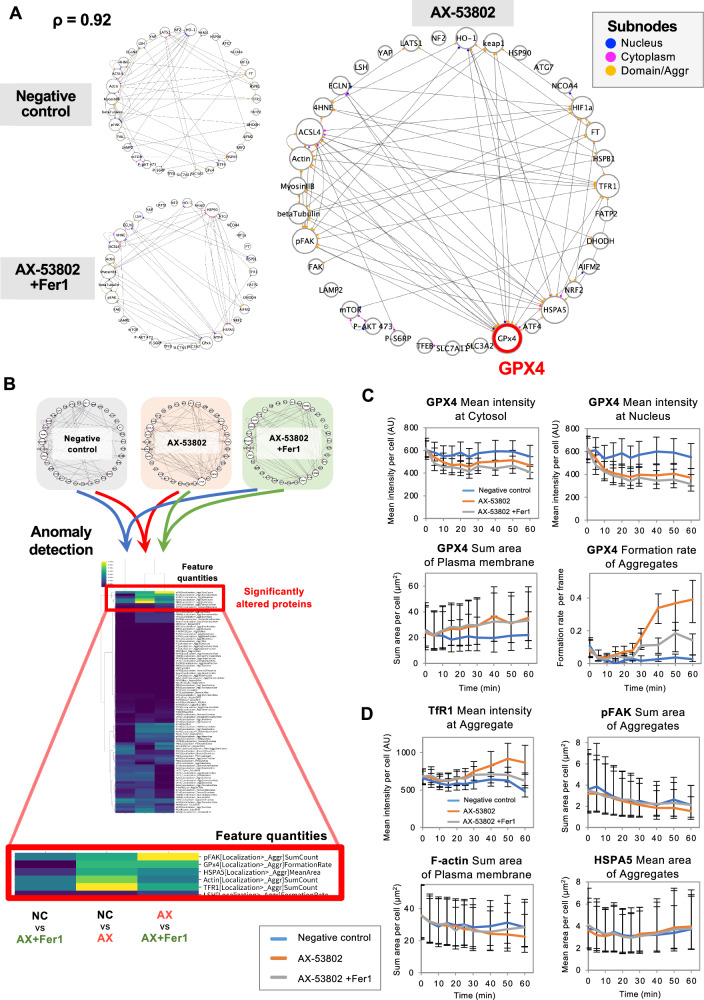
Cell state differences across drug treatment conditions visualized through covariation networks. **A** Covariation networks for three treatment conditions at *ρ* = 0.92 (refer to Fig. S6 for networks with varying *ρ* values). Network of 0.8 μM AX-53802–treated cells exhibited increased edges compared with the negative control network, with GPX4 exhibiting the highest number of edges. Edges between nodes indicate temporal correlations between protein feature quantities in each localization. Subnodes are color-coded by the cellular localizations of feature quantities: blue, nucleus; pink, cytoplasm; orange, domains, and aggregates. **B** Heatmap visualizing correlation anomaly scores (*ρ* = 0.92). Higher values indicate more significant alterations in connectivity and correlation strength of a node’s first neighbors (directly connected nodes). Feature quantities of pFAK, GPX4, HSPA5, F-actin, and TfR1 exhibited substantial variations between conditions (magnified area outlined in red). Enlarged heatmaps with other ρ values are shown in Fig. S10. **C** Line graph showing temporal dynamics of GPX4 feature quantities. Mean fluorescence intensity in the cytoplasm and nucleus decreased, whereas PM fluorescence increased within 10 min of AX-53802 treatment. These changes were unaffected by Fer1 cotreatment. Proportion of cells forming aggregates increased 30 min after AX-53802 addition, with partial reversal observed upon Fer1 cotreatment. Data: medians with interquartile ranges (error bars). **D** Line graph showing the temporal dynamics of TfR1, pFAK, F-actin, and HSPA5 feature quantities. The mean fluorescence intensity of TfR1 at aggregates increased 30 min after AX-53802 addition, attenuated by Fer1. The fluorescent area of pFAK aggregates and F-actin at the cell membrane slightly decreased after AX-53802 addition, with Fer1 showing a trend toward reversal. Data: medians with interquartile ranges (error bars).

In our image analysis, we included standard nuclear and cytoplasmic regions along with these localized staining regions, categorized as large “domains,” small “aggregates,” and plasma membrane (PM)-associated aggregates (Fig. 2B). Supplementary Data 2 details these features, including fluorescence intensity across cellular compartments, rates of domain/aggregate formation, and morphological characteristics, such as area and circularity. Following comprehensive data collection, we calculated partial correlation matrices culminating in network visualization. The PLOM-CON method employs the graphical lasso algorithm^50–52^ to estimate the partial correlation matrix, which may be viewed as a weighted adjacency matrix for covariation network visualization (Fig. S5). The resulting network comprises nodes representing proteins of interest, subnodes color-coded by cellular localization (nucleus, cytoplasm, domains, and aggregates), and edges between nodes indicating correlations between protein feature quantities for each localization (Fig. 2C). The regularization parameter ρ in the graphical lasso method controls network sparsity, with higher ρ values yielding fewer edges. As a representative example, Fig. 3A shows the network at *ρ* = 0.92, and networks for varying ρ values are presented in Fig. S6.

Through network comparisons considering three conditions, namely with and without AX-53802 as well as AX-53802 combined with Fer1, it was evident that networks with AX-53802 exhibited a higher number of edges, indicating extensive AX-53802–triggered protein covariations. Interestingly, this complex network of interactions was simplified upon Fer1 treatment, suggesting a partial reversal of AX–53802–induced changes. Among the proteins (nodes), GPX4 exhibited the most significant differences, showing the highest number of edges under AX-53802 treatment (Fig. 3A). HSPA5, TfR1, HIF1α, ACSL4, and pFAK also showed notable variations, undergoing changes in connectivity and partners across different conditions. Specific feature quantities of connected nodes observed under each condition as well as their temporal changes shown in heatmaps are provided in Figs. S7–S9.

To further explore quantitative differences between the correlation networks, we employed the correlation anomaly score^52^, which reflects changes in the connectivity and correlation strength of a node’s first neighbors (directly connected nodes), with larger values indicating more significant changes. Visualized via a heatmap of correlation anomaly scores (Figs. 3B and S10), notable changes in the feature quantities of pFAK, GPX4, HSPA5, F-actin, and TfR1 were observed between conditions. These changes predominantly occurred between the control and AX-53802 treatment conditions as well as between the sole AX-53802 treatment and AX-53802 combined with Fer1. Conversely, transitions between the control and rescue conditions (AX-53802 + Fer1) were less pronounced, indicating that these feature quantities are influenced by AX-53802 and rescued by Fer1.

Focusing on proteins exhibiting changes between conditions, we examined the temporal dynamics of their specific feature quantities. This can be achieved by referencing the temporal data that underpin the PLOM-CON analysis. By examining the timing of specific changes in time-course graphs, we can identify relationships between upstream and downstream proteins. For GPX4, we observed a decrease in mean fluorescence intensity in both the cytoplasm and nucleus shortly after AX-53802 introduction (within 10 min), accompanied by increased fluorescence at the PM (Fig. 3C). This suggests GPX4’s membrane translocation, which was not rescued by Fer1, indicating that this change occurs upstream of lipid peroxidation. Additionally, aggregate formation increased over time, starting around 30 min after AX-53802 treatment, and this effect was partially reversed by Fer1, implicating lipid peroxidation in mediating aggregate formation (Fig. 3C).

Considering other proteins with significant correlation anomaly scores, we focused on TfR1, exhibiting an increase in the average fluorescence intensity of aggregates 30 min after AX-53802 addition, a change rescued by Fer1 (Fig. 3D; further details regarding TfR1 assessment are provided in the next section). pFAK showed a decrease in the fluorescent area of aggregates, likely reflecting reduced focal adhesion (punctate staining) concurrent with cell rounding induced by AX-53802, also rescued by Fer1 (Fig. 3D). For F-actin, a decrease in the cell membrane fluorescent area following AX-53802 treatment was observed, with Fer1 showing a trend toward reversing this effect. Conversely, HSPA5 showed minimal differences between conditions (Fig. 3D), and validation experiments with an HSPA5 inhibitor confirmed that the MoA of AX-53802 is distinctly different from the mechanism of HSPA5 inhibition (Fig. S11), leading to its exclusion from further analysis.

Collectively, these findings underscore the pivotal role of GPX4 upstream in AX-53802’s MoA, with the enzyme demonstrating marked early temporal changes (decreases in the nucleus/cytoplasm and an increase at the PM) not mitigated by Fer1.

### GPX4 is the direct target of AX-53802

**Fig. 4:**
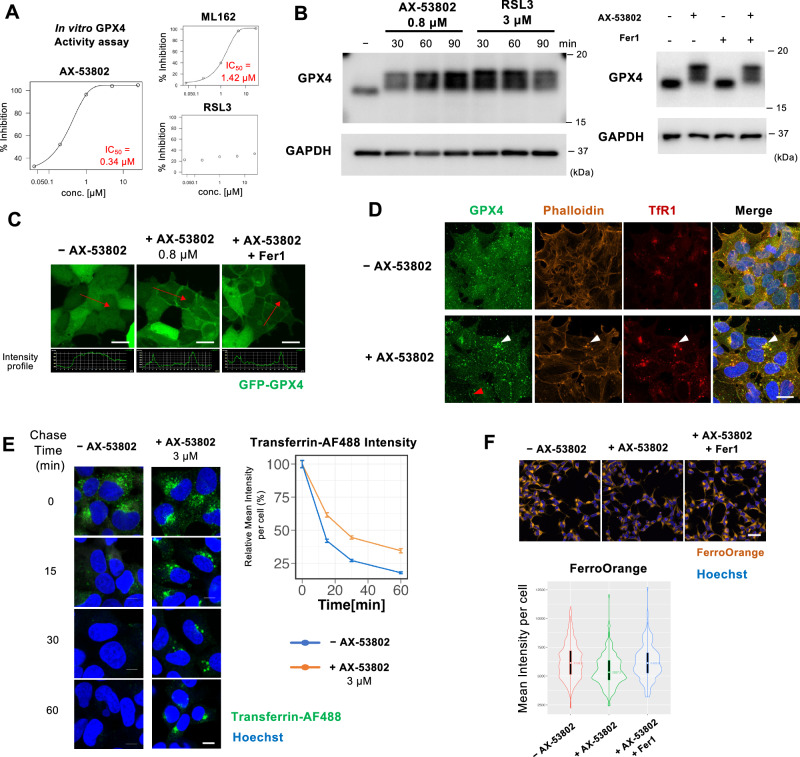
GPX4 is the direct target of AX-53802. **A** In vitro inhibition assay showing AX-53802 and ML162 activity against GPX4, with IC_50_ values of 0.34 μM and 1.42 μM, respectively. RSL3 demonstrated a modest inhibitory effect in vitro, with negligible concentration dependency. **B** Western blot showing GPX4 band shift following AX-53802 and RSL3 treatment, 30 min post-treatment (left panel). Fer1 cotreatment did not rescue this shift (right panel). **C** Images of HEK293 cells expressing GFP-GPX4, untreated or treated with 0.8 μM AX-53802 for 1 h, showing AX-53802–induced GPX4 membrane translocation, unaffected by Fer1 cotreatment. Intensity profiles shown below indicate fluorescence signal along the red arrows. Scale bars: 20 μm. Supplementary Video S3 provides time-lapse data. **D** Immunofluorescence images of HEK293 cells costained with anti-GPX4, phalloidin, and anti-TfR1 antibodies, observed with confocal microscopy (×60 objective). Treatment with 0.8 μM AX-53802 for 1 h induced aggregate formation (comprising GPX4, F-actin, and TfR1) near the nucleus. HEK293 cells were plated on glass-based dishes for high-resolution imaging at high magnification. Scale bar: 20 μm. **E** Pulse-chase experiments using fluorescently labeled transferrin (transferrin-AF488), with or without AX-53802 treatment. Cells were pretreated with 3 μM AX-53802 for 30 min, pulse-labeled with 10 μg/mL transferrin-AF488 (green) for 30 min, and chased with 10 μg/mL unlabeled transferrin for the indicated times. Transferrin recycling was significantly delayed with AX-53802 treatment; labeled transferrin persisted near the nucleus after 60 min, contrasting with rapid recycling under control conditions. Scale bars: 10 μm. Right panel: quantification of mean transferrin-AF488 intensity per cell, normalized to 0 min intensity. Effect sizes were calculated for each time point as the absolute value of Cliff’s Delta: 0.016, 0.562, 0.690, and 0.528. **F** Quantification of labile iron levels in untreated HEK293 cells or those treated with 0.8 μM AX-53802 for 1 h. Post-treatment, cells were incubated with FerroOrange probe for 30 min. Labile iron content decreased under AX-53802 treatment, an effect rescued by Fer1 addition. Scale bar: 50 μm. Right panel: quantification of mean FerroOrange intensity per cell. Absolute values of effect size were calculated between conditions using Cliff’s Delta: −AX-53802 vs. +AX-53802, 0.31; −AX-53802 vs. +AX-53802 + Fer1, 0.03; +AX-53802 vs. +AX-53802 + Fer1, 0.29.

Network analysis and examination of the actual temporal dynamics of feature quantities revealed GPX4 as the most upstream protein among the 35 ferroptosis-related proteins studied as part of AX-53802’s MoA. To explore the possibility that GPX4 is a direct target of AX-53802, we conducted in vitro experiments using the GPX4 enzyme and its substrate. Results confirmed that AX-53802 directly inhibits GPX4 activity independently of other cellular components, akin to other inhibitors, such as ML162 (Fig. 4A). The half-maximal inhibitory concentration (IC_50_) for AX-53802 was 0.34 µM, highlighting its potency as a GPX4 inhibitor compared with ML162 (IC_50_: 1.42 µM).

Given that the GPX4 inhibitors RSL3 and ML162 form covalent bonds with the 46th selenocysteine of GPX4 via an α-chloroacetamide reactive warhead^17,53,54^, we investigated whether AX-53802 also forms a covalent bond with GPX4. Despite lacking a chloroacetamide group, AX-53802 features a propiolamide group, suggesting its potential for forming a Michael addition–type covalent bond^55^ (Fig. 1A). Covalent inhibitors of GPX4 typically induce a band shift in western blot (WB) analysis^55,56^. Upon WB analysis of AX-53802, a band shift was observed similar to that of RSL3; this change was unaffected by Fer1, indicating AX-53802’s probable covalent bonding with GPX4 (Fig. 4B).

To validate AX-53802–induced membrane translocation of GPX4, as suggested earlier by temporal change data (Fig. 3C), time-lapse observations were conducted on HEK293 cells stably expressing GFP-GPX4. Membrane translocation was observed within 5–10 min of AX-53802 addition, a phenomenon not rescued by Fer1 (Fig. 4C and Supplementary Videos S3 and S4). Similar membrane transitions were observed with other covalent GPX4 inhibitors, such as RSL3 and ML162, distinguishing them from other classes of inducers (Fig. S12). These results confirm that AX-53802 forms a covalent bond with GPX4, initiating membrane translocation directly upon binding, rather than through lipid peroxide generation. This conclusion is supported by image quantification data from our network analysis (Fig. 3C), providing a clearer understanding of AX-53802’s MoA.

Next, we explored the formation of aggregates, as suggested by the temporal changes observed in GPX4. Many protein features identified through correlation anomaly analysis were associated with aggregates; therefore, we investigated whether these aggregates represented identical or distinct entities within cells. Containing experiments using anti-GPX4 and anti-TfR1 antibodies as well as phalloidin with AX-53802 addition revealed colocalization of GPX4, F-actin, and TfR1 proteins, forming aggregates near the nucleus (Fig. 4D). Given the presence of TfR1 in aggregates, suggesting perturbation in transferrin recycling, we conducted pulse-chase experiments using fluorescently labeled transferrin (transferrin-AF488). Results revealed a significant delay in transferrin recycling in the presence of AX-53802, with labeled transferrin persisting near the nucleus even after 60 min, contrasting with rapid recycling observed under control conditions (Fig. 4E and Supplementary Videos S5 and S6). To assess the impact on cellular iron levels, labile iron content was quantified using the FerroOrange probe^57^, revealing a decrease with AX-53802 treatment, evident from reduced FerroOrange fluorescence, which was rescued by Fer1 addition (Figs. 4F and  S13).

These findings highlight AX-53802’s direct targeting of GPX4, inhibiting its activity through a covalent bond. Moreover, we identified an upstream event, independent of lipid peroxides, wherein AX-53802–bound GPX4 is translocated to the cell membrane. Subsequently, the formation of aggregates containing GPX4, TfR1, and F-actin was observed. This aggregate formation correlated with delayed transferrin recycling and subsequent reduction in intracellular free iron levels. These results suggest the presence of a cellular defense mechanism against ferroptosis, wherein aggregate formation acts as a stress response to counteract ferroptosis.

### Target discovery for combination therapy using network analysis

**Fig. 5:**
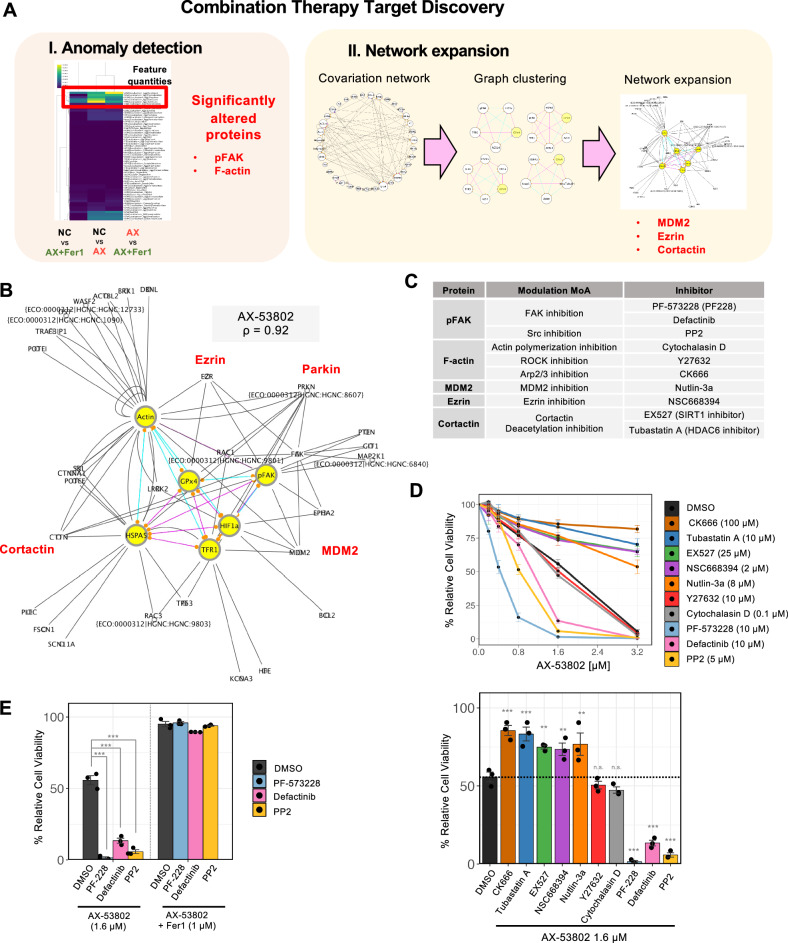
Target discovery for combination therapy using network analysis. **A** Schematic overview of two strategies for combination therapy target extraction. The first strategy incorporates correlation anomaly score (Fig. 3B), highlighting pFAK and F-actin as candidates. The second approach combined graph clustering (Fig. S14) with network expansion, identifying MDM2, ezrin, and cortactin as candidates. **B** Example of a densely connected cluster in the AX-53802 network at *ρ* = 0.92 extracted via graph clustering (yellow nodes), followed by network expansion using a protein–protein interaction database (BioGrid) to target first neighbors of cluster components. MDM2, ezrin, cortactin, and Parkin emerged as potential combination therapy targets. Refer to Fig. S15 for other clusters extracted from *ρ* = 0.92 networks. **C** Table of selected inhibitors for targeting the candidate proteins for combination therapy. **D** Viability measurements of HEK293 cells cotreated with various AX-53802 concentrations and inhibitors targeting combination therapy candidates for 24 h, assessed via the CellTiter-Glo assay. Inhibitors concentrations did not cause proliferation when used alone: CK666 (100 μM), tubastatin A (10 μM), EX527 (25 μM), NSC668394 (2 μM), nutlin-3a (8 μM), Y27632 (10 μM), cytochalasin D (0.1 μM), PF-573228 (10 μM), defactinib (10 μM), and PP2 (5 μM). Compared with AX-53802 alone, tubastatin A, EX527, NSC668394, nutlin-3a, and CK666 mitigated cell death, whereas PF-573228, defactinib, and PP2 facilitated cell death. Cytochalasin D and Y27632 did not significantly alter viability. Lower bar graph shows data from cells cotreated with 1.6 μM AX-53802. Y-axis: relative cell viability normalized to conditions lacking AX-53802 treatment. Data: means ± SEMs (*n*  =  3). **P < 0.01; ***P  <  0.001; ns not significant (Dunnett’s test). **E** Viability measurements of HEK293 cells cotreated with 1.6 μM AX-53802 and PF-573228 (10 μM), defactinib (10 μM), or PP2 (5 μM), rescued by 1 μM Fer1. *Y*-axis: relative cell viability normalized to conditions lacking AX-53802 treatment. Data: means ± SEMs (*n*  =  3). ***P  <  0.001 (Tukey’s test).

PLOM-CON network analysis not only identifies direct targets affected by AX-53802 but also reveals downstream changes in proteins involved in ferroptosis and the cellular responses contributing to its resistance. Recognizing the potential inclusion of both positive and negative regulators of ferroptosis among these changes, we identified candidates for combination therapy. This approach could reveal new strategies for efficient ferroptosis induction in cancer cells or ferroptosis prevention in acute kidney injury and neurodegenerative diseases, among other conditions. We employed two strategies for target protein extraction: a correlation anomaly–based method and a network expansion–based method for extensive target searching (Fig. 5A).

The correlation anomaly–based strategy highlighted four molecules (excluding the direct target GPX4), namely pFAK, HSPA5, F-actin, and TfR1, with significant connectivity changes between the control and AX-53802–altered networks (Fig. 3B). Among these, pFAK and F-actin were selected as potential drug targets for combination therapy, with HSPA5 omitted owing to its minimal change in feature quantity (Fig. 3D), and TfR1 excluded given the predictable outcome that inhibiting it would reduce iron levels and thereby suppress ferroptosis.

For the network expansion–based approach, we initially identified densely connected clusters in the AX-53802–conditioned network through graph clustering^58^ (Fig. S14), followed by expansion of these clusters using common protein-protein interaction (PPI) data from the BioGrid database^59^, targeting the first neighbors of the cluster components (Fig. S15). Expansion was filtered based on three criteria: (i) proteins sharing common Gene Ontology (GO) terms with those enriched in the extracted clusters, indicating possible functional alignment; (ii) proteins sharing edges with over 50% of proteins with high correlation anomaly scores in the cluster; and (iii) proteins meeting the first two criteria under multiple regularization parameters, ensuring robust selection. This filtering process isolated promising ferroptosis regulators having PPIs with proteins strongly influenced by AX-53802 and implicated in cellular functions adversely affected by AX-53802, as delineated by specific GO terms. Consequently, the proteins MDM2, ezrin, cortactin, and Parkin emerged as potential combination therapy targets (Fig. 5B). Given the lack of available agents to modulate Parkin activity, we further explored MDM2, ezrin, and cortactin as prime candidates.

The two approaches yielded five potential combination therapy targets: pFAK, F-actin, MDM2, ezrin, and cortactin. To modulate these targets’ activities, we used various inhibitors (Fig. 5C): PF-573228 (PF-228) and defactinib for direct FAK targeting, and the Src inhibitor PP2 as an upstream FAK inhibitor. For actin modulation, we employed the polymerization inhibitor cytochalasin D, the Arp2/3 complex inhibitor CK666, and the ROCK inhibitor Y27632 to manage the dynamic structure of actin fibers. MDM2 was targeted directly with nutlin-3a, ezrin directly with NSC668394, and cortactin indirectly (given the lack of direct inhibitors) with EX527, and tubastatin A, inhibitors of the upstream deacetylases SIRT1 and HDAC6, respectively^60,61^. Inhibition of cortactin deacetylases was anticipated to increase acetylated cortactin levels, leading to the nuclear localization of cortactin and its diminished interaction with F-actin^62,63^.

We combined AX-53802 at varying concentrations (0, 0.2, 0.4, 0.8, 1.6, and 3.2 µM) with the aforementioned inhibitors (used at concentrations not inhibiting proliferation when employed alone) and measured cell viability after 24 h (Fig. 5D). Compared with the concentration-dependent viability decrease caused by AX-53802 alone, drugs that attenuated ferroptosis-induced viability loss included tubastatin A, EX527, NSC668394, nutlin-3a, and CK666, whereas those that exacerbated ferroptosis, including PF-228, defactinib, and PP2, decreased viability at lower AX-53802 concentrations. Neither cytochalasin D nor Y27632 significantly affected viability. Cell death induced by cotreatment with PF-228, defactinib, and PP2 was rescued by Fer1, confirming the cell death type as ferroptosis (Fig. 5E).

Considering some drugs’ ferroptosis-suppressing effects may arise from direct antioxidant activity, we conducted a 2,2-diphenyl-1-picrylhydrazyl (DPPH) antioxidant assay^64^ (Fig. S16). Only tubastatin A displayed mild antioxidant activity comparable to that of 40 μg/mL Trolox; other drugs exhibited no antioxidant activity. Correspondingly, lipid peroxidation levels assessed via BODIPY-C11 were partially suppressed by tubastatin A but not by NSC66839 or nutlin-3a (Fig. S17). These findings suggest that NSC66839-mediated ezrin inhibition and nutlin-3a-mediated MDM2 inhibition suppress ferroptosis downstream of lipid peroxidation production. Notably lacking direct antioxidant activity, EX527 inhibited lipid peroxidation, suggesting that SIRT1 inhibition suppresses ferroptosis, at least partially, upstream of lipid peroxidation.

### Co-administration of AX-53802 and FAK/Src inhibitors promotes cell death

**Fig. 6:**
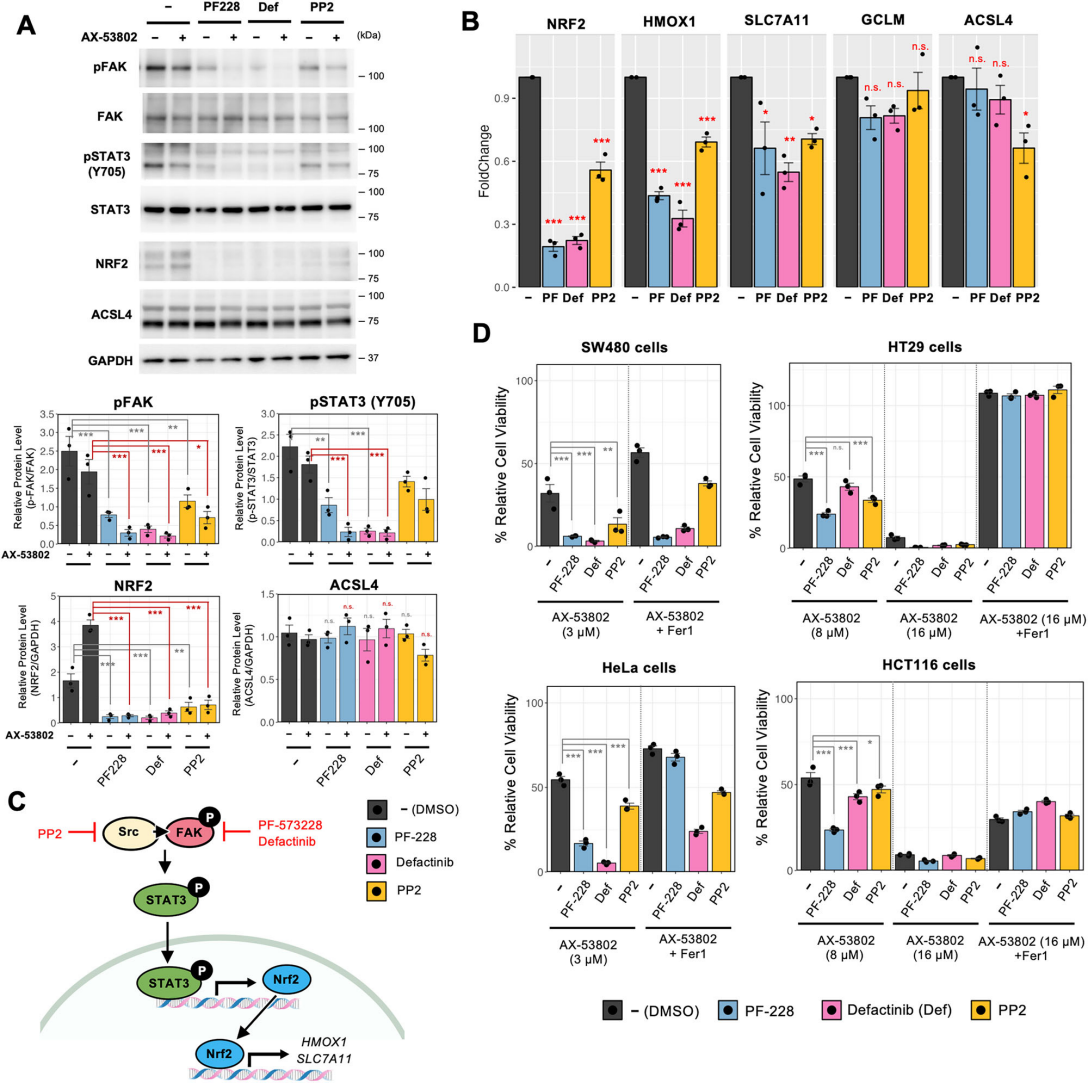
AX-53802 and FAK/Src inhibitor cotreatment promotes cell death. **A** Western blot showing reduced FAK phosphorylation and Nrf2 protein levels upon FAK/Src inhibitor treatment. STAT3 also exhibited decreased phosphorylation, albeit not statistically significant for the Src inhibitor PP2. No change in ACSL4 protein levels was observed. Data: means ± SEMs (*n*  =  3). *P < 0.05; **P < 0.01; ***P  <  0.001; ns: not significant (Tukey’s test). **B** Real-time PCR analysis of *NRF2*, *HMOX1*, *SLC7A11*, *GCLM*, and *ACSL4* in cells treated with FAK/Src inhibitors for 3 h. Transcription of NRF2 and its downstream transcriptional control target genes, *HMOX1* and *SLC7A11*, was significantly reduced by FAK/Src inhibitor treatment. GCLM showed a decreasing trend. Data: means ± SEMs (*n*  =  3). *P < 0.05; **P < 0.01; ***P  <  0.001; ns not significant (Dunnett’s test). **C** Schematic representation of the proposed pathway facilitating ferroptosis through FAK/Src inhibitor action. **D** Viability of cancer cell lines SW480, HT29, HCT116, and HeLa cotreated with AX-53802 and FAK/Src inhibitors for 24 h, measured via the CellTiter-Glo assay. FAK/Src inhibitors induced increased cell death in SW480 and HeLa cells. Cotreatment with PF-228 enhanced cell death in HT29 and HCT116 cells. Fer1 rescued cell death in HT29 cells, with incomplete rescue observed in other cells. Data: means ± SEMs (*n*  =  3). *P < 0.05; **P < 0.01; ***P  <  0.001; ns not significant (Tukey’s test).

Next, we investigated the synergistic effects of combining AX-53802 with specific FAK/Src inhibitors, namely PF-228, defactinib, and PP2, on ferroptosis and explored their MoA. Prior research indicated that α6β4 integrin can protect against erastin-induced ferroptosis by suppressing ACSL4 expression mediated by Src and STAT3, revealing complex interactions between cellular adhesion molecules and ferroptosis pathways^65^. Therefore, we inhibited FAK/Src in HEK293 cells, assessing the effects on STAT3 activity and ACSL4 expression. Results revealed reduced STAT3 phosphorylation, with no increase in ACSL4 transcription levels (Fig. 6A); thus, we investigated alternative pathways. Specifically, we explored the role of Nrf2, a critical regulator of cellular antioxidative responses, the transcription of which is regulated by STAT3^66,67^. Under conditions of FAK/Src inhibition, we observed decreased Nrf2 RNA and protein levels (Fig. 6A, B), along with downregulation of several Nrf2 target genes^68^, including *HMOX1* and *SLC7A11*, suggesting that FAK/Src inhibition blocks the Nrf2 antioxidative stress response (Fig. 6C).

We extended our investigation to various cancer cell lines, including SW480, HT29, HCT116, and HeLa, to assess the generalizability of our findings. At AX-53802 concentrations that reduced viability to ~50%, cotreatment with FAK/Src inhibitors markedly increased cell death induction, particularly noticeable in SW480 and HeLa cells (Figs. 6D and S18). In HT29 and HCT116 cells, PF-228 produced a significant effect, whereas defactinib and PP2 showed limited efficacy (Figs. 6D and S18). The incomplete rescue by Fer1 in some cells suggests the involvement of additional cell death mechanisms, warranting further investigation into the cell death induction mechanisms. These results confirm that although response variability exists among different cell lines, FAK/Src inhibitors, particularly PF-228, generally enhance cancer cell death.

## Discussion

PLOM-CON analysis focuses on protein localization and post-translational modifications, offering substantial advantages over traditional genetic networks. In this study, we leveraged PLOM-CON analysis to elucidate the MoA of a newly identified ferroptosis inducer. In the network, protein intracellular localization information was represented by color-coded subnodes, facilitating visualization of correlations between protein features (e.g., nucleus, cytoplasm, and intracellular aggregates) among covarying proteins. In the obtained AX-53802–treated network, GPX4 was found to connect with other proteins through feature quantities in the nucleus, aggregates, and PM. By examining the actual temporal dynamics of GPX4 features, we identified the process wherein AX-53802 binding initially triggers GPX4 membrane translocation followed by aggregate formation. The network also revealed increased TfR1 edges connected through aggregate subnodes upon AX-53802 treatment, indicating TfR1 aggregate formation, likely a defensive response to ferroptosis induction that decreases labile iron content by slowing transferrin recycling. Another strength of the PLOM-CON method is its ability to handle post-translational modifications, achieved through staining with antibodies specific to these modifications, which are represented in the network as individual nodes. In the current study’s network, correlations involving phosphorylated FAK were significantly altered following AX-53802 treatment, whereas total FAK showed minimal change. These findings highlight PLOM-CON’s capacity to elucidate the complex dynamics of ferroptosis induction by capturing multifaceted protein-related information from microscopic images.

Building on the existing features of PLOM-CON analysis, this study introduces two enhancements aimed at improving drug MoA and combination drug target identification. First, we incorporated the correlation anomaly score, a quantitative metric, to address the challenge of visually comparing network structures and pinpointing key elements responsible for structural differences between drug-treated and untreated networks^52^. This metric facilitated the systematic identification of protein feature quantities most influential in the observed differences. Phosphorylated FAK emerged as one of the proteins with the highest correlation anomaly score, and its inhibition in combination with AX-53802 demonstrated a pronounced ferroptosis-promoting effect in a subset of cancer cell types (Figs. 6D and S18). Validation experiments suggested that FAK inhibition blocks the Nrf2 antioxidative stress response, possibly through reduced STAT3 phosphorylation and decreased Nrf2 expression (Fig. 6A). These results partially align with prior research showing that the α6β4 integrin-mediated signaling pathway protects cells from erastin-induced ferroptosis through Src and STAT3 activation^65^. The inhibition of this pathway using FAK/Src inhibitors reduced STAT3 activation, but contrary to prior reports, it did not increase ACSL4 expression in HEK293 cells, suggesting a cell type-dependent effect.

The second improvement entailed expanding the network to increase its comprehensiveness. Although PLOM-CON analysis is constrained by the number of nodes corresponding to immunostained proteins, limiting molecular information compared with gene networks such as DeMAND^1^, the present study included augmentation of the covariation network using comprehensive PPI network data. This process led to the identification of four additional candidates for combination drug therapy: MDM2, ezrin, cortactin, and Parkin. Validation experiments subsequently demonstrated that inhibitors targeting MDM2, ezrin, and cortactin deacetylation could effectively suppress ferroptosis, offering new avenues for mitigating various conditions, such as acute kidney injuries and neurodegenerative diseases, albeit requiring further validation in disease model cells. Although the precise MoA for each target remains to be elucidated, several inferences can be drawn from previous studies.

Ezrin, which links the cytoskeleton and cell membrane to regulate membrane tension, may play a crucial role in ferroptosis. Previous research suggests that membrane tension, heightened by lipid peroxide accumulation, activates mechanosensitive cation channels such as Piezo, resulting in non-selective cation flux and cell swelling, ultimately leading to membrane rupture^69^. In addition, Piezo-1 knockout suppressed ferroptosis, although lipid peroxidation levels remained elevated. Thus, the observed inhibitory effect of ezrin inhibition on ferroptosis could involve reducing membrane tension induced by lipid peroxide accumulation following GPX4 inhibition. This hypothesis is supported by the lack of an effect of ezrin inhibition on lipid peroxide generation itself, indicating control mechanisms downstream of lipid peroxidation. Future research using membrane tension probes, such as flipper-TR, is expected to yield significant insights.

The ferroptosis-inhibitory effect of nutlin-3a also appears to operate downstream of lipid peroxide generation, as evidenced in BODIPY-C11 experiments. Previous studies showed that nutlin-3a inhibits ferroptosis in MDAH041 cells treated with cystine uptake blockers^70^. Although MDM2 inhibition results in p53 activation, the protective effect was observed even in p53-deficient cells, suggesting a p53-independent mechanism^70^. A recent study has highlighted the p53-independent actions of MDM2, such as the regulation of focal adhesion formation and metastasis^71^, both of which may affect membrane tension and could therefore be associated with ferroptosis.

Other compounds exhibiting protective effects, such as CK666, EX527, and tubastatin A, may potentially reduce iron levels by inhibiting transferrin recycling, given their modulation of Arp2/3 and cortactin, which are associated with this process^72,73^. This hypothesis is supported by the observation that the inhibitors tend to suppress lipid peroxide generation, similar to iron chelators^74^. The relationship between cortactin acetylation and transferrin-mediated iron uptake warrants further investigation to ascertain the molecular mechanisms and therapeutic potential of these interactions.

Comparing our findings with those of Feng et al. ^43^, wherein TfR1 was identified as a specific ferroptosis marker, we observed interesting parallels and distinctions. Although AX-53802–induced changes elevated TfR1’s importance in our study, its accumulation pattern differed from that reported by Feng et al., with our system emphasizing intracellular aggregation over membrane accumulation. This discrepancy may be attributed to differences in the timing of ferroptosis induction observations: Feng et al. observed TfR1 accumulation at the cell membrane 2 h after RSL3 addition^43^, whereas we observed prominent aggregates 1 h after AX-53802 addition. This suggests that lipid peroxide production prompts rapid intracellular aggregation of TfR1 due to delayed recycling, potentially preceding its later cell surface localization. Considering the consistent decrease in free iron levels from 1 to 3 h after AX-53802 addition (Figs. 4F and S13), it is plausible that even if TfR1 accumulates on the cell surface, iron uptake via TfR1 may be compromised, particularly in HEK293 cells.

According to the classification of existing ferroptosis inducers, AX-53802 is classified as a Class II inhibitor, which covalently binds to GPX4^16,20,21^. This classification is supported by AX-53802’s structure, which includes a propiolamide group believed to form a Michael addition–type covalent bond^55^. WB analysis further demonstrated a band shift upon AX-53802 treatment, similar to other covalent inhibitors of GPX4^55,56^. In contrast to erastin-induced ferroptosis, which typically leads to synchronized cell death^33^, AX-53802 did not exhibit this behavior, consistent with GPX4 inhibition. Although these findings support the notion that AX-53802 is a GPX4 covalent binder, definitive confirmation of covalent binding necessitates detecting an increase in the molecular weight of the protein–inhibitor complex using mass spectrometry^29^. Additionally, experiments such as mutating Sec46 to inhibit binding would be required to verify AX-53802’s interaction with GPX4’s selenocysteine at position 46, akin to RSL3^19^.

Specificity poses a major challenge for Class II inhibitors^29^. RSL3 and ML162 are known to form covalent bonds with selenocysteine at position 46 in GPX4’s active site. However, the flat surrounding surface complicates achieving specificity through interactions with other regions. AX-53802 likely interacts with the active site in a similar manner, presenting analogous challenges. PLOM-CON analysis could aid in selecting drugs with the highest specificity, thereby mitigating unforeseen side effects. When comparing networks of multiple drugs with equivalent primary effects, drugs with lower specificity are likely to impact a broader range of network components compared with their more specific counterparts.

Comparing PLOM-CON with existing network-based methodologies that rely on GEP data, each has distinct strengths and weaknesses. DeMAND^1^, relying on GEP data, offers comprehensiveness but may not predict targets for certain drugs (e.g., blebbistatin targeting myosin II) or may predict pathways without identifying the exact target. For instance, studies employing the DeMAND algorithm have revealed that altretamine, with an unknown MoA, shares a high MoA similarity with sulfasalazine, an inhibitor of system x_c_^−^ (cystine/glutamate antiporter), implying involvement in the system x_c_^−^−GPX4 pathway^1^. However, subsequent experimental assays showed that the actual target was GPX4 rather than system x_c_^−^. In contrast, PLOM-CON, as a protein-level network analysis method, is more likely to distinguish the specific effects of system x_c_^−^ and GPX4 inhibitors at the protein level. Despite this strength, a notable limitation of PLOM-CON is its reliance on antibodies to label individual targets, which restricts its detection capacity to only a few dozen proteins, thereby limiting the comprehensiveness of its analysis results. This dependency may also reduce its applicability, especially when targeting less common proteins with limited availability of commercial antibodies targeting them. When the relevant pathways are unknown, the identification of potential pathways is necessary to enable the selection of appropriate antibodies for the PLOM-CON method. In such cases, comprehensive gene variation analyses, such as DNA microarrays or RNA-seq, followed by GO enrichment or pathway analysis using databases, such as the KEGG pathway or WikiPathways, can be performed. Furthermore, combining MoA estimation methods using GEPs, such as DeMAND, with PLOM-CON’s protein-level analysis could effectively elucidate the detailed MoA of unknown compounds.

In further research, the same set of antibodies used in the present study can be employed to investigate ferroptosis in various contexts. Moreover, considering that ferroptosis involves not only proteins but also lipids, such as polyunsaturated fatty acids and ions, various probes (e.g., iron probes, Ca^2+^ ion probes, lipid peroxide probes, and membrane tension probes) could enable the construction of a more comprehensive covariation network. Expanding PLOM-CON methodologies to incorporate multimodal data spanning proteins, genes, lipids, ions, and biophysical parameters is expected to enhance our understanding of ferroptosis.

## Methods

### Screening for ferroptosis inducers

Cells were seeded at 500 cells/well in 384-well plates (Corning, NY, USA). Following overnight incubation, 3 μM of each test compound from >50,000 in a structurally diverse library was added to cells with either 1 μM Fer1 or DMSO. After 72 h, cell viability was assessed using the CellTiter-Glo Luminescent Cell Viability Assay (Promega, Madison, WI, USA) following the manufacturer’s instructions.

### Cell culture and reagents

HEK293 cells and HeLa cells were obtained from an existing collection at the Kano Laboratory, Tokyo Institute of Technology. SW480 and HT29 cells were purchased from the American Type Culture Collection (Manassas, VA, USA), HCT-116 cells were obtained from the European Collection of Authenticated Cell Cultures (Porton Down, Salisbury, UK), and HT-1080 cells were acquired from the JCRB. HEK293 cells were cultured in Dulbecco’s modified essential medium (DMEM; #041-29775; Fujifilm) supplemented with 10% fetal bovine serum (FBS; Sigma-Aldrich, St. Louis, MO, USA), 100 U/mL penicillin, and 100 μg/mL streptomycin (#15140-122; Gibco) in 5% CO_2_ at 37 °C. HEK293 cells stably expressing GFP-GPX4 were maintained in the same medium with 500 μg/mL Geneticin (#11811-031; Gibco). HeLa, SW480, HT29, and HCT-116 cells were cultured in DMEM (#05915; Nissui Pharmaceutical, Tokyo, Japan) supplemented with 10% FBS, 100 U/mL penicillin, and 100 μg/mL streptomycin in 5% CO_2_ at 37 °C. HT-1080 cells were cultured in Eagle’s minimum essential medium (#051-07615; Fujifilm) supplemented with 10% FBS, 1% non-essential amino acids (#139-15651; Fujifilm), 100 U/mL penicillin, and 100 μg/mL streptomycin in 5% CO_2_ at 37 °C. Details of reagents used in this study are provided in Supplementary Data 3.

### Cell death manner assay

HEK293 cells were seeded at a density of 10,000 cells/well in 96-well plates and cultured at 37 °C overnight. Cells were then cotreated with AX-53802 and several cell death inhibitors for 24 h: Fer1 (1 μM), tocopherol (100 μM), deferoxamine (20 μM), z-vad-fmk (20 μM), and Nec-1s (10 μM). Viability was assessed using the CellTiter-Glo 2.0 Cell Viability Assay (#G9242; Promega). Cell death assay was conducted by staining dead cells with 3.3 μg/ml propidium iodide (PI) for 1 h.

### Determination of lipid peroxides

HEK293 cells were seeded at a density of 10,000 cells/well in 96-well plates and cultured at 37 °C overnight. Cells were then prestained with 30 μM BODIPY 581/591 C11 (#D3861; Invitrogen) for 1 h, followed by treatment with each drug for 1–2 h. Observations were made using a Nikon A1 confocal laser-scanning microscope (Nikon, Tokyo, Japan) with a ×40 objective.

### Immunofluorescence and microscopy

Cells in 96-well plates were fixed in 4% paraformaldehyde for 20 min, permeabilized using 0.2% Triton X-100 for 15 min, and blocked with 3% bovine serum albumin for 30 min. Primary antibodies were then applied in blocking buffer overnight at 4 °C, followed by incubation with fluorophore-conjugated secondary antibodies and Hoechst 33342 (DOJINDO) in blocking buffer at room temperature for 1 h. Imaging was conducted using a Nikon A1 confocal laser-scanning microscope with a ×40 objective (Plan Apo λ 40× NA0.95; Nikon).

For PLOM-CON analysis, HEK293 cells were seeded at a density of 10,000 cells per well in 96-well plates (#655090; Greiner) and cultured overnight at 37 °C. Cells were then treated with each drug for durations of 5, 10, 15, 20, 25, 30, 40, 50, and 60 min, fixed in 4% paraformaldehyde for 20 min, and processed for immunofluorescence staining as previously described. All cells were stained with anti-GAPDH antibody as a cytoplasm marker and Hoechst 33342 as a nuclear marker.

The primary antibodies used for immunofluorescence were as follows: SLC7A11 (Invitrogen; #PA5-116134; 1:200), SLC3A2 (Sigma; #HPA017980; 1:500), GPx4 (abcam; #ab125066; 1:100), ATF4 (CST; #11815S; 1:100), HSPA5 (BiP, GRP78) (abcam; #ab21685; 1:800), Nrf2 (MBL; #M200-3; 1:1000), AIFM2 (FSP1) (Sigma; #HPA02896; 1:50), DHODH (Sigma; #HPA010123; 1:250), FATP2 (SLC27A2) (Invitrogen; #PA5-102343; 1:800), TfR1 (Invitrogen; #13-6800; 1:250), HSPB1 (e Bioscience; #14-9112-80; 1:50), FT (Invitrogen; #MA5-14736; 1:100), HIF1a (abcam; #ab179483; 1:100), NCOA4 (Sigma; #HPA051260; 1:200), ATG7 (abcam; #ab201251; 1:200), HSP90α (Invitrogen; #MA5-25036; 1:200), KEAP1 (CST; #8047; 1:400), HO-1 (abcam; #ab214643; 1:100), NF2 (merlin) (Sigma; #HPA003097; 1:400), LATS1 (MyBioSource; #MBS9610532; 1:50), YAP1 (CST; #14729; 1:400), LSH (HELLS) (Sigma; #HPA063242; 1:200), EGLN1 (Invitrogen; #PA5-78511; 1:800), 4HNE (JalCA; #MHN-020P; 1:50), ACSL4 (Invitrogen; #PA5-100033; 1:400), F-actin (Phalloidin) (Invitrogen; #A22283; 1:200), Myosin IIB (CST; #8824; 1:200), Beta tubulin (SIGMA; #T8328; 1:500), pFAK (abcam; #ab81298; 1:400), FAK (Invitrogen; #39-6500; 1:250), LAMP2 (Hybridoma Bank; #H4B4-c; 1:200), mTOR (CST; #2983; 1:400), pAKT (Merk; #05-1003; 1:500), pS6rp (CST; #4858; 1:100), TFEB (CST; #4240; 1:100), GAPDH (abcam; #ab83956; 1:200). The secondary antibodies used were: Alexa-488-anti-Mouse IgG (molecular probes; Cat#A11029; 1:200), Alexa-546-anti-Mouse IgG (molecular probes; Cat#A11030; 1:200), Cy5-anti-Chicken IgY, (abcam; Cat#ab97147; 1:200), Alexa Fluor 488-anti-Rabbit IgG (abcam; Cat#ab150061; 1:200), Alexa Fluor 647-anti-Mouse IgG (abcam; Cat#ab150111; 1:200).

Automated image acquisition was performed using NIS-Elements Ver 5.40 software (Nikon) to capture Z-stack images from +10 μm to −6 μm relative to the focal point, with 0.65 μm increments per step, for over 1000 cells per well. Hoechst 33342 was used for autofocusing, and gain and laser power was adjusted for each protein to avoid exceeding the upper fluorescence intensity limit. Image acquisition for PLOM-CON analysis was performed by Nikon Corp.

### Image analysis and visualization

For analyses excluding PLOM-CON, microscopic images were analyzed using NIS-Elements Ver 5.3 (Nikon, RRID:SCR_014329) software. Maximum intensity projection of Z-stack images was performed for further analysis. Cell segmentation involved initial nucleus detection from Hoechst-stained images, followed by determination of cell area using the watershed algorithm with GAPDH or CellMask-stained images. The cytoplasmic region was defined by subtracting the nucleus region from the total cell area. Fluorescence intensities were measured per region, and data were passed to R version 4.2.1 with RStudio (version 2022.07.1 + 554; RStudio Inc., MA, USA) for further analysis and visualization.

For PLOM-CON analysis, image analysis was conducted using the “signaling pathway analysis service” provided by Nikon Corp., employing NIS-Elements Ver 5.4 (Nikon, RRID:SCR_014329) software. Maximum intensity projection and segmentation were performed as described earlier with minor modifications. Antibodies showing localized bright staining regions were detected using specific algorithms (e.g., the Detect Regional Maxima algorithm) and categorized into relatively large “domains,” small “aggregates,” and PM-associated aggregates. Supplementary Data 2 lists all feature quantities, including fluorescence intensity across cellular compartments, rates of domain/aggregate formation, and morphological characteristics, such as area and circularity.

### Covariation network inference

Estimation of the covariation network using single-cell data of feature quantities was conducted by Nikon Corp. using the “signaling pathway analysis service,” as previously described^10^. An overview of this method is illustrated in Fig. S5. A comparison of network inference methods is provided in Supplementary Discussion.

### Correlation anomaly scores

Correlation anomaly scores, as proposed by Ide et al.^52^, were used to quantify differences between two covariation networks derived from feature quantities under different drug treatment conditions. Consider two datasets corresponding to feature quantities obtained from two conditions of drug treatment as follows:

$${{{{\mathcal{D}}}}}_{{{{\rm{A}}}}}\equiv \left\{{x}_{{{{\rm{A}}}}}^{\left(n\right)}\left|{x}_{{{{\rm{A}}}}}^{\left(n\right)}\right.\in {{\mathbb{R}}}^{M},n=1,2,\ldots ,{N}_{{{{\rm{A}}}}}\right\}$$

$${{{{\mathcal{D}}}}}_{{{{\rm{B}}}}}\equiv \left\{{x}_{{{{\rm{B}}}}}^{\left(n\right)}\left|{x}_{{{{\rm{B}}}}}^{\left(n\right)}\right.\in {{\mathbb{R}}}^{M},n=1,2,\ldots ,{N}_{{{{\rm{B}}}}}\right\}$$

The expected value of the Kullback–Leibler divergence^75^ for the *i*th node (protein and morphology information) is as follows:

$${d}_{i}^{{{{\rm{AB}}}}}\equiv \int {{{\rm{d}}}}{z}_{i}{p}_{{{{\rm{A}}}}}\left({z}_{i}\right)\int {{{\rm{d}}}}\,{x}_{i}{p}_{{{{\rm{A}}}}}\left({x}_{i}|{z}_{i}\right){{\mathrm{ln}}}\tfrac{{p}_{{{{\rm{A}}}}}\left({x}_{i}\left|{z}_{i}\right.\right)}{{p}_{{{{\rm{B}}}}}\left({x}_{i}\left|{z}_{i}\right.\right)}$$

$${z}_{i}\equiv {\left({x}_{1}^{\left(n\right)},\ldots ,{x}_{i-1}^{\left(n\right)},{x}_{i+1}^{\left(n\right)},\ldots ,{x}_{M}^{\left(n\right)}\right)}^{{{\top }}}\in {{\mathbb{R}}}^{M-1}$$

Calculating this integral yields the following:

$${d}_{i}^{{{{\rm{AB}}}}}=\, 	 {w}_{{{{\rm{A}}}}}^{{{\top }}}\left({l}_{{{{\rm{B}}}}}-{l}_{{{{\rm{A}}}}}\right)+\frac{1}{2}\left\{\frac{{l}_{{{{\rm{B}}}}}^{{{\top }}}{{{{\rm{W}}}}}_{{{{\rm{A}}}}}{l}_{{{{\rm{B}}}}}}{{\lambda }_{{{{\rm{B}}}}}}-\frac{{l}_{{{{\rm{A}}}}}^{{{\top }}}{{{{\rm{W}}}}}_{{{{\rm{A}}}}}{l}_{{{{\rm{A}}}}}}{{\lambda }_{{{{\rm{A}}}}}}\right\}\\ 	 +\frac{1}{2}\left\{{{\mathrm{ln}}}\frac{{\lambda }_{{{{\rm{A}}}}}}{{\lambda }_{{{{\rm{B}}}}}}+{\sigma }_{{{{\rm{A}}}}}\left({\lambda }_{{{{\rm{B}}}}}-{\lambda }_{{{{\rm{A}}}}}\right)\right\}$$

$${{{\Lambda }}}_{{{{\rm{A}}}}}=\left(\begin{array}{cc}{L}_{A} & {l}_{A}\\ {l}_{A}^{{{\top }}} & {\lambda }_{A}\end{array}\right),{{{\Sigma }}}_{{{{\rm{A}}}}}\equiv {{{\Lambda }}}_{{{{\rm{A}}}}}^{-1}=\left(\begin{array}{cc}{W}_{{{{\rm{A}}}}} & {w}_{{{{\rm{A}}}}}\\ {w}_{{{{\rm{A}}}}}^{{{\top }}} & {\sigma }_{{{{\rm{A}}}}}\end{array}\right)$$

The correlation anomaly score ${a}_{i}$ is defined as the maximum value of ${d}_{i}^{{{{\rm{AB}}}}}$ and ${d}_{i}^{{{{\rm{BA}}}}}$.

$${a}_{i}=\max \left\{{d}_{i}^{{{{\rm{AB}}}}},{d}_{i}^{{{{\rm{BA}}}}}\right\}$$

### Graph clustering and network expansion using a PPI database

Graph clustering and network expansion were performed to identify a broader spectrum of target molecules for combination therapy. Graph clustering followed previously described methods^10,58^. Briefly, the covariation network was regarded as an undirected graph, G = (V, E) with *n* vertexes (nodes) and *m* edges. The Overlapping Cluster Generator^58^ was then employed to decompose the network into overlapping subnetworks, using the R package linkcomm^76^. Clusters with a mean degree 2|E_i|/|V_i| exceeding the mean degree of the whole graph were selected for each L1 regularization parameter. Subsequently, clusters were expanded using common PPI data from the BioGrid database^59^, targeting the first neighbors of the cluster components. Only proteins sharing common GO terms enriched in the extracted clusters were selected for expansion.

### Validation and selection of antibodies

HEK293 cells plated in 96-well plates were left untreated or treated without AX-53802 for 1 h, followed by fixation, permeabilization, and immunofluorescence processing, as described earlier. Staining patterns were validated against various references, including the Human Protein Atlas and antibody manufacturers’ data. Antibodies were selected based on observable changes in response to AX-53802, resulting in a curated list of 35 antibodies detailed in Supplementary Data 1. As immunofluorescence largely depends on the accessibility of antibodies to protein epitopes, observed signals may not quantitatively represent protein levels, as they can be influenced by protein localization, conformational changes, and interactions with other molecules. Therefore, immunofluorescence images potentially offer a more sensitive indicator of protein intracellular states compared to the quantitative data from biochemical experiments.

### In vitro GPX4 enzyme activity assay

The direct inhibitory effect of AX-53802 on GPX4 was assessed using the GPX4 Inhibitor Screening Assay Kit (#701880; Cayman Chemical) following the manufacturer’s instructions. Briefly, test compounds dissolved in GPX4 assay buffer were incubated with GPX4 enzyme at room temperature for 1 h. A mixture of glutathione, glutathione reductase, reconstituted NADPH, and cumene hydroperoxide was then added and mixed. Subsequently, kinetic detection was performed using a microplate reader to measure absorbance at 340 nm for 5 min in 1 min intervals.

### Western blotting assay

Cells cultured in 6-well plates were lysed with 100 μL of ice-cold radioimmunoprecipitation assay buffer [1% Triton X-100, 0.1% sodium dodecyl sulfate, 1% sodium deoxycholate, 150 mM NaCl, and 50 mM Tris-HCl (pH 8.0)] supplemented with protease inhibitor mixture (#11697498001; Roche) and PhosSTOP phosphatase inhibitor mixture (#4906837001; Roche). Cell lysates were homogenized via passage through a 27-gauge needle 10–15 times, mixed with an equal volume of 2× Laemmli’s sodium dodecyl sulfate sample buffer, boiled for 5 min, and subjected to 12% or a 5%–20% gradient sodium dodecyl sulfate-polyacrylamide gel electrophoresis. Subsequently, they were transferred to a polyvinylidene fluoride membrane (IPVH00010; Merck Millipore) using a Trans-Blot SD cell (Bio-Rad) at 70 V for 120–180 min. The membrane was blocked with 5% (w/v) bovine serum albumin, probed with primary antibodies overnight at 4 °C, and incubated with horseradish peroxidase-conjugated secondary antibodies at room temperature for 1 h. Finally, the proteins were detected using the Western Lightning Plus-ECL system (PerkinElmer) and a LAS 4000 mini imager (Fujifilm).

The primary antibodies used for western blotting were as follows: GPx4 (abcam; #ab125066; 1:1000), pFAK (abcam; #ab81298; 1:1000), FAK (Invitrogen; #39-6500; 1:500), pSTAT3(Y705) (CST; #Cat#9145; 1:2000), STAT3 (CST; #Cat#9139; 1:1000), Nrf2 (MBL; #M200-3; 1:1000), ACSL4 (Invitrogen; #PA5-100033; 1:1000), GAPDH (for WB) (Millipore; #Cat#MAB374; 1:4000). The secondary antibodies used were: Anti-rabbit-HRP (CST; #7074; 1:2000), Anti-Mouse-HRP (Promega; #W4021; 1:2000).

### Generation of cells stably expressing GFP-GPX4

A plasmid expressing GFP-tagged human GPX4 was constructed by inserting human GPX4 cDNA, including the 3′ untranslated region containing the selenocysteine insertion sequence (GenBank accession no. NM_002085.5; Cytoplasmic isoform, Uniprot ID: P36969-2), into the pEGFP-C vector (Clontech, Mountain View, CA, USA) using the In-Fusion HD Cloning Kit (Takara, Shiga, Japan). HEK293 cells were cultured in 6-well plates until semi-confluent and then transfected with the plasmid using FuGENE HD Transfection Reagent (#E2311; Promega; WI, USA), following the manufacturer’s instructions. Post-transfection, cells were passaged several times in media containing 1000 μg/mL Geneticin for selection.

### Intracellular iron assay

Intracellular ferrous ion levels were assessed using FerroOrange dye (#GC904-02; Goryo Chemical), according to the manufacturer’s instructions. Cells were treated with each drug for the indicated time, followed by incubation for 30 min with 100 μM FerroOrange diluted in serum-free medium. Imaging was performed using a Nikon A1 confocal laser-scanning microscope with a ×40 objective.

### Total RNA extraction and RT-PCR

Cells cultured in 6-well plates were treated with each drug. Total RNA was then purified using the RNeasy Mini Kit (Qiagen) and reverse-transcribed using a ReverTra Ace qPCR RT Kit (Toyobo). Real-time PCR was conducted using fast SYBR Green Master Mix (Applied Biosystems, Foster City, CA, USA) and the StepOnePlus Real-Time PCR System (Applied Biosystems). The primer oligonucleotides used for real-time PCR are as follows: forward, 5′-CACATCCAGTCAGAAACCAGTGG-3′ and reverse 5′-GAATGTCTGCGCCAAAAGCTG-3′ for *NRF2*; forward, 5′-CCAGGCAGAGAATGCTGAGTTC-3′ and reverse 5′-AAGACTGGGCTCTCCTTGTTGC-3′ for *HMOX1*; forward, 5′-TCCTGCTTTGGCTCCATGAACG-3′ and reverse 5′-AGAGGAGTGTGCTTGCGGACAT-3′ for *SLC7A11*; forward, 5′-GTTCAGTCCTTGGAGTTGCACA-3′ and reverse 5′-CCCAGTAAGGCTGTAAATGCTC-3′ for *GCLM*; forward, 5′-CATCCCTGGAGCAGATACTCT-3′ and reverse 5′-TCACTTAGGATTTCCCTGGTCC-3′ for *ACSL4*; forward, 5′-TCATCAGCAATGCCTCCTG-3′ and reverse 5′-GGCCATCCACAGTCTTCTG-3′ for *GAPDH*. *GAPDH* served as the internal standard.

### DPPH antioxidant assay

Free radical scavenging activity of the test compounds was assessed using the DPPH Antioxidant Assay Kit (#347-09561; Fujifilm) following the manufacturer’s instructions. Briefly, DPPH was dissolved in ethanol and sonicated to prepare the DPPH working solution. Test compounds were mixed with assay buffer and DPPH working solution, followed by incubation at 25 °C for 30 min in the dark. Absorbance was measured at 517 nm using a microplate reader, and the percentage inhibition of DPPH was calculated.

### Statistics and reproducibility

Statistical significance of observed differences was determined using Dunnett’s test (two-sided) for comparisons against the control group, with Tukey’s test (two-sided) used for pairwise comparisons among all groups. Results are presented as means ± standard errors of the mean unless otherwise specified. The number of samples analyzed is shown in each figure legend. *P* < 0.05 was considered statistically significant. For single-cell analysis, effect size was calculated between conditions using Cliff’s Delta. All statistical analyses were performed using R version 4.2.1.

### Reporting summary

Further information on research design is available in the Nature Portfolio Reporting Summary linked to this article.

## Supplementary information

Supplementary Information

Description of Additional Supplementary Files

Supplementary Data 1

Supplementary Data 2

Supplementary Data 3

Supplementary Data 4

Supplementary Video S1

Supplementary Video S2

Supplementary Video S3

Supplementary Video S4

Supplementary Video S5

Supplementary Video S6

Reporting Summary

## Data Availability

The input and output files for the PLOM-CON analysis, correlation anomaly analysis, graph clustering, and network expansion are available in Mendeley Data (10.17632/x3bnb4fhw4.3)^77^. Supplementary Data 4 contains the source data used for the graphs presented in the main figures. Supplementary Fig. S19 includes the uncropped and unedited blot images. Additional data files that support this study are available upon request.

## Code availability

The codes for graph clustering and network expansion are provided in Mendeley data (10.17632/x3bnb4fhw4.3)^77^. The code with a toy dataset for the PLOM-CON analysis was previously published^8^ and is available via Mendeley data at: 10.17632/vzz7xpymxd.1^78^.
