# Supplementary material for: Protein covariation networks for elucidating ferroptosis inducer mechanisms and potential synergistic drug targets
Source: Commun Biol. 2025 Mar 31;8:480. doi: 10.1038/s42003-025-07886-3 (PMC11958834; doi:10.1038/s42003-025-07886-3)
Supplement: Supplementary file 1 — Supplementary Information [file 42003_2025_7886_MOESM1_ESM.pdf]

Supplementary Information

Protein covariation networks for elucidating ferroptosis inducer mechanisms and potential synergistic drug targets

Rina Kunishige[1, 2], Yoshiyuki Noguchi[2, 3], Naomi Okamoto[2], Lei Li[2], Akito Ono[4], Masayuki Murata[1, 2], Fumi Kano[1, 2, 5 *]

Affiliations

1. Multimodal Cell Analysis Collaborative Research Cluster, Institute of Science Tokyo, Yokohama-shi, Kanagawa, 226-8503, Japan
2. Cellshoot Therapeutics, Inc., Koto-ku, Tokyo, 136-0082, Japan
3. International Research Center for Neurointelligence, Institutes for Advanced Study, The University of Tokyo
4. Axcelead Drug Discovery Partners, Inc., Fujisawa, Kanagawa 251-8555, Japan.
5. Cell Biology Center, Institute of Integrated Research, Institute of Science Tokyo, Yokohama-shi, Kanagawa, 226-8503, Japan

* Corresponding author email: kanou.f.f7f1@m.isct.ac.jp / kano.f.aa@m.titech.ac.jp

Supplementary Figures

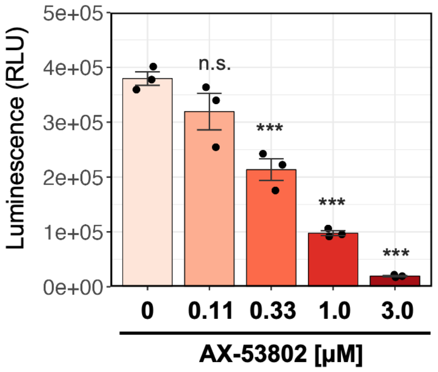

Fig. S1 Viability of HT-1080 cells treated with various AX-53802 concentrations.

HT-1080 cells were treated with the indicated AX-53802 concentrations for 24 h and measured via a CellTiter-Glo assay. Data: means ± SEMs (n = 3). ***P < 0.001; ns: not significant (Dunnett's test).

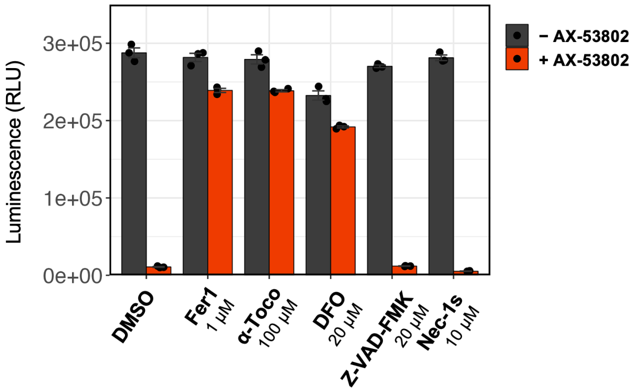

Fig. S2 Inhibition of AX-53802–induced cell death by lipophilic antioxidants and an iron chelator.

Viability measurements of HEK293 cells treated with AX-53802 and various cell death inhibitors, assessed via the CellTiter-Glo assay. Cotreatment with lipophilic antioxidants Fer1 (1 μM) or tocopherol (α-Toco; 100 μM), or with the iron chelator deferoxamine (DFO; 20 μM), inhibited cell death, whereas inhibitors of apoptosis (z-vad-fmk; 20 μM) and necrosis (Nec-1s; 10 μM) did not. A slight reduction in proliferation (<20%) was observed with single administration of 20 μM DFO. Data: means ± SEMs (n = 3).

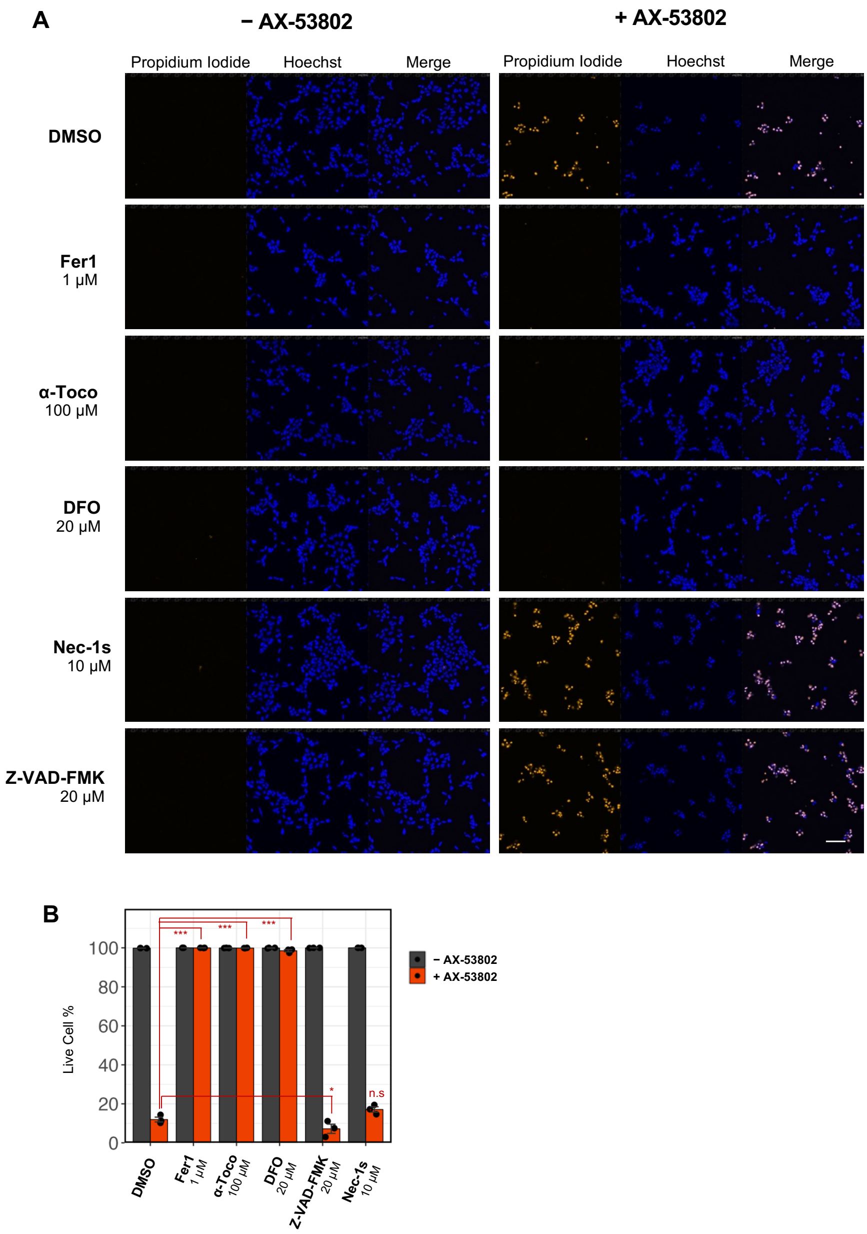

Fig. S3 Assay of HEK293 cell death after treatment with AX-53802 and cell death inhibitors. (**A**) Cell death detection via PI staining. Cotreatment with the lipophilic antioxidants Fer1 (1 μM) or tocopherol (α-Toco; 100 μM) or with the iron chelator deferoxamine (DFO; 20 μM) inhibits cell death, whereas apoptosis (z-vad-fmk; 20 μM) and necrosis (Nec-1s; 10 μM) inhibitors do not. Scale bar: 100 μm.

(B) Quantification of the PI-stained samples shown in (A). The percentage of live cells (PI-negative) compared to that of the total number of cells (Hoechst-stained) was calculated. Data are shown as means ± SEMs (n = 3). *P < 0.05; ***P < 0.001; ns: not significant (Dunnett's test).

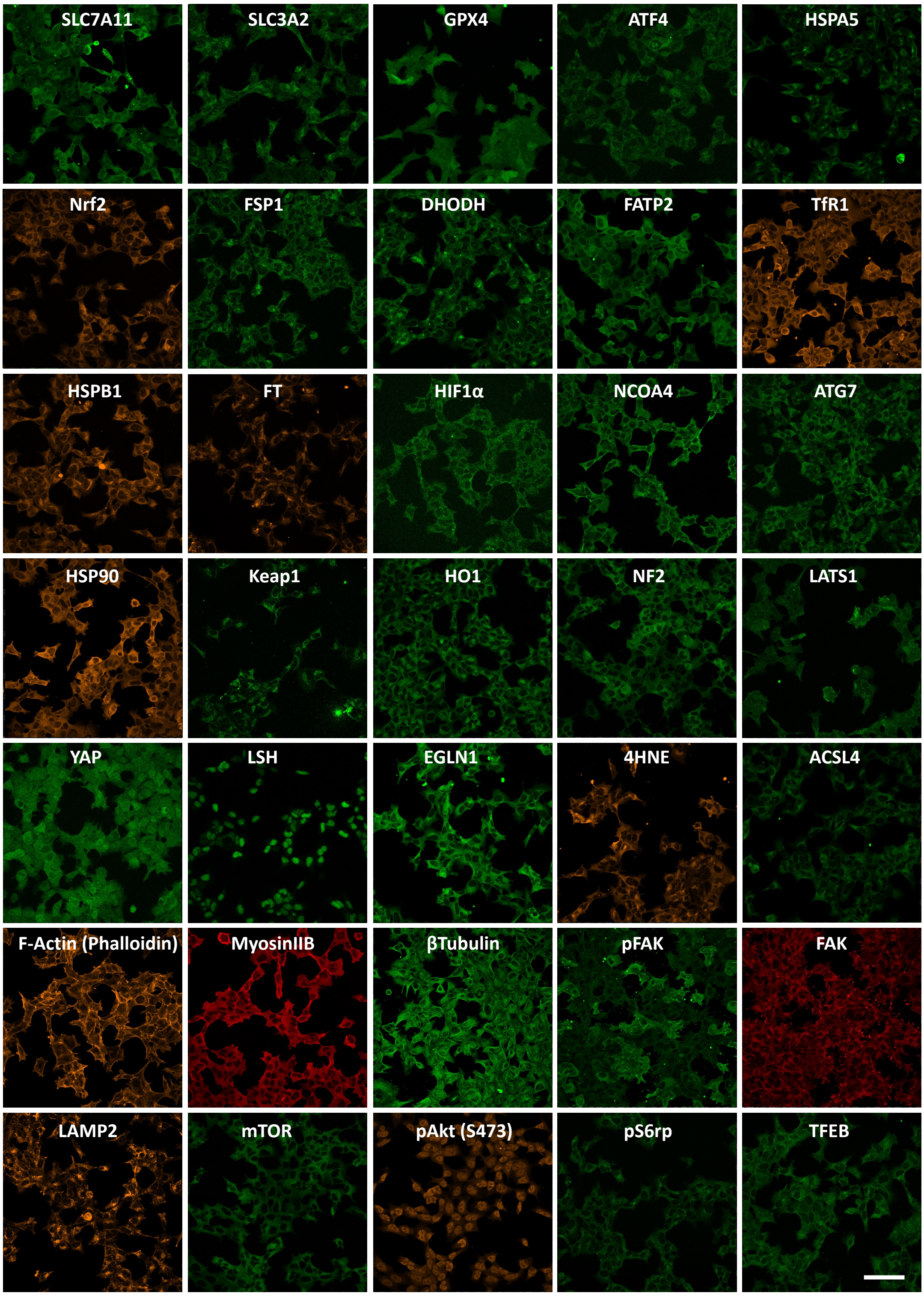

Fig. S4 Immunofluorescence staining of HEK293 cells with 35 antibodies used for PLOM-CON analysis.

HEK293 cells were fixed, permeabilized, and stained using the indicated antibodies. Images were obtained using a confocal laser-scanning microscope (×40 objective). Scale bar: 100 μm.

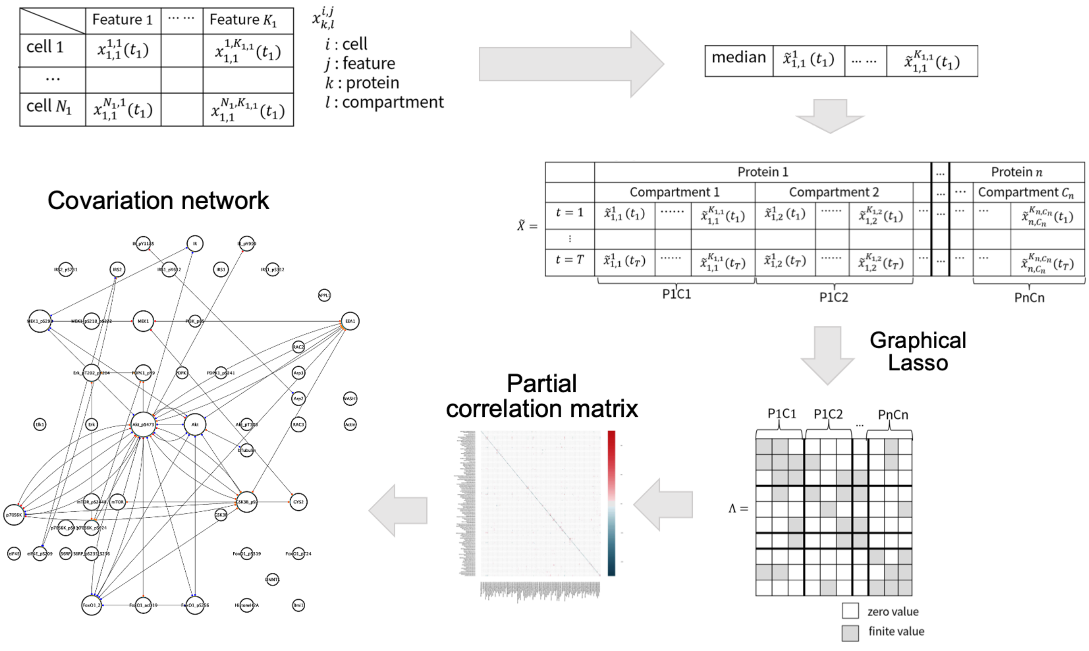

Fig. S5 Estimation of covariation network [adapted from Noguchi et al. (2021), iScience, 24(7), p. 4].

Single-cell data of feature quantities was obtained as described in Fig. 2B. Covariation network was estimated as previously described (Noguchi et al., 2021)[1]. Median values of cells for each condition and time point were calculated and merged into a comprehensive matrix incorporating all features, localizations (compartments), and proteins at each time point. A covariance matrix representing the correlation of time changes between each feature quantity was then calculated. Graphical lasso was applied to derive a sparse precision matrix, where zero elements indicate that corresponding variables are conditionally independent. A partial correlation matrix was obtained by normalizing the precision matrix with diagonal components, followed by selection of the maximum value for each protein localization unit. The resulting matrix was visualized as a covariation network, with proteins as nodes and color-coded subnodes representing localizations.

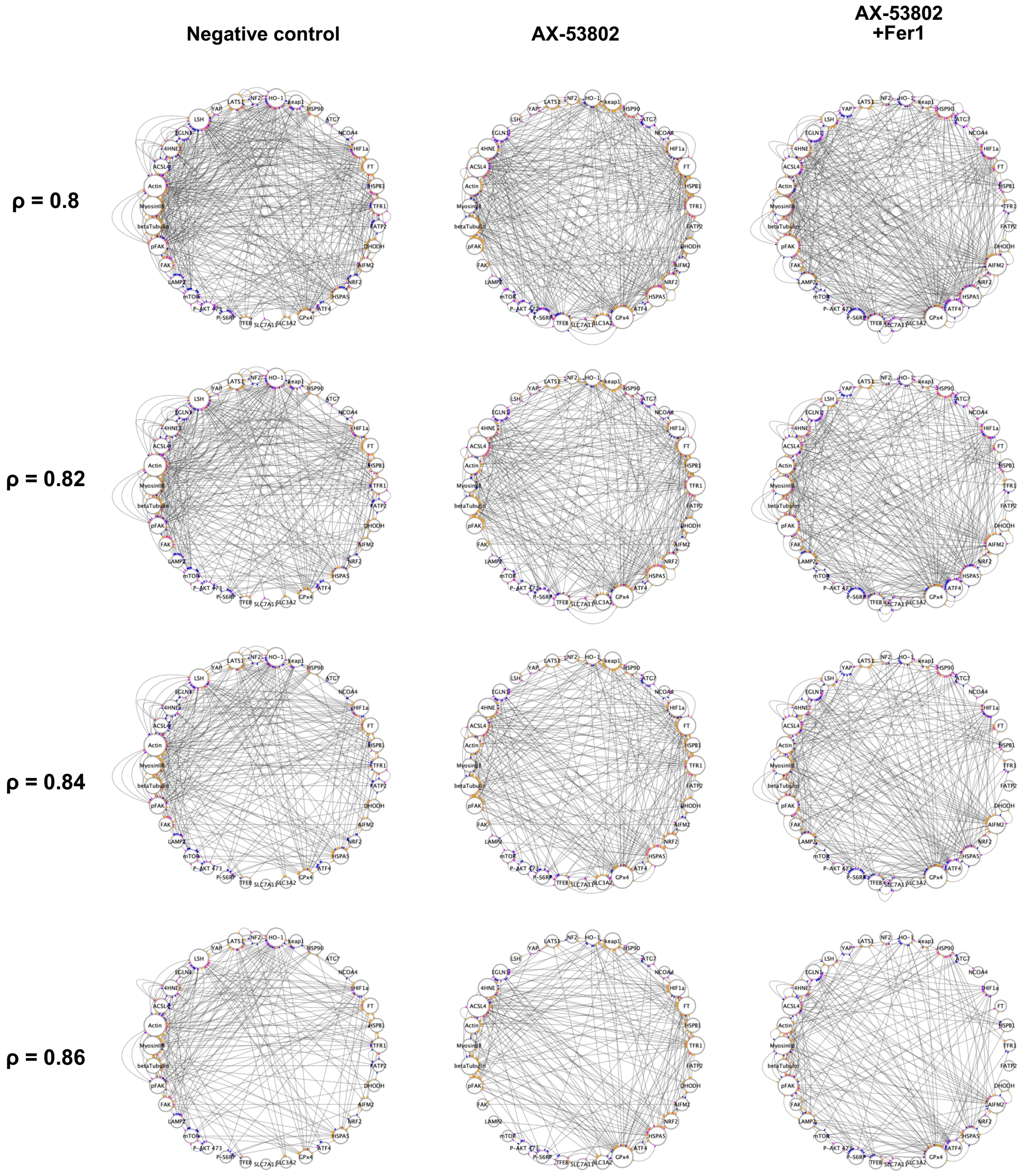

Fig. S6 Covariation networks for three treatment conditions at varying ρ values (ρ = 0.8 to 0.86).

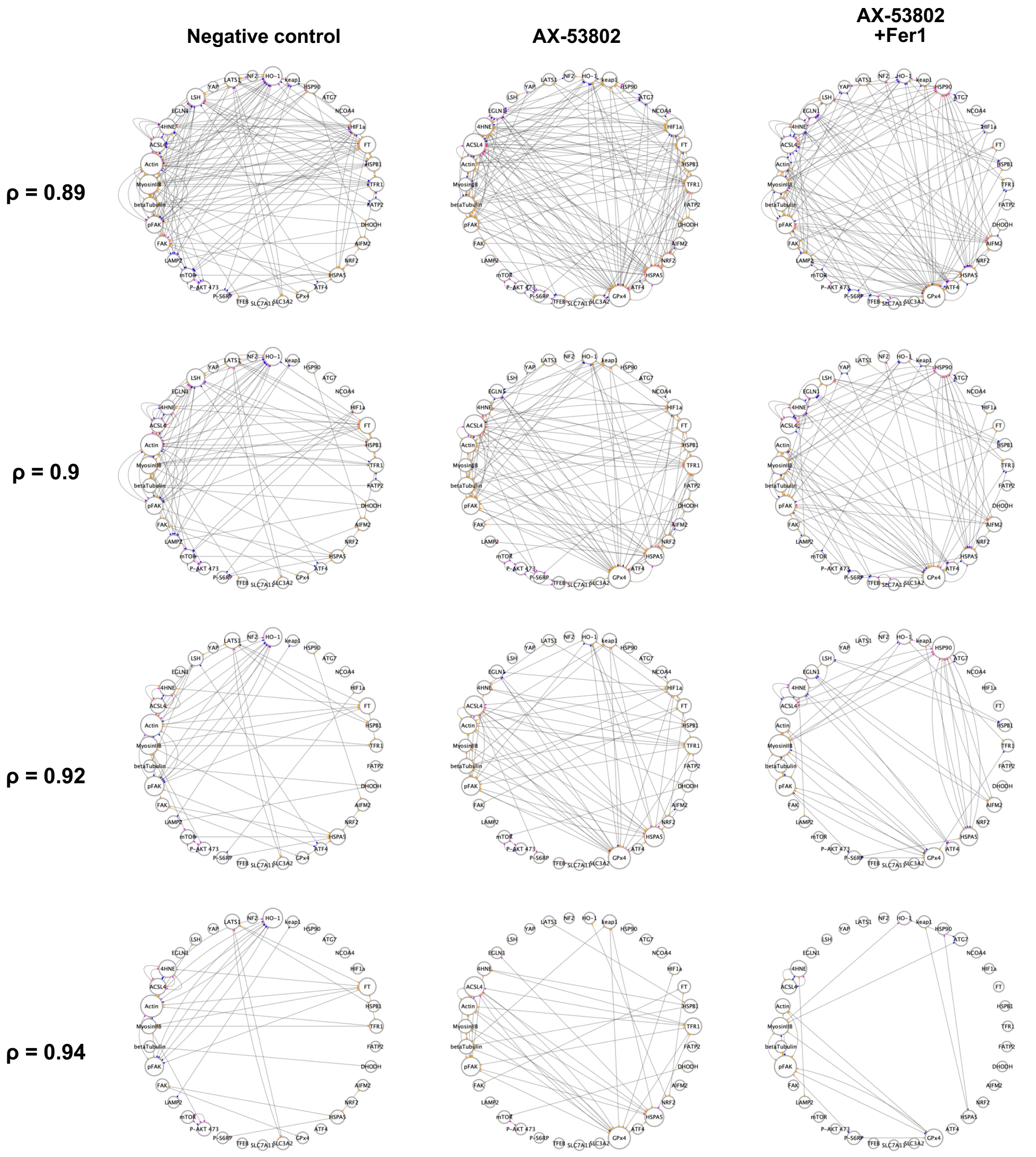

Fig. S6 Covariation networks for three treatment conditions at varying ρ values (ρ = 0.89 to 0.94).

In the graphical lasso method, the regularization parameter ρ controls network sparsity, with higher ρ values resulting in fewer edges. An edge between nodes indicates a temporal correlation between protein feature quantities within each localization. Subnodes are color-coded by the cellular localizations of feature quantities; blue, nucleus; pink, cytoplasm; orange, domains and aggregates.

ρ = 0.92 Negative control

Node 1	10	15	20	25	30	40	50	60 (min)	10	15	20	25	30	40	50	60 (min)	Node 2
TFR1[Localization>_Aggr]SumCount	0.80	0.80	0.80	0.80	0.80	0.80	0.80	0.80	2.00	2.00	2.00	2.00	2.00	2.00	2.00	2.00	FT[Localization>_Domain]SumCount
TFR1[Localization>_Aggr]SumCount	0.80	0.80	0.80	0.80	0.80	0.80	0.80	0.80	0.67	0.67	0.67	0.67	0.67	0.67	0.67	0.67	Actin[Localization>_Aggr]SumCount
TFR1[Localization>_Aggr]SumCount	0.80	0.80	0.80	0.80	0.80	0.80	0.80	0.80	1.17	1.17	1.17	1.17	1.17	1.17	1.17	1.17	MyosinIIB[Localization>_PM]SumCount
HSPB1[Localization>_Domain]AreaFraction	0.81	0.79	0.86	0.87	0.83	0.83	0.84	0.85	1.13	1.14	1.09	1.08	1.10	1.11	1.13	1.09	LATS1[Localization>_Aggr]Mint
FT[Localization>_AggrGrp]AreaFraction	1.30	0.94	0.99	1.00	0.99	0.95	0.95	0.93	2.00	1.00	1.00	1.00	1.00	1.00	1.00	1.00	LATS1[Localization>_Aggr]SumCount
FT[Localization>_Aggr]Mint	0.95	1.04	1.01	1.09	1.12	1.05	1.08	0.99	0.95	1.05	0.95	1.13	1.15	1.11	1.12	0.99	4HNE[Localization>_Domain]AreaFraction
FT[Localization>_Domain]SumCount	2.00	2.00	2.00	2.00	2.00	2.00	2.00	2.00	0.67	0.67	0.67	0.67	0.67	0.67	0.67	0.67	Actin[Localization>_Aggr]SumCount
FT[Localization>_Domain]SumCount	2.00	2.00	2.00	2.00	2.00	2.00	2.00	2.00	1.17	1.17	1.17	1.17	1.17	1.17	1.17	1.17	MyosinIIB[Localization>_PM]SumCount
keap1[Nuc>_Region]Mint	1.02	0.86	0.94	0.93	1.01	0.88	0.91	0.82	0.98	0.85	0.88	0.88	0.96	0.88	0.87	0.80	LAMP2[Nuc>_Region]Mint
HO-1[Nuc>_Region]Mint	0.86	0.75	0.74	0.68	0.68	0.73	0.67	0.68	0.88	0.75	0.72	0.69	0.70	0.71	0.64	0.61	Actin[Cyto>_ExLo]Mint
HO-1[Nuc>_Region]Mint	0.86	0.75	0.74	0.68	0.68	0.73	0.67	0.68	0.81	0.65	0.62	0.63	0.58	0.73	0.56	0.54	Actin[Localization>_Aggr]SumArea
HO-1[Nuc>_Region]Mint	0.86	0.75	0.74	0.68	0.68	0.73	0.67	0.68	0.91	0.72	0.73	0.68	0.69	0.70	0.61	0.55	Actin[Nuc]MInt
HO-1[Cyto>_ExLo]Mint	0.95	0.89	0.87	0.82	0.82	0.88	0.84	0.84	0.88	0.75	0.72	0.69	0.70	0.71	0.64	0.61	Actin[Cyto>_ExLo]Mint
HO-1[Cyto>_ExLo]Mint	0.95	0.89	0.87	0.82	0.82	0.88	0.84	0.84	0.97	0.87	0.84	0.75	0.79	0.88	0.76	0.71	Actin[Localization>_Aggr]FormationRate
HO-1[Nuc>_Region]Mint	0.86	0.75	0.74	0.68	0.68	0.73	0.67	0.68	0.81	0.70	0.64	0.58	0.57	0.52	0.59	0.47	pFAK[Localization>_Aggr]AreaFraction
HO-1[Nuc>_Region]Mint	0.86	0.75	0.74	0.68	0.68	0.73	0.67	0.68	0.96	0.88	0.89	0.84	0.86	0.87	0.87	0.87	pFAK[Nuc>_Region]Mint
SLC3A2[Localization>_Domain]SumArea	0.86	0.81	0.84	0.91	0.93	0.86	0.88	0.96	1.21	1.21	1.18	1.13	1.07	1.19	1.17	1.03	LATS1[Localization>_Domain]Mint
LATS1[Nuc]MInt	1.16	1.13	1.11	1.03	1.03	1.11	1.06	1.00	0.82	0.86	0.91	0.96	0.91	0.89	0.93	1.02	HSPB1[Localization>_Domain]SumArea
LATS1[Cyto>_ExLo]Mint	1.25	1.22	1.21	1.13	1.09	1.21	1.17	1.06	0.86	0.81	0.84	0.91	0.93	0.86	0.88	0.96	SLC3A2[Localization>_Domain]SumArea
LATS1[Localization>_Domain]AreaFraction	0.91	0.87	0.87	0.85	0.87	0.92	0.88	0.89	0.93	0.88	0.88	0.89	0.86	0.94	0.89	0.90	LSH[Localization>_Aggr]MeanArea
LSH[Localization>_Aggr]AreaFraction	0.85	0.75	0.70	0.75	0.75	0.83	0.72	0.70	0.81	0.65	0.62	0.63	0.58	0.73	0.56	0.54	Actin[Localization>_Aggr]SumArea
LSH[Nuc>_Region]Mint	0.93	0.88	0.89	0.90	0.92	0.89	0.90	0.81	0.88	0.90	0.88	0.83	0.93	0.86	0.87	0.73	MyosinIIB[Nuc>_Region]Mint
LSH[Localization>_Aggr]AreaFraction	0.85	0.75	0.70	0.75	0.75	0.83	0.72	0.70	0.92	0.87	0.83	0.82	0.86	0.90	0.87	0.79	betaTubulin[Localization>_Aggr]AreaFraction
4HNE[Nuc>_Region]Mint	1.05	1.06	0.95	1.03	1.04	0.97	0.80	0.77	1.04	1.01	0.94	0.95	0.97	0.91	0.80	0.77	ACSL4[Nuc>_Region]Mint
4HNE[Cyto>_ExLo]Mint	1.13	1.08	1.05	1.09	1.02	0.98	0.89	0.89	1.12	1.06	1.01	1.00	1.00	0.94	0.84	0.84	ACSL4[Cyto>_ExLo]Mint
4HNE[Cyto>_ExLo]Mint	1.13	1.08	1.05	1.09	1.02	0.98	0.89	0.89	1.12	1.02	1.00	1.00	1.00	0.94	0.87	0.86	ACSL4[Localization>_Domain]Mint
4HNE[Localization>_DomainRegion]Mint	1.17	1.07	1.08	1.06	1.03	0.96	0.90	0.90	1.12	1.02	1.00	1.00	1.00	0.94	0.87	0.86	ACSL4[Localization>_Domain]Mint
ACSL4[Cyto>_ExLo]Mint	1.12	1.06	1.01	1.00	1.00	0.94	0.84	0.84	1.17	1.07	1.08	1.06	1.03	0.96	0.90	0.90	4HNE[Localization>_DomainRegion]Mint
Actin[Localization>_Aggr]SumCount	0.67	0.67	0.67	0.67	0.67	0.67	0.67	0.67	1.17	1.17	1.17	1.17	1.17	1.17	1.17	1.17	MyosinIIB[Localization>_PM]SumCount
Actin[Nuc]MInt	0.91	0.72	0.73	0.68	0.69	0.70	0.61	0.55	0.81	0.70	0.64	0.58	0.57	0.52	0.59	0.47	pFAK[Localization>_Aggr]AreaFraction
Actin[Cyto>_ExLo]Mint	0.88	0.75	0.72	0.69	0.70	0.71	0.64	0.61	0.81	0.70	0.64	0.58	0.57	0.52	0.59	0.47	pFAK[Localization>_Aggr]AreaFraction
Actin[Localization>_PMInclAggr]AreaFraction	0.99	0.93	0.91	0.88	0.88	0.84	0.86	0.80	0.81	0.70	0.64	0.58	0.57	0.52	0.59	0.47	pFAK[Localization>_Aggr]AreaFraction
MyosinIIB[Localization>_PM]AreaFraction	0.84	0.83	0.73	0.74	0.75	0.71	0.72	0.80	0.83	0.79	0.77	0.75	0.77	0.78	0.74	0.85	FAK[Localization>_PM]AreaFraction
pFAK[Nuc>_Region]Mint	0.96	0.88	0.89	0.84	0.86	0.87	0.87	0.87	1.00	1.50	1.50	1.50	1.50	1.50	1.50	1.50	FT[Localization>_Aggr]SumCount
pFAK[Nuc>_Region]Mint	0.96	0.88	0.89	0.84	0.86	0.87	0.87	0.87	0.95	0.89	0.87	0.82	0.82	0.88	0.84	0.84	HO-1[Cyto>_ExLo]Mint
pFAK[Nuc>_Region]Mint	0.96	0.88	0.89	0.84	0.86	0.87	0.87	0.87	0.95	0.87	0.86	0.82	0.82	0.90	0.86	0.86	HO-1[Localization>_Domain]Mint
pFAK[Nuc>_Region]Mint	0.96	0.88	0.89	0.84	0.86	0.87	0.87	0.87	0.88	0.75	0.72	0.69	0.70	0.71	0.64	0.61	Actin[Cyto>_ExLo]Mint
GPx4[Localization>_Domain]AreaFraction	0.89	0.86	0.87	0.79	0.85	0.83	0.85	0.87	0.85	0.84	0.85	0.79	0.82	0.78	0.81	0.87	FAK[Localization>_PM]TotalLength
GPx4[Localization>_PM]AreaFraction	0.95	0.84	0.84	0.84	0.84	0.87	0.90	0.91	0.97	0.87	0.91	0.92	0.89	0.93	0.93	0.93	HSPA5[Localization>_Aggr]AreaFraction
LAMP2[Nuc>_Region]Mint	0.98	0.85	0.88	0.88	0.96	0.88	0.87	0.80	0.98	0.88	0.89	0.92	0.95	0.93	0.91	0.88	DHODH[Localization>_Aggr]AreaFraction
mTOR[Cyto>_Region]Mint	1.00	1.00	1.00	1.05	1.05	0.98	1.01	0.83	1.00	1.00	1.00	1.00	1.00	1.00	1.00	0.50	MyosinIIB[Localization>_Aggr]SumCount
mTOR[Cyto>_Region]Mint	1.00	1.00	1.00	1.05	1.05	0.98	1.01	0.83	1.04	0.99	1.02	1.06	1.08	1.04	1.03	0.79	P-AKT 473[Cyto>_Region]Mint
mTOR[Nuc]MInt	1.01	0.98	0.95	0.96	0.97	0.92	0.90	0.79	1.10	0.99	0.93	1.03	0.98	0.98	0.92	0.75	HSPA5[Localization>_Aggr]FormationRate
P-AKT 473[Cyto>_Region]Mint	1.04	0.99	1.02	1.06	1.08	1.04	1.03	0.79	1.00	1.00	1.00	1.00	1.00	1.00	1.00	0.50	MyosinIIB[Localization>_Aggr]SumCount
P-AKT 473[Nuc]MInt	0.97	1.00	1.03	1.02	1.03	0.97	1.02	0.86	1.00	1.00	1.00	1.05	1.05	0.98	1.01	0.83	mTOR[Cyto>_Region]Mint
P-S6RP[Nuc]MInt	1.09	0.76	0.95	1.00	0.95	0.90	0.79	0.66	1.13	0.85	0.95	0.96	0.93	0.85	0.86	0.72	HIF1a[Localization>_Aggr]AreaFraction
P-S6RP[Cyto>_Region]Mint	1.20	1.04	1.28	1.34	1.32	1.25	1.13	0.94	0.87	0.95	0.87	0.84	0.87	0.88	0.92	1.01	HSPA5[Localization>_Aggr]MeanArea
ATF4[Nuc>_Region]Mint	0.91	0.86	0.86	0.91	0.92	0.81	0.86	0.77	0.93	0.88	0.89	0.90	0.92	0.89	0.90	0.81	LSH[Nuc>_Region]Mint
ATF4[Nuc>_Region]Mint	0.91	0.86	0.86	0.91	0.92	0.81	0.86	0.77	0.67	0.67	0.67	0.67	0.67	0.33	0.67	0.33	pFAK[Localization>_Aggr]SumCount
HSPA5[Localization>_Aggr]SumArea	0.90	0.87	0.84	0.83	0.85	0.86	0.85	0.94	0.79	0.79	0.73	0.77	0.71	0.70	0.71	0.89	MyosinIIB[Localization>_PM]MeanArea
HSPA5[Localization>_Aggr]SumArea	0.90	0.87	0.84	0.83	0.85	0.86	0.85	0.94	0.83	0.79	0.77	0.75	0.77	0.78	0.74	0.85	FAK[Localization>_PM]AreaFraction
HSPA5[Localization>_Aggr]SumCount	1.00	0.50	1.00	1.00	1.00	1.00	0.50	0.50	1.00	1.13	1.00	1.00	1.00	1.00	1.06	1.13	AIFM2[Localization>_Aggr]SumArea
AIFM2[Localization>_Aggr]AreaFraction	0.93	0.97	0.99	0.81	0.97	0.97	0.99	0.97	1.14	1.14	1.14	1.43	1.14	1.14	1.14	1.14	HSP90[Localization>_PM]SumCount
DHODH[Localization>_Aggr]AreaFraction	0.98	0.88	0.89	0.92	0.95	0.93	0.91	0.88	0.95	0.86	0.90	0.92	0.92	0.91	0.88	0.86	betaTubulin[Localization>_PMInclAggr]AreaFraction

Fig. S7 Feature quantities of connected nodes under the negative control condition at ρ = 0.92. Table shows the specific feature quantities of connected nodes, with their temporal changes depicted as heatmaps. Increases relative to 5 min are shown in red, whereas decreases are shown in blue. Proteins represented as Node 1 and Node 2 are connected by an edge in the network shown in Fig. 3A ("Negative control").

ρ = 0.92 AX-53802

Node 1	10	15	20	25	30	40	50	60 (min)	Node 2: 10	15	20	25	30	40	50	60 (min)	Node 2
TFR1[Localization>_Aggr]SumCount	1.00	1.00	1.00	1.00	1.00	0.75	0.75	0.75	1.05	1.11	1.05	1.05	1.11	1.21	1.26	1.32	HIF1a[Localization>_Aggr]SumArea
TFR1[Localization>_Aggr]SumCount	1.00	1.00	1.00	1.00	1.00	0.75	0.75	0.75	1.02	1.13	0.99	1.06	1.01	0.71	0.76	0.82	4HNE[Localization>_Aggr]FormationRate
TFR1[Localization>_Aggr]SumCount	1.00	1.00	1.00	1.00	1.00	0.75	0.75	0.75	0.99	0.98	1.01	0.97	0.95	0.86	0.90	0.86	ACSL4[Localization>_Domain]Mint
TFR1[Localization>_Aggr]Mint	0.93	0.94	0.98	1.01	1.13	1.25	1.38	1.31	1.01	1.01	0.99	0.93	0.87	0.76	0.75	0.78	Actin[Localization>_PM]MeanArea
TFR1[Localization>_Aggr]SumCount	1.00	1.00	1.00	1.00	1.00	0.75	0.75	0.75	1.00	1.00	1.00	1.00	1.00	0.50	0.50	0.50	pFAK[Localization>_Aggr]SumCount
HSPB1[Localization>_Domain]MeanArea	1.10	0.97	0.96	0.99	0.98	1.06	1.16	1.25	1.05	0.94	0.96	0.94	0.99	0.99	1.10	1.16	LATS1[Localization>_Domain]FormationRate
HSPB1[Localization>_Domain]SumArea	1.04	0.98	0.98	0.94	0.96	1.03	1.10	1.17	1.03	0.90	0.98	0.94	0.97	1.07	1.15	1.28	4HNE[Localization>_Domain]MeanArea
HSPB1[Localization>_Domain]AreaFraction	1.01	1.00	0.97	0.92	0.96	1.03	1.07	1.14	1.00	1.01	0.98	0.96	0.96	1.03	1.10	1.17	betaTubulin[Localization>_PM]SumArea
FT[Localization>_Domain]MeanArea	0.93	0.97	0.90	0.99	0.92	1.03	1.06	1.27	0.92	0.91	0.96	0.94	0.91	1.04	1.07	1.17	HO-1[Localization>_Domain]MeanArea
FT[Localization>_Domain]MeanArea	0.93	0.97	0.90	0.99	0.92	1.03	1.06	1.27	1.00	1.01	0.98	0.96	0.96	1.03	1.10	1.17	betaTubulin[Localization>_PM]SumArea
FT[Localization>_Domain]SumCount	2.00	2.00	2.00	2.00	2.00	2.00	2.00	1.00	0.83	0.83	0.78	0.81	0.84	0.82	0.81	0.97	FAK[Localization>_PM]AreaFraction
HIF1a[Localization>_Aggr]SumArea	1.05	1.11	1.05	1.05	1.11	1.21	1.26	1.32	0.63	1.82	0.47	1.12	1.32	2.22	4.16	3.57	keap1[Localization>_Aggr]FormationRate
HIF1a[Localization>_Aggr]SumArea	1.05	1.11	1.05	1.05	1.11	1.21	1.26	1.32	0.95	0.92	0.92	0.85	0.85	0.76	0.75	0.71	Actin[Localization>_PM]SumArea
HIF1a[Localization>_Aggr]SumArea	1.05	1.11	1.05	1.05	1.11	1.21	1.26	1.32	1.00	1.00	1.00	1.00	1.00	0.50	0.50	0.50	pFAK[Localization>_Aggr]SumCount
NCOA4[Nuc]MInt	0.99	1.04	1.04	1.11	1.17	1.02	1.08	1.01	0.96	1.05	1.05	1.22	1.28	1.11	1.20	1.07	FT[Localization>_Aggr]AreaFraction
keap1[Localization>_Domain]AreaFraction	0.98	0.92	0.91	0.93	0.95	0.94	0.97	1.17	1.00	1.00	1.00	1.00	1.00	1.00	1.00	0.86	Actin[Localization>_PMInclAggr]SumCount
keap1[Localization>_Aggr]FormationRate	0.63	1.82	0.47	1.12	1.32	2.22	4.16	3.57	0.90	1.00	0.95	1.00	0.97	1.03	1.16	1.10	betaTubulin[Localization>_Aggr]AreaFraction
HO-1[Localization>_Domain]MeanArea	0.92	0.91	0.96	0.94	0.91	1.04	1.07	1.17	1.03	0.90	0.98	0.94	0.97	1.07	1.15	1.28	4HNE[Localization>_Domain]MeanArea
HO-1[Nuc]MInt	0.94	0.91	0.85	0.83	0.84	0.85	0.85	0.79	0.93	0.83	0.76	0.67	0.63	0.56	0.56	0.50	pFAK[Localization>_Aggr]AreaFraction
HO-1[Nuc]MInt	0.94	0.91	0.85	0.83	0.84	0.85	0.85	0.79	1.13	1.16	1.19	1.21	1.19	1.21	1.18	1.23	GPx4[Localization>_PM]FormationRate
LATS1[Localization>_Domain]FormationRate	1.05	0.94	0.96	0.94	0.99	0.99	1.10	1.16	1.03	0.90	0.98	0.94	0.97	1.07	1.15	1.28	4HNE[Localization>_Domain]MeanArea
EGLN1[Nuc]MInt	0.99	0.88	0.95	1.01	0.89	0.88	0.79	0.78	1.05	1.11	1.05	1.05	1.11	1.21	1.26	1.32	HIF1a[Localization>_Aggr]SumArea
EGLN1[Cyto>_Region]Mint	1.04	0.95	1.01	1.10	0.94	0.94	0.81	0.77	0.81	1.03	0.93	0.92	1.06	1.33	1.72	1.76	GPx4[Localization>_Aggr]AreaFraction
EGLN1[Nuc]MInt	0.99	0.88	0.95	1.01	0.89	0.88	0.79	0.78	0.97	1.03	0.97	0.92	1.03	1.07	1.21	1.24	HSPA5[Localization>_Aggr]MeanArea
EGLN1[Cyto>_Region]Mint	1.04	0.95	1.01	1.10	0.94	0.94	0.81	0.77	0.97	1.03	0.97	0.92	1.03	1.07	1.21	1.24	HSPA5[Localization>_Aggr]MeanArea
4HNE[Localization>_Aggr]FormationRate	1.02	1.13	0.99	1.06	1.01	0.71	0.76	0.82	1.00	1.00	1.00	1.00	1.00	0.50	0.50	0.50	pFAK[Localization>_Aggr]SumCount
ACSL4[Cyto>_ExLo]Mint	1.01	0.99	1.03	0.99	0.97	0.85	0.90	0.83	1.00	1.00	1.00	1.00	1.00	0.75	0.75	0.75	TFR1[Localization>_Aggr]SumCount
ACSL4[Cyto>_ExLo]Mint	1.01	0.99	1.03	0.99	0.97	0.85	0.90	0.83	1.05	1.11	1.05	1.05	1.11	1.21	1.26	1.32	HIF1a[Localization>_Aggr]SumArea
ACSL4[Nuc]MInt	0.96	0.92	0.93	0.90	0.93	0.82	0.87	0.82	0.95	0.92	0.92	0.85	0.85	0.76	0.75	0.71	Actin[Localization>_PM]SumArea
ACSL4[Localization>_Domain]Mint	0.99	0.98	1.01	0.97	0.95	0.86	0.90	0.86	1.01	1.01	0.99	0.93	0.87	0.76	0.75	0.78	Actin[Localization>_PM]MeanArea
ACSL4[Cyto>_ExLo]Mint	1.01	0.99	1.03	0.99	0.97	0.85	0.90	0.83	1.00	1.00	1.00	1.00	1.00	0.50	0.50	0.50	pFAK[Localization>_Aggr]SumCount
ACSL4[Localization>_Domain]Mint	0.99	0.98	1.01	0.97	0.95	0.86	0.90	0.86	1.01	0.95	0.95	0.91	0.85	0.78	0.83	0.77	pFAK[Localization>_Aggr]MeanArea
ACSL4[Nuc]MInt	0.96	0.92	0.93	0.90	0.93	0.82	0.87	0.82	1.15	1.25	1.23	1.33	1.34	1.70	1.45	1.66	GPx4[Localization>_PMInclAggr]SumArea
ACSL4[Cyto>_ExLo]Mint	1.01	0.99	1.03	0.99	0.97	0.85	0.90	0.83	0.94	1.39	1.76	2.02	4.01	8.48	9.19	9.77	GPx4[Localization>_Aggr]FormationRate
Actin[Localization>_PMInclAggr]SumCount	1.00	1.00	1.00	1.00	1.00	1.00	1.00	0.86	1.00	1.00	1.00	1.00	1.00	1.00	1.00	1.33	betaTubulin[Localization>_PMInclAggr]SumCount
Actin[Localization>_PM]SumArea	0.95	0.92	0.92	0.85	0.85	0.76	0.75	0.71	0.97	0.85	0.79	0.73	0.67	0.58	0.58	0.48	pFAK[Localization>_Aggr]SumArea
MyosinIIB[Localization>_PM]MeanArea	0.81	0.82	0.75	0.72	0.71	0.73	0.70	0.74	0.84	0.86	0.77	0.69	0.71	0.71	0.70	0.76	pFAK[Localization>_Aggr]FormationRate
GPx4[Localization>_Aggr]FormationRate	0.94	1.39	1.76	2.02	4.01	8.48	9.19	9.77	0.93	0.94	0.98	1.01	1.13	1.25	1.38	1.31	TFR1[Localization>_Aggr]Mint
GPx4[Localization>_Aggr]FormationRate	0.94	1.39	1.76	2.02	4.01	8.48	9.19	9.77	1.05	1.11	1.05	1.05	1.11	1.21	1.26	1.32	HIF1a[Localization>_Aggr]SumArea
GPx4[Localization>_Aggr]AreaFraction	0.81	1.03	0.93	0.92	1.06	1.33	1.72	1.76	0.63	1.82	0.47	1.12	1.32	2.22	4.16	3.57	keap1[Localization>_Aggr]FormationRate
GPx4[Nuc]MInt	0.82	0.77	0.73	0.70	0.74	0.73	0.75	0.69	0.94	0.91	0.85	0.83	0.84	0.85	0.85	0.79	HO-1[Nuc]MInt
GPx4[Localization>_Aggr]FormationRate	0.94	1.39	1.76	2.02	4.01	8.48	9.19	9.77	0.99	0.98	1.01	0.97	0.95	0.86	0.90	0.86	ACSL4[Localization>_Domain]Mint
GPx4[Localization>_PMInclAggr]AreaFraction	1.21	1.22	1.25	1.29	1.44	1.59	1.59	1.68	0.95	0.92	0.92	0.85	0.85	0.76	0.75	0.71	Actin[Localization>_PM]SumArea
GPx4[Nuc]MInt	0.82	0.77	0.73	0.70	0.74	0.73	0.75	0.69	0.80	0.80	0.77	0.72	0.74	0.71	0.74	0.73	MyosinIIB[Localization>_PM]AreaFraction
GPx4[Localization>_PM]FormationRate	1.13	1.16	1.19	1.21	1.19	1.21	1.18	1.23	0.80	0.80	0.77	0.72	0.74	0.71	0.74	0.73	MyosinIIB[Localization>_PM]AreaFraction
GPx4[Localization>_Aggr]AreaFraction	0.81	1.03	0.93	0.92	1.06	1.33	1.72	1.76	0.90	1.00	0.95	1.00	0.97	1.03	1.16	1.10	betaTubulin[Localization>_Aggr]AreaFraction
GPx4[Localization>_Aggr]FormationRate	0.94	1.39	1.76	2.02	4.01	8.48	9.19	9.77	1.00	1.00	1.00	1.00	1.00	0.50	0.50	0.50	pFAK[Localization>_Aggr]SumCount
GPx4[Localization>_Aggr]AreaFraction	0.81	1.03	0.93	0.92	1.06	1.33	1.72	1.76	0.97	1.03	0.97	0.92	1.03	1.07	1.21	1.24	HSPA5[Localization>_Aggr]MeanArea
mTOR[Cyto>_Region]Mint	0.91	0.89	0.95	0.99	0.99	0.94	0.88	0.82	0.93	0.87	0.93	1.01	0.99	1.01	0.87	0.77	P-AKT 473[Cyto>_Region]Mint
mTOR[Nuc>_Region]Mint	0.87	0.73	0.84	0.85	0.88	0.83	0.83	0.80	0.90	0.83	0.87	0.88	0.93	0.90	0.90	0.88	DHODH[Localization>_Aggr]AreaFraction
P-AKT 473[Nuc]MInt	0.90	0.88	0.92	0.94	0.93	0.92	0.87	0.81	0.91	0.89	0.95	0.99	0.99	0.94	0.88	0.82	mTOR[Cyto>_Region]Mint
P-S6RP[Cyto>_Region]Mint	1.09	1.09	1.14	1.22	1.25	1.13	0.94	0.78	0.96	0.90	0.85	0.89	0.89	0.94	1.04	1.13	ACSL4[Localization>_Domain]AreaFraction
TFEB[Cyto>_Region]Mint	0.93	0.90	0.94	0.99	0.90	0.98	0.87	0.72	1.07	1.04	1.02	1.03	1.10	1.04	1.08	1.23	SLC3A2[Localization>_Domain]AreaFraction
HSPA5[Cyto>_ExLo]Mint	1.07	1.03	1.02	1.06	1.07	0.89	0.90	0.86	1.00	1.00	1.00	1.00	1.00	0.75	0.75	0.75	TFR1[Localization>_Aggr]SumCount
HSPA5[Localization>_Aggr]FormationRate	1.23	1.14	1.17	1.19	1.14	0.80	0.87	0.86	1.00	1.00	1.00	1.00	1.00	0.75	0.75	0.75	TFR1[Localization>_Aggr]SumCount
HSPA5[Localization>_Aggr]MeanArea	0.97	1.03	0.97	0.92	1.03	1.07	1.21	1.24	1.05	1.11	1.05	1.05	1.11	1.21	1.26	1.32	HIF1a[Localization>_Aggr]SumArea
HSPA5[Localization>_Aggr]MeanArea	0.97	1.03	0.97	0.92	1.03	1.07	1.21	1.24	0.63	1.82	0.47	1.12	1.32	2.22	4.16	3.57	keap1[Localization>_Aggr]FormationRate
HSPA5[Cyto>_ExLo]Mint	1.07	1.03	1.02	1.06	1.07	0.89	0.90	0.86	0.92	0.91	0.96	0.94	0.91	1.04	1.07	1.17	HO-1[Localization>_Domain]MeanArea
HSPA5[Localization>_Aggr]MeanArea	0.97	1.03	0.97	0.92	1.03	1.07	1.21	1.24	0.99	0.99	0.89	0.96	1.01	1.04	1.14	1.18	ACSL4[Localization>_Domain]MeanArea
HSPA5[Localization>_Domain]SumCount	1.00	2.00	1.00	1.00	1.00	1.00	1.00	1.00	1.00	0.50	1.00	1.00	1.00	1.00	1.00	1.00	Actin[Localization>_Aggr]SumCount
HSPA5[Localization>_Aggr]MeanArea	0.97	1.03	0.97	0.92	1.03	1.07	1.21	1.24	1.00	1.01	0.98	0.96	0.96	1.03	1.10	1.17	betaTubulin[Localization>_PM]SumArea
HSPA5[Cyto>_ExLo]Mint	1.07	1.03	1.02	1.06	1.07	0.89	0.90	0.86	1.00	1.00	1.00	1.00	1.00	0.50	0.50	0.50	pFAK[Localization>_Aggr]SumCount
HSPA5[Localization>_Aggr]FormationRate	1.23	1.14	1.17	1.19	1.14	0.80	0.87	0.86	1.00	1.00	1.00	1.00	1.00	0.50	0.50	0.50	pFAK[Localization>_Aggr]SumCount
NRF2[Localization>_Domain]MeanArea	0.87	0.92	0.85	0.87	0.90	1.08	1.04	1.28	0.93	0.97	0.90	0.99	0.92	1.03	1.06	1.27	FT[Localization>_Domain]MeanArea
NRF2[Localization>_Domain]SumArea	0.85	0.83	0.86	0.89	0.88	0.99	0.97	1.20	0.92	0.91	0.96	0.94	0.91	1.04	1.07	1.17	HO-1[Localization>_Domain]MeanArea
AIFM2[Nuc>_Region]Mint	0.90	0.87	0.86	0.85	0.93	0.90	0.94	0.88	0.91	0.87	0.87	0.83	0.90	0.91	0.94	0.85	GPx4[Cyto>_ExLo]Mint
DHODH[Localization>_Domain]MeanArea	0.98	0.92	0.94	1.00	1.01	0.96	1.08	1.16	0.95	0.94	0.86	0.95	0.99	0.98	1.10	1.15	ACSL4[Localization>_Domain]SumArea

Fig. S8 Feature quantities of connected nodes under the AX-53802–treated condition at ρ = 0.92.

Table shows the specific feature quantities of connected nodes, with their temporal changes depicted as heatmaps. Increases relative to 5 min are shown in red, whereas decreases are shown in blue. Proteins represented as Node 1 and Node 2 are connected by an edge in the network shown in Fig. 3A (“AX-53802”). GPX4 exhibited earlier changes compared with other proteins. TfR1 aggregate numbers decreased, whereas mean intensity in TfR1 aggregation regions increased.

ρ = 0.92 AX-53802 + Fer1

	Node 1								Node 2								
	10	15	20	25	30	40	50	60 (min)	10	15	20	25	30	40	50	60 (min)	
TFR1[Localization>_Aggr]SumArea	1.11	1.13	1.04	1.04	1.02	1.02	0.93	1.00	0.93	0.88	0.97	0.92	0.96	0.97	1.07	0.99	HO-1[Localization>_Domain]SumArea
TFR1[Nuc>_Region]Mint	0.95	0.96	0.92	0.88	0.99	0.89	0.95	0.80	0.96	0.95	0.93	0.89	0.94	0.91	0.93	0.85	GPx4[Localization>_PM]Mint
HSPB1[Nuc>_Region]Mint	0.93	0.81	1.00	0.92	0.95	1.00	0.92	0.86	0.95	0.86	0.99	0.96	0.96	0.96	0.92	0.89	LSH[Cyto]MInt
HSPB1[Nuc>_Region]Mint	0.93	0.81	1.00	0.92	0.95	1.00	0.92	0.86	0.96	0.84	0.97	0.92	0.94	0.95	0.90	0.88	LSH[Nuc>_Region]Mint
ATG7[Nuc>_Region]Mint	1.15	1.09	1.01	0.91	0.97	0.90	0.91	0.91	1.12	1.07	1.03	0.87	0.89	0.89	0.91	0.86	HO-1[Cyto>_Region]Mint
ATG7[Nuc>_Region]Mint	1.15	1.09	1.01	0.91	0.97	0.90	0.91	0.91	1.14	1.09	1.03	0.94	0.94	0.89	0.94	0.86	MyosinIIB[Localization>_Aggr]SumArea
HSP90[Cyto>_ExLo]Mint	1.14	1.06	1.08	1.05	0.98	0.84	0.91	0.81	1.03	1.00	1.01	1.04	0.97	0.87	0.93	0.90	ACSL4[Cyto>_ExLo]Mint
HSP90[Cyto>_ExLo]Mint	1.14	1.06	1.08	1.05	0.98	0.84	0.91	0.81	1.03	0.99	1.01	1.02	0.96	0.87	0.93	0.92	ACSL4[Localization>_Domain]Mint
HSP90[Cyto>_UpperNucl]Mint	1.21	1.11	1.09	1.03	0.97	0.83	0.86	0.80	1.14	1.09	1.03	0.94	0.94	0.89	0.94	0.86	MyosinIIB[Localization>_Aggr]SumArea
HSP90[Cyto>_UpperNucl]Mint	1.21	1.11	1.09	1.03	0.97	0.83	0.86	0.80	1.28	1.05	1.11	1.09	1.04	0.79	0.67	0.73	HSPA5[Localization>_Aggr]FormationRate
HSP90[Cyto>_ExLo]Mint	1.14	1.06	1.08	1.05	0.98	0.84	0.91	0.81	0.88	1.00	1.00	1.00	1.00	1.14	1.05	1.14	AIFM2[Localization>_Aggr]MeanArea
HO-1[Nuc>_Region]Mint	1.03	0.90	0.89	0.75	0.80	0.79	0.81	0.76	1.02	0.92	0.91	0.84	0.90	0.86	0.91	0.80	MyosinIIB[Localization>_Aggr]FormationRate
HO-1[Cyto>_Region]Mint	1.12	1.07	1.03	0.87	0.89	0.89	0.91	0.86	1.14	1.09	1.03	0.94	0.94	0.89	0.94	0.86	MyosinIIB[Localization>_Aggr]SumArea
LSH[Localization>_Aggr]AreaFraction	0.93	0.80	0.91	0.92	0.87	0.79	0.78	0.81	0.91	0.83	0.88	0.93	0.84	0.77	0.83	0.80	4HNE[Localization>_Aggr]AreaFraction
EGLN1[Nuc>_Region]Mint	1.01	0.91	0.86	0.96	0.98	0.88	0.77	0.68	0.97	0.93	0.90	0.99	0.96	0.88	0.87	0.83	ATF4[Cyto]MInt
EGLN1[Nuc>_Region]Mint	1.01	0.91	0.86	0.96	0.98	0.88	0.77	0.68	1.03	0.96	0.99	0.99	1.02	0.95	0.90	0.80	AIFM2[Localization>_DomainRegion]Mint
EGLN1[Cyto>_Region]Mint	1.03	0.96	0.94	1.06	1.04	0.95	0.79	0.73	1.03	0.96	0.99	0.99	1.02	0.95	0.90	0.80	AIFM2[Localization>_DomainRegion]Mint
4HNE[Cyto>_ExLo]Mint	1.01	0.92	0.97	1.01	0.94	0.86	0.92	0.88	1.00	0.91	0.97	1.07	0.97	0.87	0.89	0.85	LSH[Localization>_Aggr]FormationRate
4HNE[Nuc]MInt	0.97	0.88	0.86	0.97	0.92	0.79	0.86	0.83	0.99	0.94	0.93	0.99	0.95	0.83	0.89	0.85	ACSL4[Nuc>_Region]Mint
4HNE[Cyto>_ExLo]Mint	1.01	0.92	0.97	1.01	0.94	0.86	0.92	0.88	1.03	1.00	1.01	1.04	0.97	0.87	0.93	0.90	ACSL4[Cyto>_ExLo]Mint
4HNE[Localization>_DomainRegion]Mint	1.04	0.95	0.99	1.00	0.95	0.87	0.93	0.93	1.03	0.99	1.01	1.02	0.96	0.87	0.93	0.92	ACSL4[Localization>_Domain]Mint
4HNE[Localization>_Aggr]FormationRate	1.05	1.11	1.09	1.23	0.93	0.77	0.95	0.90	1.04	1.08	1.04	1.08	0.92	0.88	0.96	0.92	LAMP2[Localization>_Aggr]SumCount
4HNE[Nuc]MInt	0.97	0.88	0.86	0.97	0.92	0.79	0.86	0.83	0.97	0.93	0.90	0.99	0.96	0.88	0.87	0.83	ATF4[Cyto]MInt
ACSL4[Cyto>_ExLo]Mint	1.03	1.00	1.01	1.04	0.97	0.87	0.93	0.90	1.14	1.07	1.06	1.05	0.97	0.85	0.90	0.80	HSP90[Localization>_PM]Mint
ACSL4[Nuc>_Region]Mint	0.99	0.94	0.93	0.99	0.95	0.83	0.89	0.85	1.01	0.92	0.97	1.01	0.94	0.86	0.92	0.88	4HNE[Cyto>_ExLo]Mint
Actin[Localization>_PM]AreaFraction	0.94	0.89	0.81	0.86	0.80	0.80	0.80	0.78	0.89	0.77	0.76	0.72	0.74	0.69	0.73	0.70	MyosinIIB[Localization>_PMInclAggr]AreaFraction
Actin[Localization>_PM]AreaFraction	0.94	0.89	0.81	0.86	0.80	0.80	0.80	0.78	0.88	0.87	0.79	0.79	0.71	0.68	0.71	0.65	pFAK[Localization>_Aggr]SumArea
MyosinIIB[Localization>_PMInclAggr]SumArea	0.89	0.83	0.80	0.76	0.78	0.70	0.80	0.80	0.97	0.96	0.94	0.91	0.94	0.87	0.94	0.95	betaTubulin[Localization>_PMInclAggr]MeanArea
MyosinIIB[Nuc>_Region]Mint	0.98	0.83	0.80	0.77	0.78	0.76	0.78	0.59	0.97	0.87	0.82	0.83	0.80	0.82	0.80	0.64	pFAK[Localization>_Aggr]FormationRate
MyosinIIB[Localization>_PMInclAggr]Mint	1.05	0.97	0.92	0.90	0.91	0.88	0.90	0.73	0.97	0.87	0.82	0.83	0.80	0.82	0.80	0.64	pFAK[Localization>_Aggr]FormationRate
betaTubulin[Localization>_PMInclAggr]SumCount	1.00	1.00	1.00	1.00	1.00	1.00	1.33	1.33	1.00	1.00	1.00	1.00	1.00	1.00	0.50	0.50	pFAK[Localization>_Aggr]SumCount
pFAK[Nuc>_Region]Mint	0.94	0.90	0.88	0.90	0.81	0.83	0.85	0.78	0.94	0.89	0.81	0.86	0.80	0.80	0.80	0.78	Actin[Localization>_PM]AreaFraction
pFAK[Nuc>_Region]Mint	0.94	0.90	0.88	0.90	0.81	0.83	0.85	0.78	1.06	1.10	1.13	1.10	1.15	1.14	1.13	1.14	GPx4[Localization>_PM]FormationRate
GPx4[Localization>_PM]FormationRate	1.06	1.10	1.13	1.10	1.15	1.14	1.13	1.14	0.94	0.89	0.81	0.86	0.80	0.80	0.80	0.78	Actin[Localization>_PM]AreaFraction
GPx4[Nuc]MInt	0.88	0.81	0.77	0.71	0.77	0.72	0.74	0.66	0.89	0.77	0.76	0.72	0.74	0.69	0.73	0.70	MyosinIIB[Localization>_PMInclAggr]AreaFraction
GPx4[Nuc]MInt	0.88	0.81	0.77	0.71	0.77	0.72	0.74	0.66	0.98	0.83	0.80	0.77	0.78	0.76	0.78	0.59	MyosinIIB[Nuc>_Region]Mint
GPx4[Localization>_PMInclAggr]SumArea	1.15	1.27	1.24	1.34	1.32	1.46	1.44	1.51	0.89	0.77	0.76	0.72	0.74	0.69	0.73	0.70	MyosinIIB[Localization>_PMInclAggr]AreaFraction
GPx4[Nuc]MInt	0.88	0.81	0.77	0.71	0.77	0.72	0.74	0.66	0.84	0.79	0.74	0.71	0.66	0.64	0.64	0.52	pFAK[Localization>_Aggr]AreaFraction
GPx4[Localization>_PMInclAggr]SumArea	1.15	1.27	1.24	1.34	1.32	1.46	1.44	1.51	0.98	0.93	0.94	0.91	0.89	0.88	0.89	0.85	pFAK[Localization>_Aggr]Mint
P-S6RP[Nuc]MInt	0.93	0.90	0.82	0.80	0.79	0.70	0.64	0.64	0.88	0.87	0.79	0.79	0.71	0.68	0.71	0.65	pFAK[Localization>_Aggr]SumArea
P-S6RP[Nuc]MInt	0.93	0.90	0.82	0.80	0.79	0.70	0.64	0.64	1.15	1.27	1.24	1.34	1.32	1.46	1.44	1.51	GPx4[Localization>_PMInclAggr]SumArea
P-S6RP[Cyto>_Region]Mint	1.15	1.19	1.21	1.17	1.21	1.06	0.95	0.95	0.97	0.89	0.93	0.90	0.89	0.97	1.14	1.09	HSPA5[Localization>_Domain]MeanArea
ATF4[Nuc>_Region]Mint	0.94	0.85	0.80	0.90	0.90	0.80	0.78	0.76	0.97	0.88	0.86	0.97	0.92	0.79	0.86	0.83	4HNE[Nuc]MInt
HSPA5[Nuc>_Region]Mint	1.13	1.02	1.02	1.01	0.97	0.89	0.83	0.86	1.21	1.11	1.09	1.03	0.97	0.83	0.86	0.80	HSP90[Cyto>_UpperNucl]Mint
HSPA5[Nuc>_Region]Mint	1.13	1.02	1.02	1.01	0.97	0.89	0.83	0.86	1.14	1.07	1.06	1.05	0.97	0.85	0.90	0.80	HSP90[Localization>_PM]Mint
HSPA5[Cyto]MInt	1.10	1.00	1.01	1.04	1.00	0.90	0.84	0.85	1.14	1.07	1.06	1.05	0.97	0.85	0.90	0.80	HSP90[Localization>_PM]Mint
HSPA5[Localization>_Aggr]FormationRate	1.28	1.05	1.11	1.09	1.04	0.79	0.67	0.73	1.14	1.07	1.06	1.05	0.97	0.85	0.90	0.80	HSP90[Localization>_PM]Mint
HSPA5[Localization>_Aggr]SumArea	0.92	1.00	0.87	0.85	0.80	0.89	0.99	0.95	0.95	0.99	0.86	0.89	0.83	0.89	1.00	0.96	keap1[Localization>_Domain]SumArea
AIFM2[Localization>_Aggr]MeanArea	0.88	1.00	1.00	1.00	1.00	1.14	1.05	1.14	1.14	1.07	1.06	1.05	0.97	0.85	0.90	0.80	HSP90[Localization>_PM]Mint

Fig. S9 Feature quantities of connected nodes under AX-53802 and Fer1 cotreatment at ρ = 0.92.

Table shows the specific feature quantities of connected nodes, with their temporal changes depicted as heatmaps. Increases relative to 5 min are shown in red, whereas decreases are shown in blue. Proteins represented as Node 1 and Node 2 are connected by an edge in the network shown in Fig. 3A (“AX-53802 + Fer1”).

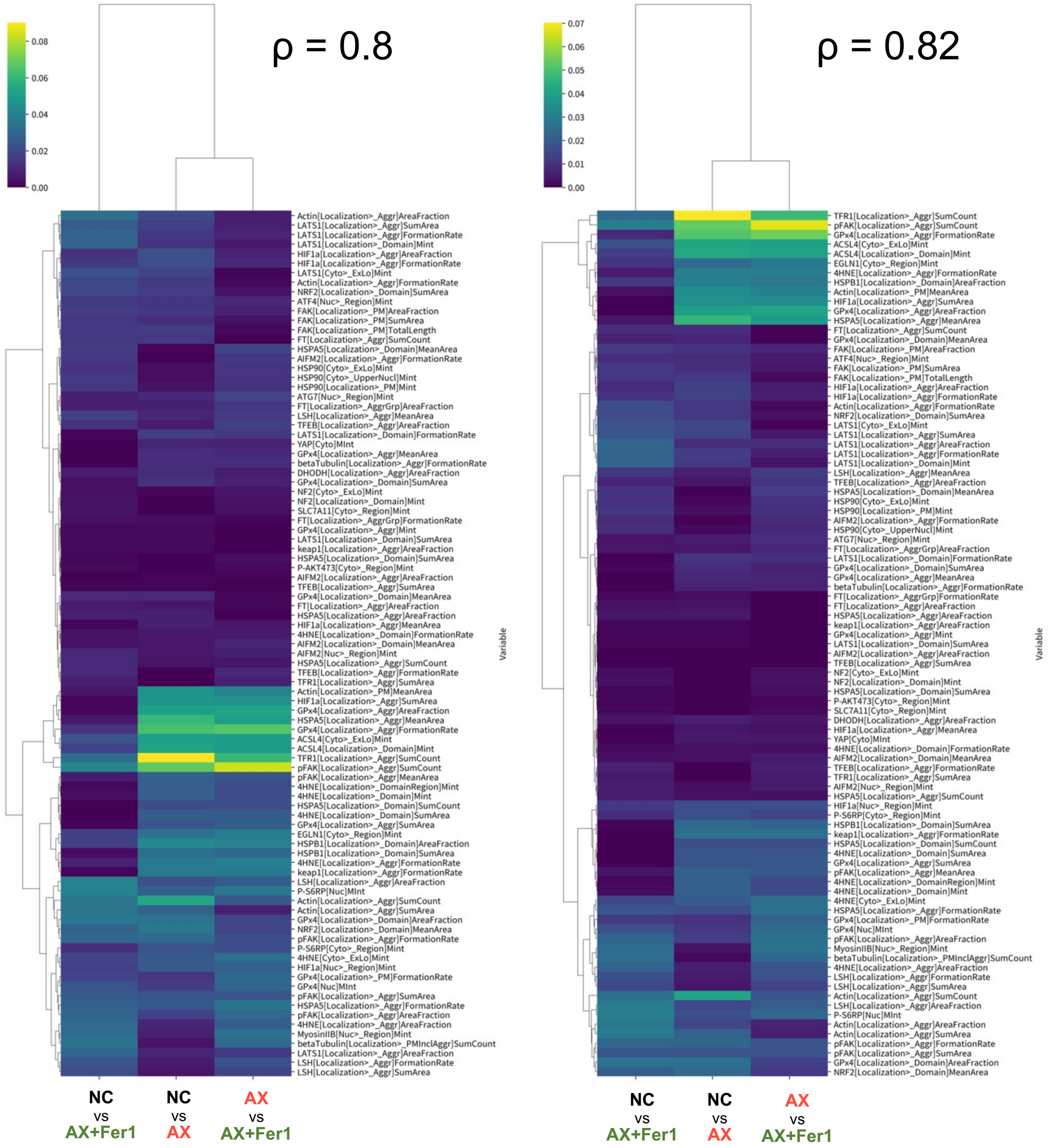

Fig. S10 Heatmap of correlation anomaly scores (for ρ values of 0.80 and 0.82). Correlation anomaly scores were calculated between the two conditions indicated at the bottom of the heatmap. Higher values indicate more significant alterations in connectivity and correlation strength of a node's first neighbors (directly connected nodes). AX: AX-53802, NC: negative control, Fer1: ferrostatin-1.

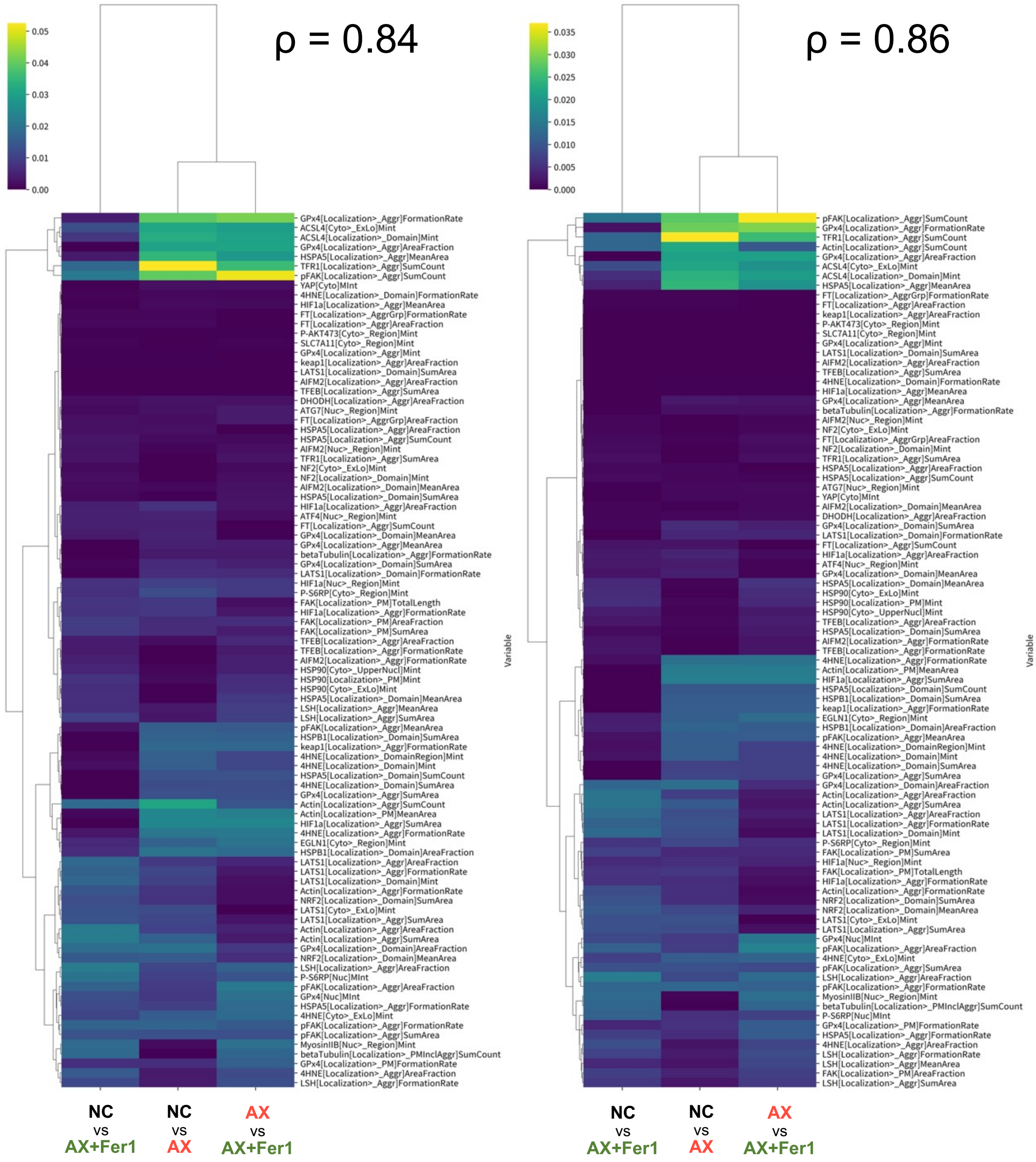

Fig. S10 Heatmap of correlation anomaly scores (for ρ values of 0.84 and 0.86).

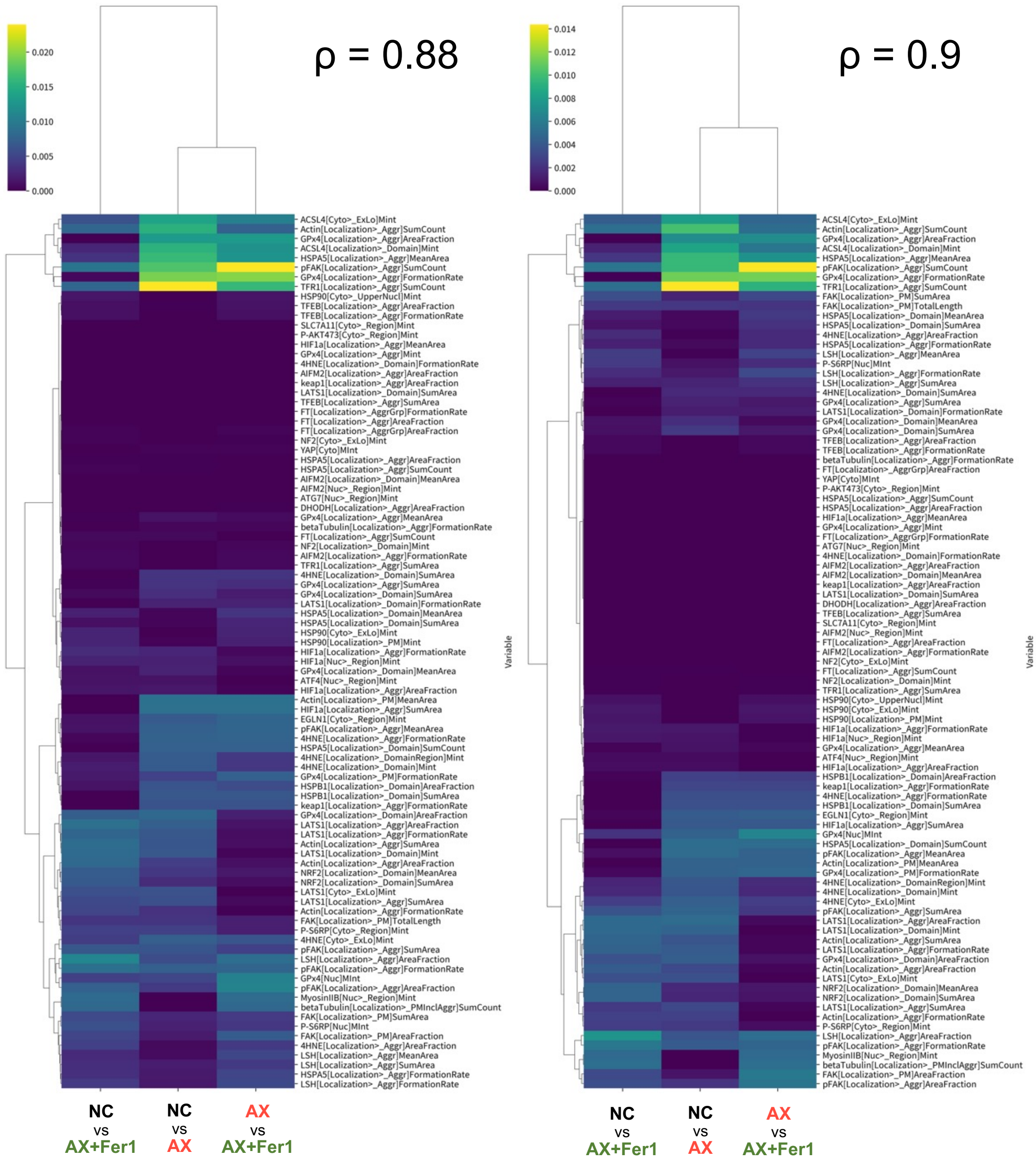

Fig. S10 Heatmap of correlation anomaly scores (for ρ values of 0.88 and 0.90).

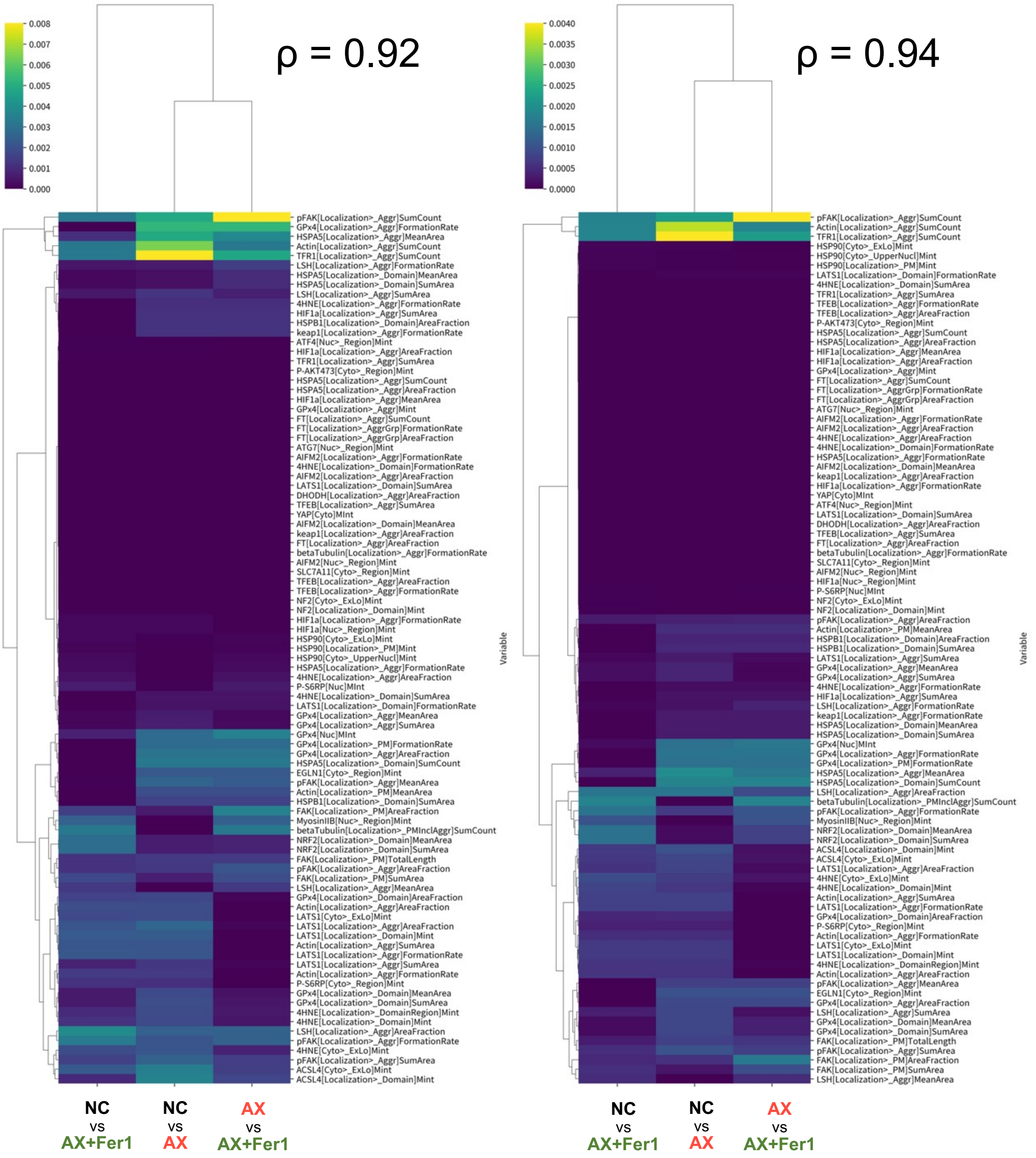

Fig. S10 Heatmap of correlation anomaly scores (for ρ values of 0.92 and 0.94).

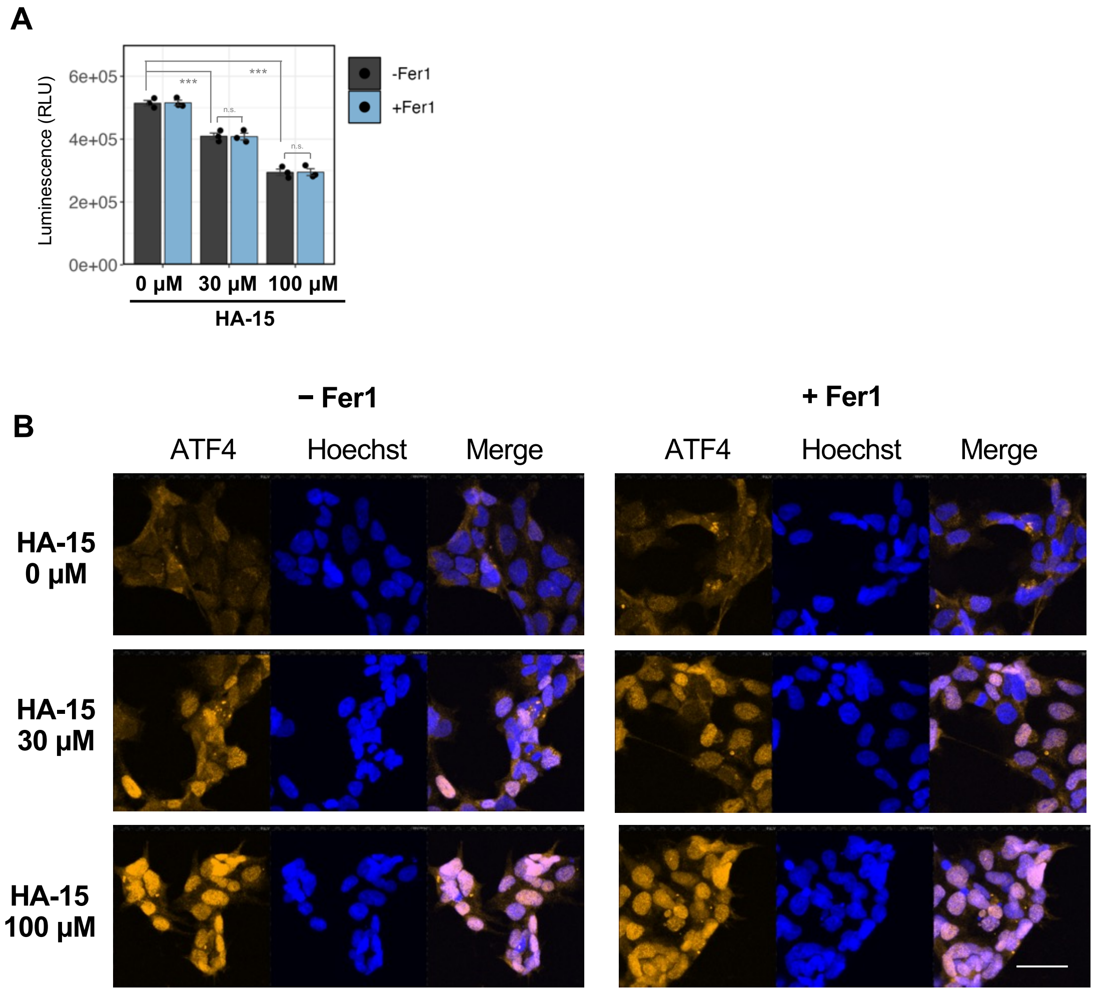

Fig. S11 The HSPA5 inhibitor HA-15 did not induce ferroptosis in HEK293 cells.

(A) The viability of HEK293 cells treated with HA-15 for 24 h was measured using a CellTiter-Glo assay. A concentration-dependent decrease in viability is observed with HA-15 treatment; however, this reduction is not prevented by Ferrostatin-1. These findings indicate that HSPA5 inhibition alone does not induce ferroptosis, demonstrating that the MoA of AX-53802 is distinctly different from the mechanism of HSPA5 inhibition. Data are shown as mean ± SEM (n = 3). ***$P < 0.001$; ns: not significant (Tukey's test).

(B) Immunofluorescence analysis showing nuclear accumulation of ATF4, an ER stress marker, in cells treated with HA-15 for 3 h. Inhibition of the ER chaperone HSPA5 using HA-15 induces misfolded protein accumulation in the ER, leading to ER stress. Results confirm effective HSPA5 inhibition under the tested conditions. Scale bar: 50 μm.

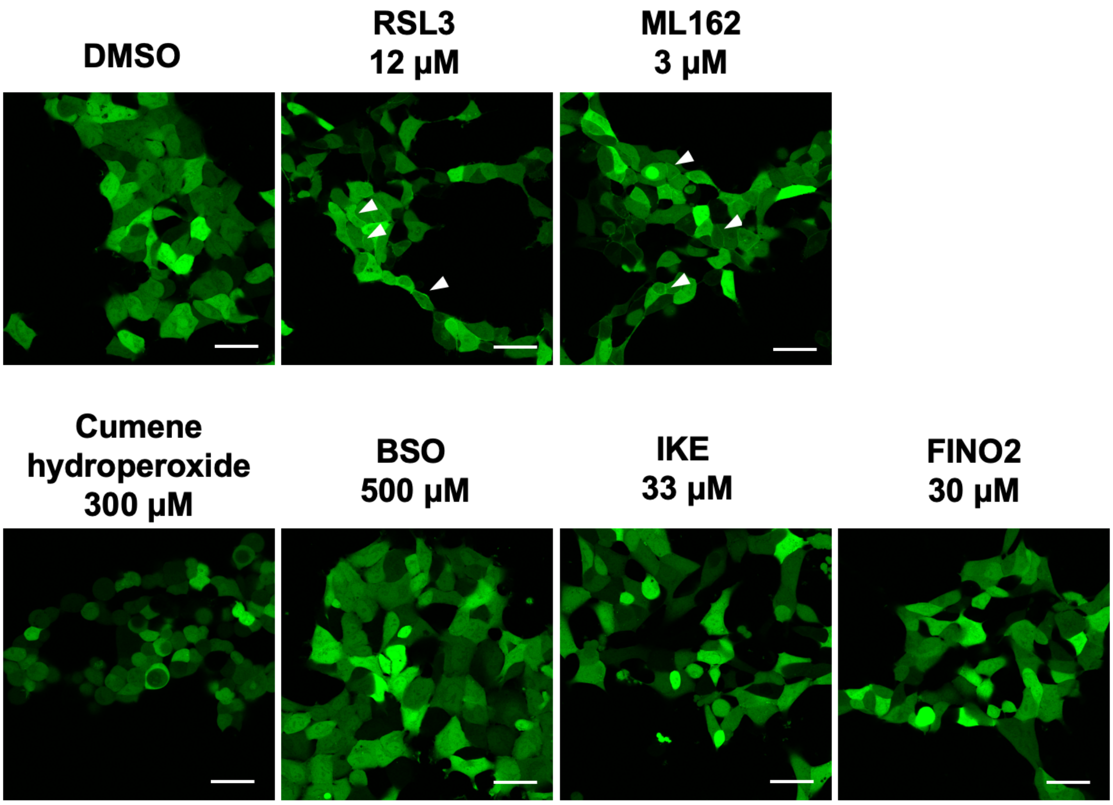

Fig. S12 Images of HEK293 cells stably expressing GFP-GPX4 and treated with ferroptosis inducers.

HEK293 cells treated with the covalent GPX4 inhibitors RSL3 and ML162 exhibited GFP-GPX4 membrane translocation, whereas those treated with other inducers did not. Cells were treated with RSL3 (12 µM), ML162 (3 µM), cumene hydroperoxide (300 µM), BSO (500 µM), imidazole ketone erastin (IKE; 33 µM), or FINO2 (30 µM) for 3 h. Images were captured using confocal laser-scanning microscopy (×40 objective). Scale bars: 50 µm.

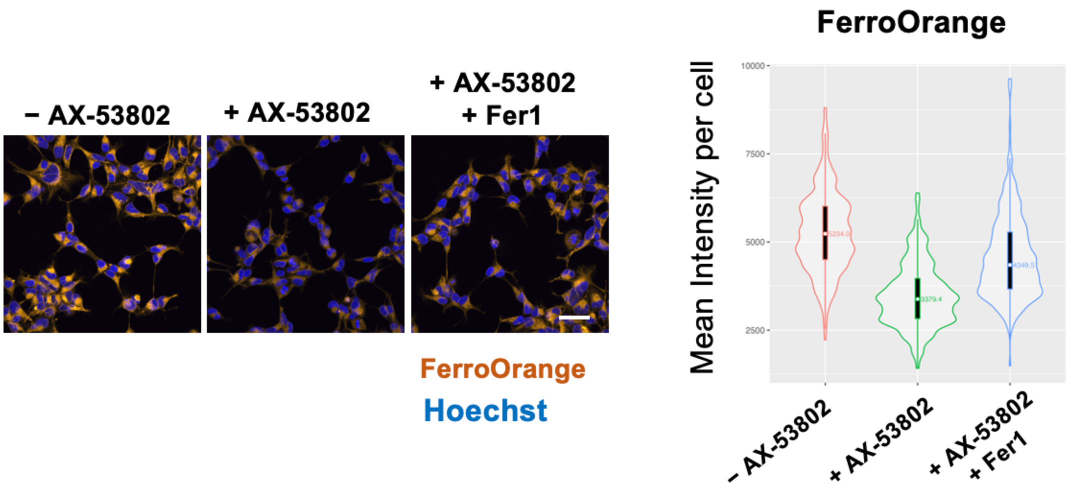

Fig. S13 Quantification of labile iron levels 3 h post-treatment.

Quantification of labile iron levels in untreated HEK293 cells or those treated with 0.8 μM AX-53802 for 3 h. Post-treatment, cells were incubated with FerroOrange probe for 30 min. Labile iron content decreased under AX-53802 treatment, an effect rescued by Fer1 addition. Scale bar: 50 μm. Right panel: quantification of mean FerroOrange intensity per cell.

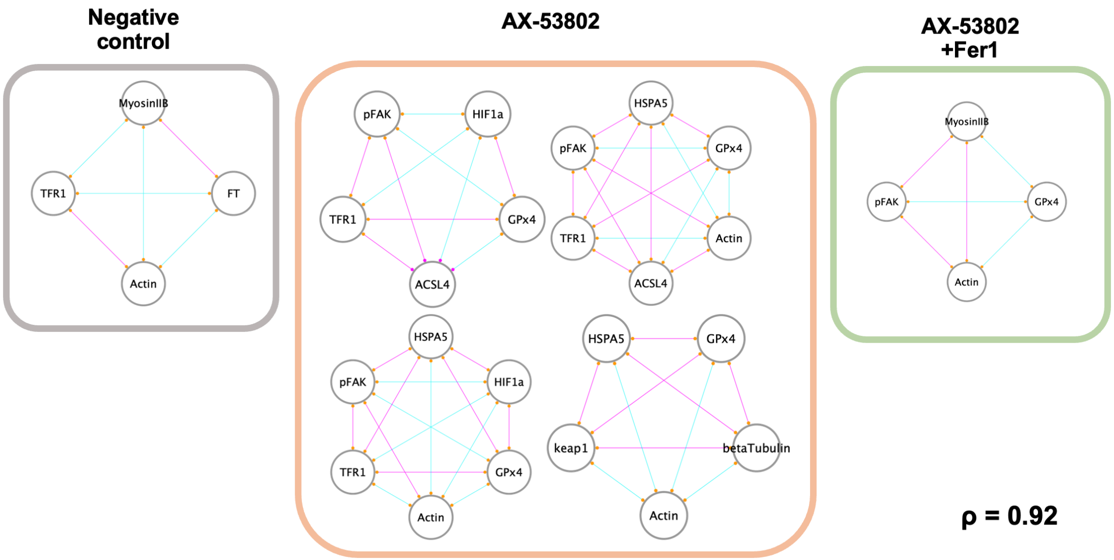

Fig. S14 Clusters extracted from the covariation network at ρ = 0.92.

Densely connected clusters were obtained from the covariation networks at ρ = 0.92 (Fig. 3A) using the Overlapping Cluster Generator algorithm.

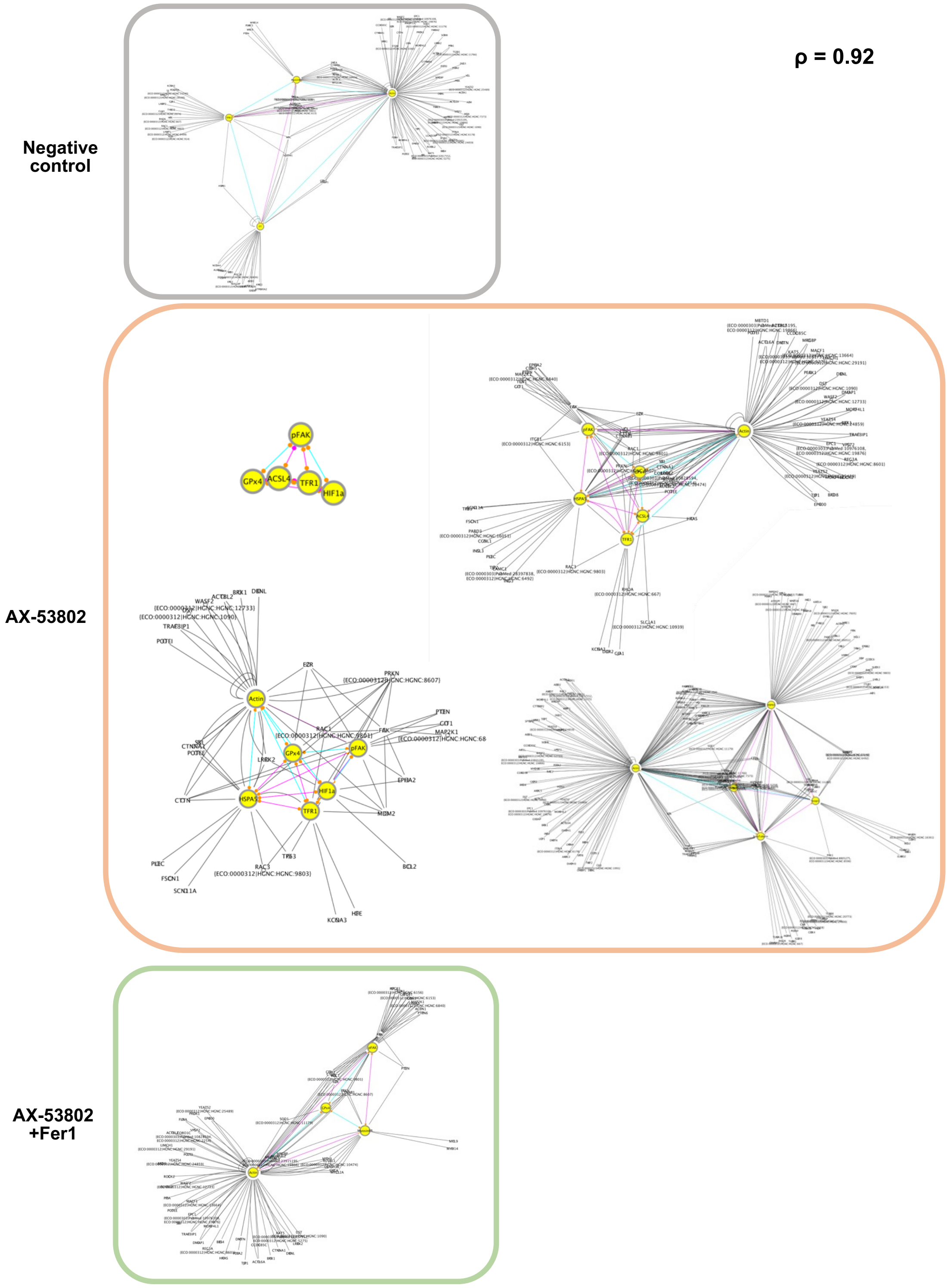

Fig. S15 Expanded networks at ρ = 0.92.

Clusters extracted via graph clustering (Fig. S14) were expanded using a protein–protein interaction database (BioGrid) to target the first neighbors of cluster components.

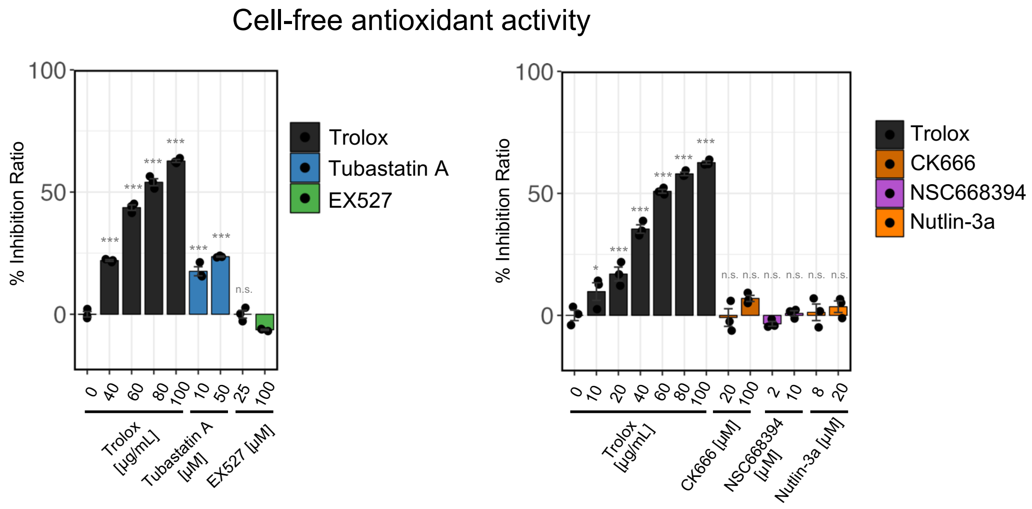

Fig. S16 Cell-free antioxidant activity assay of ferroptosis-suppressing compounds.

Cell-free antioxidant activity of tubastatin A (10 and 50 μM), EX527 (25 and 100 μM), CK666 (20 and 100 μM), NSC668394 (2 and 10 μM), and nutlin-3a (8 and 20 μM) were evaluated via a DPPH antioxidant assay, with Trolox used as a positive control. Tubastatin A exhibited mild antioxidant activity comparable to 40 μg/mL Trolox, whereas other inhibitors showed less antioxidant activity compared with 10 μg/mL Trolox. Data: means ± SEMs ($n = 3$). *$P < 0.05$; ***$P < 0.001$; ns: not significant (Dunnett's test).

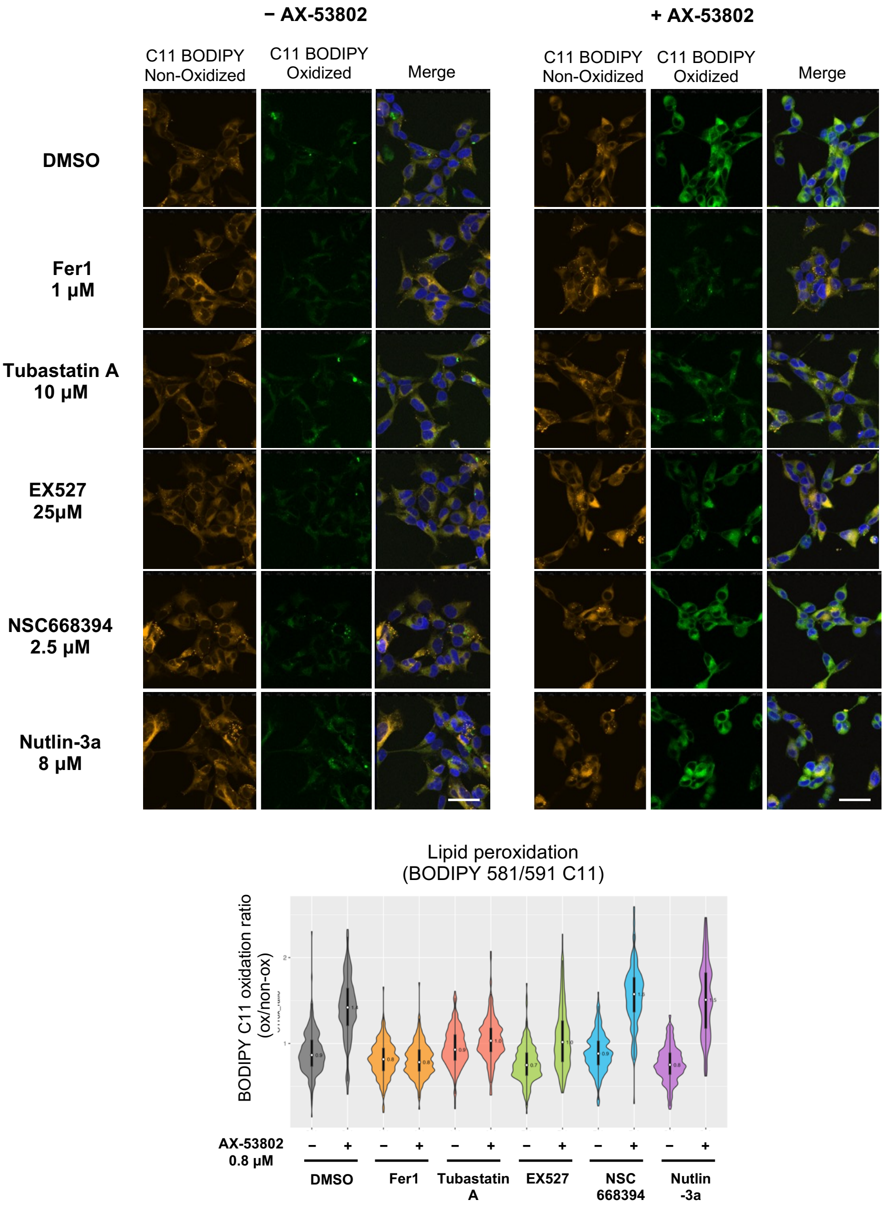

Fig. S17 Effects of ferroptosis-suppressing compounds on lipid peroxidation.

HEK293 cells were pretreated with BODIPY-C11 probe for 1 h, followed by cotreatment with 0.8 μM AX-53802 and the indicated compounds for 2 h. Cells were observed using a confocal laser-scanning microscope (×40 objective). Oxidization level of the BODIPY-C11 probe was partially suppressed by tubastatin A and EX527 but not by NSC66839 or nutlin-3a. Scale bars: 50 μm. Oxidation ratio of BODIPY-C11 was quantified per cell and represented as a violin plot. Absolute values of effect sizes were calculated between AX-53802 alone [AX-53802 + dimethylsulfoxide (DMSO)] vs. cotreatment of AX-53802 with each combination drug using Cliff's Delta: 0.852 (+Fer1), 0.643 (+tubastatin A), 0.53 (+EX527), 0.23 (+NSC668394), and 0.137 (+nutlin-3a).

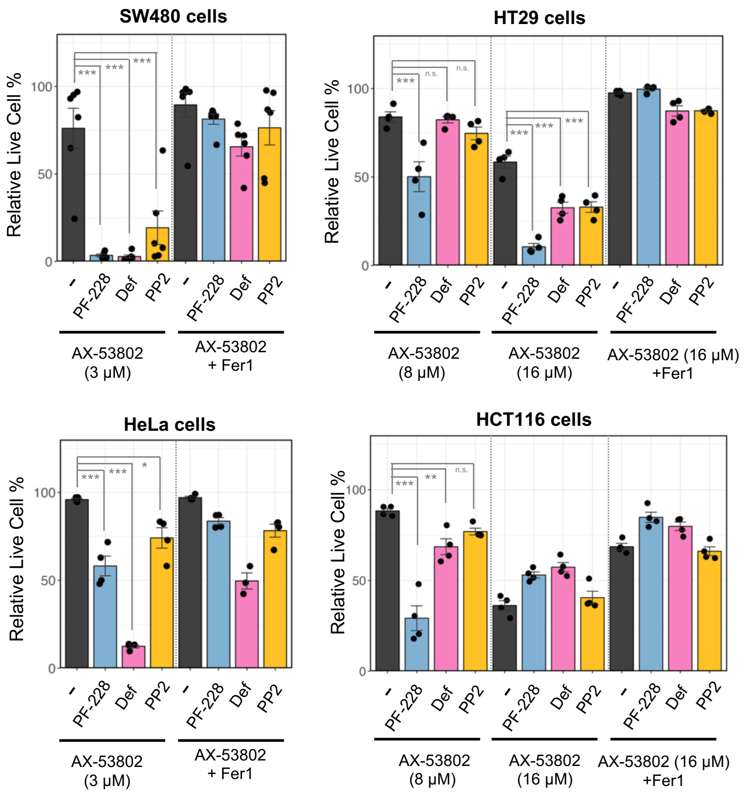

Fig. S18 Assay of cancer cell death after cotreatment with AX-53802 and FAK/Src inhibitors. SW480, HT29, HCT116, and HeLa cells were cotreated with AX-53802 and FAK/Src inhibitors for 24 h, and cell death was detected via PI staining. The percentage of live cells (PI-negative) to the total number of cells (Hoechst-stained) was calculated and data were normalized to the control (without AX-53802). Data are presented as means ± SEMs (n = 3). *P < 0.05; ***P < 0.001; ns: not significant (Tukey's test).

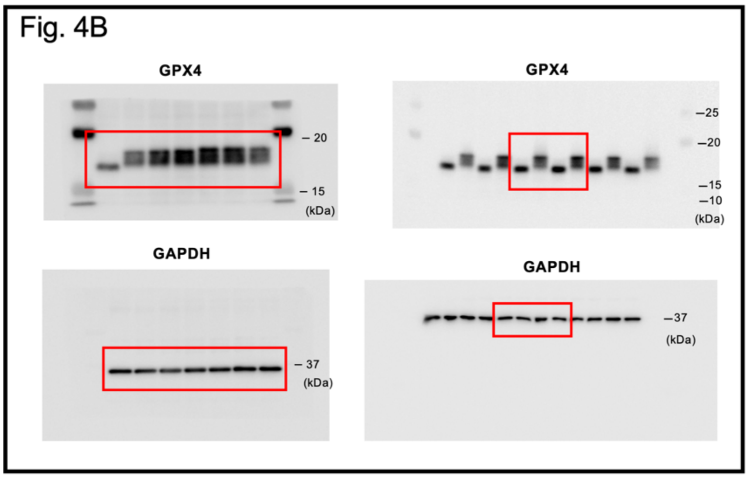

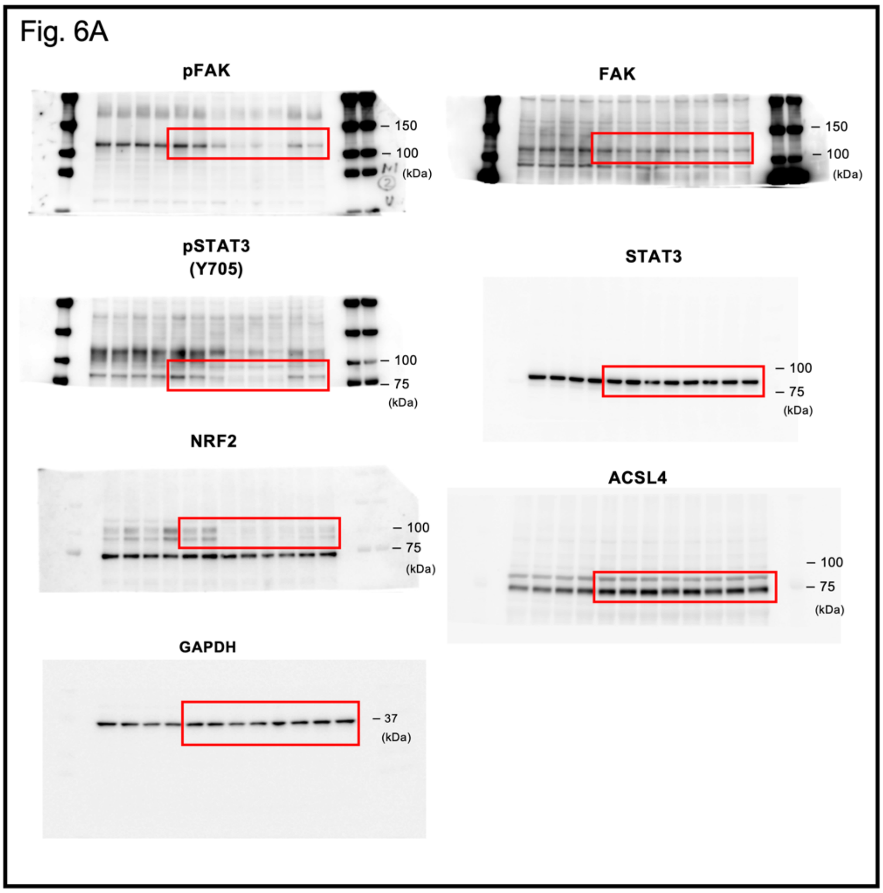

Fig. S19 Uncropped and unedited blot images.

Supplementary Discussion

Comparison of Image-Based Network Analysis Methods

Image-based network analysis methods, such as DiSWOP[2] and PLOM-CON, share the common features such as utilizing protein-level data and single-cell image analysis; however, differ considerably in their analytical approaches, making them suited for analyzing distinct sample types. DiSWOP links multiplex protein staining data within individual cells to provide a detailed snapshot of protein interactions (dependencies) at a specific time point. This makes it highly effective for clustering and phenotyping heterogeneous samples, such as tissue sections. In contrast, PLOM-CON does not link all protein data within individual cells; instead, it captures temporal protein variations over a specific time window and uses these covariations to establish network edges. In PLOM-CON, feature quantities are initially quantified at the single-cell level; however, partway through the analysis, the data were aggregated using the median, shifting the focus from the diversity within a single sample to the behavior of most cells over time. This approach is deemed adequate for systems using cultured cells, where less variability among individual cells is expected.

For estimating the mechanism of action (MoA) of a drug, PLOM-CON was selected because of its practical advantages in sample preparation and its suitability for relatively homogeneous cultured cell models. Unlike DiSWOP[2], which requires complex multiplex staining techniques, such as multiepitope-ligand cartography (MELC)[3], and precise image alignment for single-cell data, PLOM-CON has a simplified process involving staining individual cells for each protein separately. Although DiSWOP is well-suited for analyzing heterogeneous samples, it may provide limited insights or even pose challenges to interpret results pertaining to systems in which stained proteins exhibit uniform distribution across the sample. In contrast, the ability of PLOM-CON to detect temporal changes in response to drug treatment offers more interpretable results and is better suited for estimating the drug's MoA in relatively homogeneous cultured cell systems.

Comparison of Network Inference Methods

For PLOM-CON analysis, we utilized a graphical lasso for network inference rather than traditional correlation-based methods, such as Pearson's correlation. This approach allows for the capture of partial correlations and elimination of spurious correlations. It also enables the mathematical removal of the effects of non-specific or false staining, which are common challenges in immunofluorescence (IF) experiments. Moreover, other methods capable of capturing nonlinear relationships, such as Mutual Information (MI)[4], Maximal Information Coefficient (MIC)[5], Distance Correlation[6], and Hilbert-Schmidt Independence Criterion

(HSIC)[7], have been reported; however, these techniques are not suitable for our dataset due to the limited number of time points (10 in this study). Similarly, the use of Time-Varying Graphical Lasso (TVGL)[8], designed for analyzing temporal data, is limited by the minimal time points in our dataset. However, future studies using systems capable of acquiring more detailed time-course data, for example through live-cell imaging, could enable the application of these advanced network inference methods and provide more comprehensive insights into network analyses.

Supplementary References

1. Noguchi, Y. *et al.* Microscopic image-based covariation network analysis for actin scaffold-modified insulin signaling. *iScience* **24**, 102724 (2021).
2. Kovacheva, V. N., Khan, A. M., Khan, M., Epstein, D. B. A. & Rajpoot, N. M. DiSWOP: a novel measure for cell-level protein network analysis in localized proteomics image data. *Bioinformatics* **30**, 420–427 (2014).
3. Schubert, W. *et al.* Analyzing proteome topology and function by automated multidimensional fluorescence microscopy. *Nat Biotechnol* **24**, 1270–1278 (2006).
4. Moon, Y.-I., Rajagopalan, B. & Lall, U. Estimation of mutual information using kernel density estimators. *Phys Rev E* **52**, 2318–2321 (1995).
5. Reshef, D. N. *et al.* Detecting Novel Associations in Large Data Sets. *Science (1979)* **334**, 1518–1524 (2011).
6. Székely, G. J. & Rizzo, M. L. BROWNIAN DISTANCE COVARIANCE. *Ann Appl Stat* **3**, 1236–1265 (2009).
7. Gretton, A., Bousquet, O., Smola, A. & Schölkopf, B. Measuring Statistical Dependence with Hilbert-Schmidt Norms. in *Algorithmic Learning Theory* (eds. Jain, S., Simon, H. U. & Tomita, E.) 63–77 (Springer Berlin Heidelberg, Berlin, Heidelberg, 2005).
8. Hallac, D., Park, Y., Boyd, S. & Leskovec, J. Network Inference via the Time-Varying Graphical Lasso. in *Proceedings of the 23rd ACM SIGKDD International Conference on Knowledge Discovery and Data Mining* 205–213 (Association for Computing Machinery, New York, NY, USA, 2017). doi:10.1145/3097983.3098037.
