# Supplementary material for: Protein covariation networks for elucidating ferroptosis inducer mechanisms and potential synergistic drug targets
Source: Commun Biol. 2025 Mar 31;8:480. doi: 10.1038/s42003-025-07886-3 (PMC11958834; doi:10.1038/s42003-025-07886-3)
Supplement: Supplementary file 2 — Description of Additional Supplementary Files [file 42003_2025_7886_MOESM2_ESM.pdf]

Description of Additional Supplementary Files

File name: Supplementary Data 1
Description: List of antibodies

File name: Supplementary Data 2
Description: List of feature quantities

File name: Supplementary Data 3
Description: List of regents and resources

File name: Supplementary Data 4
Description: Source data used for the graphs presented in the main figures

File name: Supplementary Video S1
Description: Time-lapse observation of AX-53802–treated cells. Cells were prestained with the mitochondrial membrane potential probe MT-1 (red) and incubated in a medium containing SYTOX Green (green). AX-53802 treatment led to cell rounding starting from 1 h post-treatment, with some cells undergoing cell death as early as 3 h post-treatment. By 18 h, most cells underwent cell death, whereas some remained viable, highlighting varied responses to ferroptosis induction. Cell death was characterized by cellular swelling and rupture was observed, alongside loss of mitochondrial membrane potential.

File name: Supplementary Video S2
Description: Time-lapse observation of negative control cells. Cells were prestained with the mitochondrial membrane potential probe MT-1 (red) and incubated in a medium containing SYTOX Green (green). In the absence of AX-53802 treatment, mitochondrial membrane potential remained intact, and no cell death was observed.

File name: Supplementary Video S3
Description: Time-lapse observation of HEK293 cells stably expressing GFP-GPX4 and treated with 1.6 μM AX-53802. Membrane translocation of GPX4 was observed 5–10 min after adding AX-53802. Scale bar: 10 μm.

File name: Supplementary Video S4
Description: Time-lapse observation of HEK293 cells stably expressing GFP-GPX4 and untreated (negative control: no AX-53802 treatment). Scale bar: 10 μm.

File name: Supplementary Video S5

Description: Time-lapse observation of pulse-chase experiments using fluorescently labeled transferrin (AX-53802–treated condition). 1 HEK293 cells were incubated for 30 min with 10 μg/mL transferrin-AF488 (green) and Hoechst (blue) to facilitate transferrin-AF488 uptake. Subsequently, cells were chased with medium containing 1.6 μM AX-53802, and time-lapse images were captured over a chase period of 0–60 min. SPY555-FastAct (orange) was included in the chase medium to stain f-actin. Transferrin recycling was significantly delayed in the presence of AX-53802, with labeled transferrin persisting near the nucleus after 60 min, contrasting with rapid recycling observed under control conditions (Supplementary Video S6). Scale bars: 20 μm.

File name: Supplementary Video S6

Description: Time-lapse observation of pulse-chase experiments using fluorescently labeled transferrin (negative control). HEK293 cells were incubated for 30 min with 10 μg/mL transferrin-AF488 (green) and Hoechst (blue) to facilitate transferrin-AF488 uptake. Subsequently, cells were chased with medium containing AX-53802, and time-lapse images were captured over a chase period of 0–60 min. SPY555-FastAct (orange) was included in the chase medium to stain f-actin. Rapid transferrin recycling was observed under the negative control condition. Scale bars: 20 μm
