# Supplementary material for: Protein covariation networks for elucidating ferroptosis inducer mechanisms and potential synergistic drug targets
Source: Commun Biol. 2025 Mar 31;8:480. doi: 10.1038/s42003-025-07886-3 (PMC11958834; doi:10.1038/s42003-025-07886-3)
Supplement: Supplementary file 13 — Reporting Summary [file 42003_2025_7886_MOESM13_ESM.pdf]

nature portfolio

Corresponding author(s): Fumi Kano

Last updated by author(s): Feb 27, 2025

Reporting Summary

Nature Portfolio wishes to improve the reproducibility of the work that we publish. This form provides structure for consistency and transparency in reporting. For further information on Nature Portfolio policies, see our Editorial Policies and the Editorial Policy Checklist.

Statistics

For all statistical analyses, confirm that the following items are present in the figure legend, table legend, main text, or Methods section.

n/a	Confirmed	
☐	☒	The exact sample size (n) for each experimental group/condition, given as a discrete number and unit of measurement
☐	☒	A statement on whether measurements were taken from distinct samples or whether the same sample was measured repeatedly
☐	☒	The statistical test(s) used AND whether they are one- or two-sided<br>*Only common tests should be described solely by name; describe more complex techniques in the Methods section.*
☒	☐	A description of all covariates tested
☒	☐	A description of any assumptions or corrections, such as tests of normality and adjustment for multiple comparisons
☐	☒	A full description of the statistical parameters including central tendency (e.g. means) or other basic estimates (e.g. regression coefficient) AND variation (e.g. standard deviation) or associated estimates of uncertainty (e.g. confidence intervals)
☐	☒	For null hypothesis testing, the test statistic (e.g. F, t, r) with confidence intervals, effect sizes, degrees of freedom and P value noted<br>*Give P values as exact values whenever suitable.*
☒	☐	For Bayesian analysis, information on the choice of priors and Markov chain Monte Carlo settings
☒	☐	For hierarchical and complex designs, identification of the appropriate level for tests and full reporting of outcomes
☐	☒	Estimates of effect sizes (e.g. Cohen's d, Pearson's r), indicating how they were calculated

Our web collection on statistics for biologists contains articles on many of the points above.

Software and code

Policy information about availability of computer code

Data collection	NIS-Elements ver 5.3 was used to obtain fluorescence images. Gen5 ver 3.09 was used for measuring CellTiter-Glo 2.0 Cell Viability Assay.
Data analysis	R ver 4.2.1 and Rstudio version 2022.07.1+554 was used for data analysis.

For manuscripts utilizing custom algorithms or software that are central to the research but not yet described in published literature, software must be made available to editors and reviewers. We strongly encourage code deposition in a community repository (e.g. GitHub). See the Nature Portfolio guidelines for submitting code & software for further information.

Data

Policy information about availability of data

All manuscripts must include a data availability statement. This statement should provide the following information, where applicable:

- Accession codes, unique identifiers, or web links for publicly available datasets
- A description of any restrictions on data availability
- For clinical datasets or third party data, please ensure that the statement adheres to our policy

The dataset generated during and/or analyzed during the current study are available from the corresponding author on reasonable request.

Research involving human participants, their data, or biological material

Policy information about studies with human participants or human data. See also policy information about sex, gender (identity/presentation), and sexual orientation and race, ethnicity and racism.

Reporting on sex and gender	Not associated for this study.
Reporting on race, ethnicity, or other socially relevant groupings	Not associated for this study.
Population characteristics	Not associated for this study.
Recruitment	Not associated for this study.
Ethics oversight	Not associated for this study.

Note that full information on the approval of the study protocol must also be provided in the manuscript.

Field-specific reporting

Please select the one below that is the best fit for your research. If you are not sure, read the appropriate sections before making your selection.

☒ Life sciences ☐ Behavioural & social sciences ☐ Ecological, evolutionary & environmental sciences

For a reference copy of the document with all sections, see nature.com/documents/nr-reporting-summary-flat.pdf

Life sciences study design

All studies must disclose on these points even when the disclosure is negative.

Sample size	For Western blotting and rt-PCR, the sample sizes were selected according to customary practices in the field, with a minimum of three independent biological replicates per condition. In constructing the covariation network through image analyses, we generally utilized datasets from more than 1,000 cells in each condition.
Data exclusions	Automatic image acquisition can occasionally produce images that are either unfocused or contain large fluorescent aggregates. Whenever the quantification data for a particular feature is significantly deviated, we reviewed the original images and excluded any outliers.
Replication	Western blotting and rt-PCR analyses were replicated and all replication attempts were successful.
Randomization	To reduce arbitrariness, we automatically acquired cell images at n×n grid points per well, utilizing A1 confocal microscopy controlled by NIS-Elements software (Nikon).
Blinding	In the PLOM-COM analysis, the target protein names were converted to numbers after sample preparation, and were converted back to their original names after creating the network. Additionally, the conditions names were kept confidential and were numerically coded during image acquisition and analysis.

Reporting for specific materials, systems and methods

We require information from authors about some types of materials, experimental systems and methods used in many studies. Here, indicate whether each material, system or method listed is relevant to your study. If you are not sure if a list item applies to your research, read the appropriate section before selecting a response.

Materials & experimental systems

n/a	Involved in the study	
☐	☒	Antibodies
☐	☒	Eukaryotic cell lines
☒	☐	Palaeontology and archaeology
☒	☐	Animals and other organisms
☒	☐	Clinical data
☒	☐	Dual use research of concern
☒	☐	Plants

Methods

n/a	Involved in the study	
☒	☐	ChIP-seq
☒	☐	Flow cytometry
☒	☐	MRI-based neuroimaging

Antibodies

Antibodies used	SLC7A11, Invitrogen, Cat#PA5-116134<br>SLC3A2, Sigma, Cat#HPA017980<br>GPx4, abcam, Cat#ab125066, clone EPNCIR144<br>ATF4, CST, Cat#11815S, clone D4B8<br>HSPA5 (BiP, GRP78), abcam, Cat#ab21685<br>NRF2, MBL, Cat#M200-3, clone 1F2<br>AIFM2 (FSP1), Sigma, Cat#HPA028961<br>DHODH, Sigma, Cat#HPA010123<br>FATP2 (SLC27A2), Invitrogen, Cat#PA5-102343<br>TFR1, Invitrogen, Cat#13-6800, clone H68.4<br>HSPB1, e Bioscience, Cat#14-9112-80, clone STRSN<br>FT, Invitrogen, Cat#MA5-14736, clone 513C10<br>HIF1a, abcam, Cat#ab179483, clone EPR16897<br>NCOA4, Sigma, Cat#HPA051260<br>ATG7, abcam, Cat#ab201251, clone EP1759Y<br>HSP90α, Invitrogen, Cat#MA5-25036, clone OTI3B5<br>KEAP1, CST, Cat#8047, clone D6B12<br>HO-1, abcam, Cat#ab214643, clone EPR1390Y<br>NF2 (merlin), Sigma, Cat#HPA003097<br>LATS1, MyBioSource, Cat#MBS9610532<br>YAP1, CST, Cat#14729, clone D8H1X<br>LSH (HELLS), Sigma, Cat#HPA063242<br>EGLN1, Invitrogen, Cat#PA5-78511<br>4HNE, JalCA, Cat#MHN-020P, clone HNE-J2<br>ACSL4, Invitrogen, Cat#PA5-100033<br>Myosin IIB, CST, Cat#8824, clone D8H8<br>Beta tubulin, SIGMA, Cat#T8328, clone AA2<br>pFAK, abcam, Cat#ab81298, clone EP2160Y<br>FAK, Invitrogen, Cat#39-6500, clone ZF002<br>LAMP2, Hybridoma Bank, Cat#H4B4-c (Clone ID)<br>mTOR, CST, Cat#2983, clone 7C10<br>pAKT, Merck, Cat#05-1003, clone 6F5<br>pRPS6, CST, Cat#4858, clone D57.2.2E<br>TFEB, CST, Cat#4240<br>GAPDH (for IF), abcam, Cat#ab83956<br>Alexa-488-anti-mouse IgG, molecular probes, Cat#A11029<br>Alexa-546-anti-mouse IgG, molecular probes, Cat#A11030<br>Anti-Chicken IgY, Cy5, abcam, Cat#ab97147<br>anti-Rabbit IgG, Alexa Fluor 488, abcam, Cat#ab150061<br>Anti-Mouse IgG, Alexa Fluor 647, abcam, Cat#ab150111<br>pSTAT3(Y705), CST, Cat#9145, clone D3A7<br>STAT3, CST, Cat#9139, clone 124H6<br>GAPDH (for WB), Millipore, Cat#MAB374, clone 6C5
Validation	The antibodies used in this study have been validated by manufacturers, and the details are demonstrated on the product websites.

Eukaryotic cell lines

Policy information about cell lines and Sex and Gender in Research

Cell line source(s)	HEK293 cells and HeLa cells were obtained from an existing collection at the Kano Laboratory, Tokyo Institute of Technology. SW480 and HT29 cells were purchased from the American Type Culture Collection (Manassas, VA, USA), HCT-116 cells were obtained from the European Collection of Authenticated Cell Cultures (Porton Down, Salisbury, UK), and HT-1080 cells were acquired from the JCRB.
Authentication	No authentication was performed by the authors of this manuscript.
Mycoplasma contamination	Cell line was not tested for mycoplasma contamination but no indication of contamination was observed.
Commonly misidentified lines<br>(See ICLAC register)	No commonly misidentified lines are used.

Plants

Seed stocks	N/A
Novel plant genotypes	N/A
Authentication	N/A
